# Block-Based Development of Mobile Learning Experiences for the Internet of Things

**DOI:** 10.3390/s19245467

**Published:** 2019-12-11

**Authors:** Iván Ruiz-Rube, José Miguel Mota, Tatiana Person, José María Rodríguez Corral, Juan Manuel Dodero

**Affiliations:** School of Engineering, University of Cádiz, Avenida de la Universidad de Cádiz, 10, 11519 Puerto Real, Cádiz, Spain; josemiguel.mota@uca.es (J.M.M.); tatiana.person@uca.es (T.P.); josemaria.rodriguez@uca.es (J.M.R.C.); juanma.dodero@uca.es (J.M.D.)

**Keywords:** Internet of Things (IoT), mobile apps, end-user development, App Inventor, block-based languages, map-reduce

## Abstract

The Internet of Things enables experts of given domains to create smart user experiences for interacting with the environment. However, development of such experiences requires strong programming skills, which are challenging to develop for non-technical users. This paper presents several extensions to the block-based programming language used in App Inventor to make the creation of mobile apps for smart learning experiences less challenging. Such apps are used to process and graphically represent data streams from sensors by applying map-reduce operations. A workshop with students without previous experience with Internet of Things (IoT) and mobile app programming was conducted to evaluate the propositions. As a result, students were able to create small IoT apps that ingest, process and visually represent data in a simpler form as using App Inventor’s standard features. Besides, an experimental study was carried out in a mobile app development course with academics of diverse disciplines. Results showed it was faster and easier for novice programmers to develop the proposed app using new stream processing blocks.

## 1. Introduction

The Internet of Things (IoT) concept has several definitions, as involved technologies are continually evolving. IoT is defined as “a network that connects uniquely identifiable things to the Internet” [1]. These *things* have sensing and actuating capabilities and can be programmed, such that data can be collected and their state can change. IoT potentialities enable the development of a significant number of applications for improving citizens’ life. Smart homes and buildings, smart cities, mobility and transportation, healthcare, agriculture and industry are some of the main areas of IoT application [1]. For a rapid materialization of IoT, the symbiosis among the physical world and the cyber world must be harmonious [2]. Interactions between humans and computing-enabled objects must be smarter and opportunistic [3]. As it may happen with humans’ intelligence [4], the smartness of IoT things relies heavily on their sensory, interactive capabilities. In this vein, smart interactive objects enable creating tangible things to do different tasks in different application domains [5].

The development of smart IoT applications usually requires strong programming skills, which commonly exceed people’s abilities. However, in recent years, several projects, such as Arduino and Raspberry Pi, aimed not only at professionals but also educators and students, have influenced the IoT expansion. These initiatives include both hardware platforms and programming tools, and a user community is growing around them.

Since the notation used in programming languages has a tremendous impact on novices [6], various tools to program IoT microcontrollers and microcomputers have emerged. These tools are based on block-based languages and proved to be useful for novice programmers. Learners of block-based languages depicted greater gains in algorithmic thinking [7] and a higher interest in computer science than those using text-based environments [8]. Differences between block-based languages and text-based languages often fade after learners transfer their acquired knowledge of computer programming to more professional, text-based languages and environments [9].

Currently, the most commonly used block-based programming tools, namely Scratch and App Inventor, provide capabilities to connect with external hardware devices, such as Arduino. However, they present some limitations when it comes to developing IoT applications, namely: (1) the absence of an easy mechanism for ingesting and processing event data streams and (2) the lack of usable mechanisms for visually representing data.

To facilitate authoring of IoT mobile apps, several visual components for a custom version of App Inventor, as well as a set of extensions for its block-based programming language, have been developed. With these components and language extensions, users can easily create apps that ingest data streams from available sensors, process them using a map-reduce programming style and then visualise the results of data processing graphically. The goal of this paper is to investigate how easy it is for non-experts to leverage such improved features to create their own smart IoT applications.

The block-based language approach followed in our research proposal has some limitations, which have been described in the literature. First, it may be applicable only for novice programmers who are learning to create their own smart IoT applications [9]. The research claims and results are not directly transferable to professional, text-based programming languages or even to other not block-based, visual programming paradigms [10]. Second, the use of programming concepts that are relevant to create smart IoT applications (such as state initialisation [11], parallelism [12], anonymous functions [13] and higher-order functions [14]) were adapted to visual and block-based languages. However, there are no evidences of learning improvements thanks to the use of such end-user development (EUD) approaches. Therefore, the use of block-based languages as an EUD approach for creating smart IoT applications may have limitations, which have to be overcome by more extensive research, as intended in this work.

The rest of the paper is structured as follows: the background and related works are presented in Section 2. Section 3 describes the main contribution. Two case studies are included in Section 4 and Section 5. The former presents a usability study conducted with students of a computer programming fundamentals course whereas the latter was targeted at university lecturers. Finally, Section 6 discusses the results and draws the conclusions of this research.

## 2. Background & Related Works

IoT solutions are composed of hardware and software elements. Guth et al. [15] propose an IoT reference architecture from a comparison of various open-source (SiteWhere, OpenMTC and FIWARE) and proprietary (Amazon Web Services IoT) IoT platforms. Such architecture includes a set of sensors and actuators at the lower level. On the next level up, a hardware device is connected by a wired connection or wirelessly to sensors and actuators. Data communication protocols are required to manage the constraints of the smart devices, as well as gateways to translate data between different protocols and to forward communications. Middleware [15,16] processes the data received from the connected devices (e.g., by the execution of condition-action rules) to provide them to connected applications and sends commands to be executed by the corresponding actuators. Finally, IoT applications allow device-to-device and human-to-device interactions [17]. In the latter, mobile app-based smart interactive experiences can be provided for end-users.

Existing initiatives for learning and developing IoT solutions as well as block-based end-user development tools and their applications for creating IoT mobile experiences are described below.

### 2.1. Initiatives for Learning and Developing IoT Solutions

Arduino and Raspberry Pi are some of the most popular platforms used for educational purposes [1,18,19], with a huge development community. Arduino is a programmable circuit board, which can be connected with sensors and actuators of many types. Raspberry Pi is a single-board computer to run programs in a multitasking environment. However, the analog-digital conversion is not available onboard and thus additional hardware is required for interfacing with analog sensors such as photocells, joysticks and potentiometers.

Some initiatives and educational projects were carried out in order to teach IoT technologies for undergraduate and university students [20,21]. For example, in a project-based teaching and learning approach conceived for an IoT course [22], Raspberry Pi is used to devise and implement IoT designs. Other project-based learning courses for learning wired and wireless networking techniques have been offered to electrical and computer engineering students [23]. The use of microcontrollers with network connectivity and without complex operating systems provides cost-effective, well-supported and flexible platforms for developing IoT applications.

Moreover, the educational research outcome of teaching IoT device prototyping in a practical, real problem-based setting is presented [24] as a means for teaching computer science and software engineering. An example course outline for planning learning experiences in IoT prototyping is described along with a general assessment framework and best practice recommendations in order to facilitate personalised learning in analogous contexts.

Some educational approaches are based on the pocket labs (PL) concept to stimulate students’ initiative and creativity. PL allow learners to experiment with real equipment in any place and at any time [25]. Despite that IoT and PL are not initially interrelated, the authors present a real case of IoT teaching practice based on Arduino and accompanying shields that includes sensors and actuators. PLs are combined with the online Tinkercad software tool to prototype and simulate electronic designs that include the Arduino boards.

Other initiatives for integrating IoT technologies in existing teaching-learning case studies were developed. For example, an IoT-based learning framework that integrates IoT and hardware/software technologies is used as part of a software engineering course for embedded system analysis and design [26]. Specifically, the authors introduced a lab development kit composed by Arduino and Raspberry Pi boards, sensors and XBee modules for providing wireless communication.

Common general-purpose programming languages can be used for developing IoT applications [19]. However, since IoT systems involve a wide variety of hardware and software components, depending on a range of distributed system and communication technologies, developing IoT applications is time-consuming and complex. Hence, a variety of IoT libraries, such as CoAPthon [27], and frameworks [28], such as IDeA, FRASAD, D-LITe, IoTLink, WebRTC based IoT application Framework, Datatweet, IoTSuite and RapIoT, have been developed to manage those complexities.

### 2.2. End-User Development Tools for IoT

Modern software programming tools hide much of the complexity of traditional programming languages. Recent *low code* software engineering approaches have been successful both for IoT [29] and for more general mobile application development [30]. Their general objective consists in making application creation easier for people without programming skills. This goal is shared by the research field known as end-user development (EUD). A recent review on this topic differentiates between end-user programming (EUP) and other software engineering activities that span the entire software development lifecycle [31]. The review was recently completed by another author, focusing on current EUD tools for developing IoT and robot applications [32].

Among EUP tools, block-based programming environment features are noteworthy [33] to enable composing programs without dealing with the syntactic issues of textual languages. Among such block-based languages and environments, Scratch [34,35] is very popular to create interactive games, stories and animations, as well as to share such creations on the Web. Scratch computer programs are built by dragging and dropping blocks that represent common programming elements, such as variables, expressions, conditions and statements. Another block-based EUP approach for robotic applications is Phratch, which is a Scratch-like live programming environment [36]. Besides, App Inventor [37,38] is an open-source block-based programming tool. This tool enables users without prior programming experience to create apps specifically for smartphones and mobile devices. In particular, it makes mobile app deployment easier for the end-user. Additionally to other tools’ amenities, App Inventor allows end-users to perform interface design and software deployment tasks, which belong to the realm of EUD beyond EUP. End-users can drag, drop and arrange various interface and non-visible components through a visual designer and then use a block language editor to program the app logic behaviour in order to create and deploy fully functional mobile apps. App Inventor provides event handling as a form of trigger-action programming (TAP), which proved to be particularly suitable to define bespoke behaviours to respond to the multiple events that may occur in an IoT context [39]. End-users specify the behaviour of a system as events or triggers and response actions when the events occur [40].

Despite the availability of libraries and frameworks to work with IoT technologies, it is very complicated to find EUD solutions to assist non-IT professionals in a particular area or topic to develop their own IoT consumer applications and smart user experiences. For example, ScratchX [41] is an experimental platform that allows people to test experimental functionalities built by some developers for the Scratch visual language. These experimental extensions enable apps to integrate with web services and external hardware, such as Arduino or Raspberry Pi.

On the other hand, the MIT IoT App Inventor project [42] allows students, teachers and *makers* to implement IoT projects in the same way as they develop regular mobile apps. This project provides users with components and block extensions to read data from a great variety of sensors (e.g., moisture, pressure, temperature, noise, etc.) and control a multiplicity of actuators (e.g., buzzers, lights, motors, etc.) As apps run on mobile devices, they can take advantage of all built-in features provided by App Inventor, but they can also use the apps to interact with objects all around. Besides, UDOO [43] is a combined set of open hardware and software technologies to allow novice makers to create their own digital objects connected to the cloud and to define custom behaviour logic for sensors and actuators. In addition to the physical devices, UDOO includes an App Inventor extension to handle sensors and actuators from within mobile apps. Finally, IoT Inventor [44] is a web-based integration platform, not based on but inspired by App Inventor, with a friendly drag-and-drop composer interface to build personalised and reconfigurable services using smart IoT-enabled things.

All of the described extensions are targeted to handle sensors and actuators but they do not provide support for easily ingesting, processing and visualizing data.

## 3. Creating IoT Mobile Apps with VEDILS

VEDILS [45] is a visual environment for designing interactive learning scenarios. It is an authoring tool targeted at users without programming skills who want to create their own mobile apps. The platform is based on App Inventor, the programming tool to build apps for mobile devices. The current version requires Android devices, though an iOS-based version is currently being devised by MIT. The development environment relies on the Blockly library for its visual programming language based on blocks.

App Inventor provides several components for designing mobile apps’ user interfaces as well as other features, including multimedia elements, communication with the device sensors, sharing through social networks, etc. In addition to the built-in components provided by App Inventor, VEDILS features new components to enrich the apps with virtual and augmented reality experiences and to serve multi-modal external Human Machine Interface (HMI) devices, such as hand gesture sensors or electroencephalography (EEG) headsets, among other features. The platform was also used to conduct a study on the suitability of visual languages for non-expert robot programmers [46]. Regarding IoT computing, several components and blocks were developed for VEDILS to ingest, process and visualise data from a diversity of sensors.

### 3.1. Ingesting IoT Data Streams

App Inventor manages the following block types for each component: property getters and setters (green blocks), functions (blue blocks) and event handlers (yellow blocks). VEDILS extends those with a particular type of block (similar to event handlers) for non-visual components that issue a continuous flow of data, as is the case of both internal and external sensors. These kinds of components (red blocks) provide the app developer with a data stream suitable to be treated with the processing blocks described in Section 3.2; these are triggered when data from the sensor are ingested for a predefined time window.

**Figure 1:**
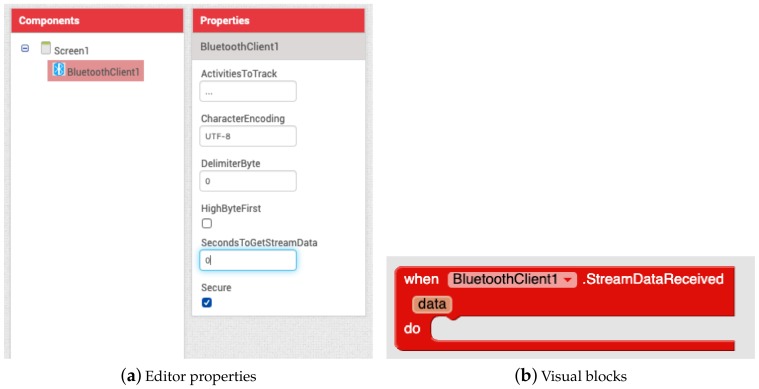
Ingesting stream data from Bluetooth external devices.

One of the most common ways of receiving data from an IoT sensor and sending commands to an actuator is via a Bluetooth connection. Thus, the built-in *BluetoothClient* component was extended with the new *StreamDataReceived* block (see Figure 1), which provides the data stream as well as a new *SecondsToGetStreamData* property to set the time period to fetch new data from the Bluetooth server.

**Figure 2:**
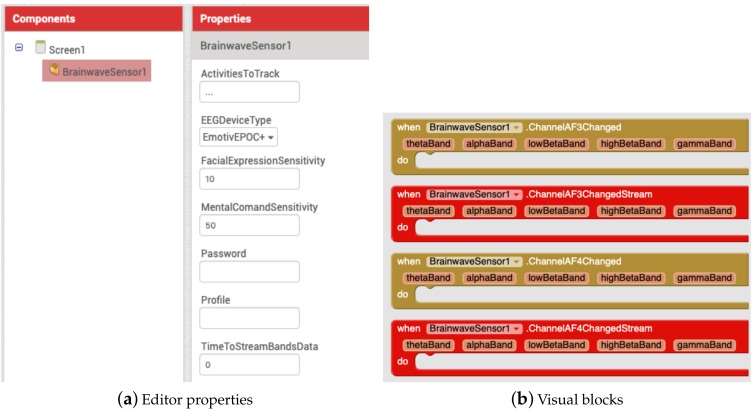
Ingesting stream data from electroencephalography (EEG) headsets.

In addition, every new VEDILS component that provides communication with internal or external devices can support the streaming blocks. For example, the *BrainwaveSensor* component, which enables to detect brain activity by means of an EEG headset, includes specific blocks for ingesting stream data from regular fast Fourier transform (FFT) bands (i.e., Theta, Alpha, Low Beta, High Beta and Gamma) of EEG channels (see Figure 2). It also includes a *TimeToStreamBandsData* property to set the time window. The current implementation works with the Emotiv Epoc+ and Insight headsets. Thus, this component enables a new range of mobile applications to monitor emotions, track cognitive performance and even control objects through learning a set of mental activity patterns that can be trained and interpreted as mental commands.

The blocks of Figure 2 were developed as Java class methods that support external and internal sensors. The data flow is internally managed as Java 8 streams. Besides, additional classes based on threads and the Java Timer API were required to periodically check data availability.

### 3.2. Processing IoT Data Streams

**Figure 3:**
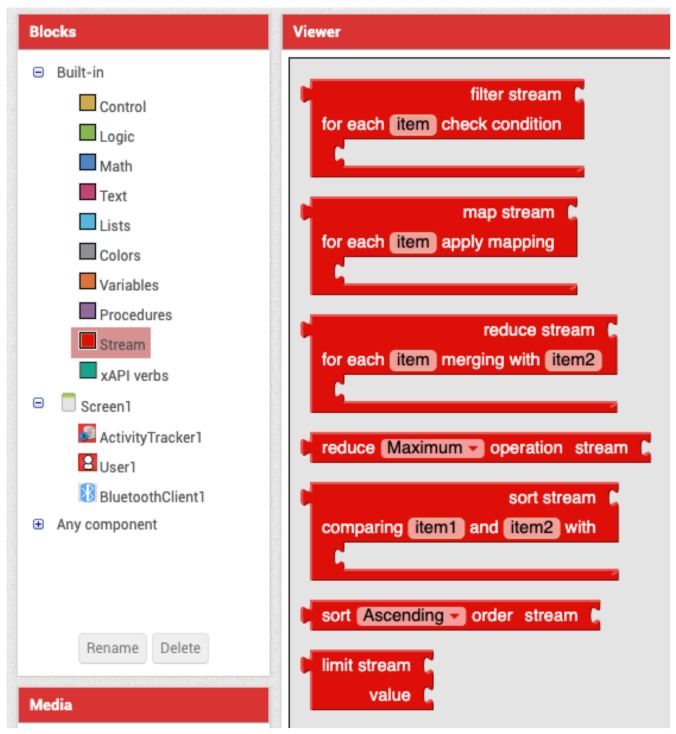
Visual blocks for processing stream data.

In order to address the issue of treating IoT data, several data processing blocks were developed and delivered with VEDILS (see Figure 3):

- *Filter* block: removes the elements that do not meet a specific condition from an input stream. For example, in a stream containing a set of numbers, developers could filter the odd numbers obtaining a new stream with only the even ones.
- *Map* block: applies an operation to each element of an input stream. For example, transforming a stream of lowercase words into a stream of uppercase words.
- *Reduce* (a) block: combines the elements contained in the input stream by applying the binary operator specified as a parameter. The combiner function must combine two numbers to return a new one, such as the maximum or minimum value.
- *Reduce* (b) block: combines the elements contained in the input stream, by applying one of the built-in mathematical operations. For example, computing the average or standard deviation of a 50-item stream.
- *Sort* (a) block: produces a new stream with the elements of the input stream according to the order induced by the comparator specified as a parameter. The comparator must be a function that returns a negative number if *item1* is less than *item2*; a positive one if *item1* is higher than *item2*; or zero if both items are equal.
- *Sort* (b) block: produces a new stream with the elements of the input stream according to its natural order, i.e., numerically or alphabetically. The block has a field to specify whether to apply an ascending or descending sorting.
- *Limit* block: shortens the stream size to the specified length. For example, collecting the first 10 items in the stream.

All the previous blocks are intermediary operations, except for the *Reduce* block, which is terminal. The intermediary blocks can be indistinctly chained, whereas the *Reduce* blocks must always appear on the left of the sequence of operations.

**Figure 4:**
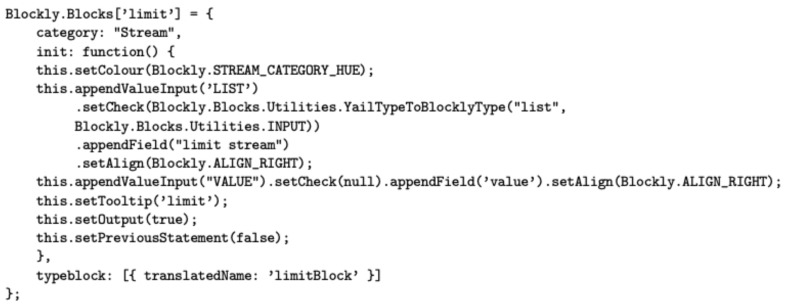
JS fragment for defining the *Limit* block with Blockly.

**Figure 5:**
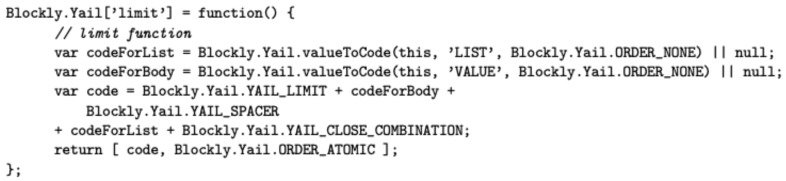
JS fragment for generating the Young Android Intermediate Language (YAIL) code of the *Limit* block.

Extensions for the visual programming language itself requires not only Java code but also other languages. The visual appearance of each block of the *Streams* palette is defined by a JavaScript fragment using the Blockly library API, whereas its run-time behaviour is defined by generating YAIL code. Young Android Intermediate Language (YAIL) is a set of abstractions for Kawa, a Java-based implementation of the Scheme functional language. Figure 4 and Figure 5 show the code required to develop the *limit* block.

### 3.3. Visualising IoT Data Streams

**Figure 6:**
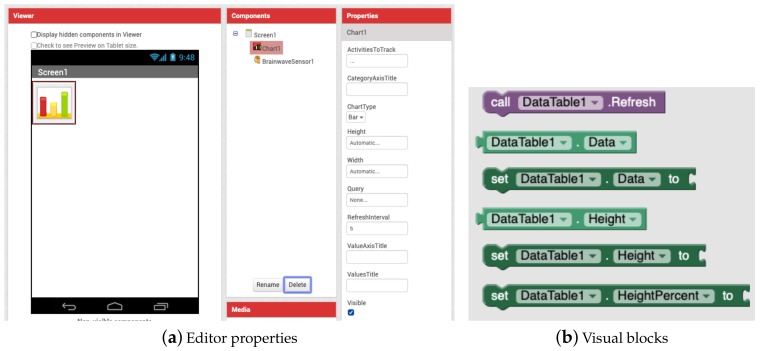
Visualizing stream data with the chart component.

App Inventor does not provide built-in capabilities to include charts or data tables in the apps. Thus, two new visible components were integrated into VEDILS for allowing developers to equip their apps with those kinds of visualisations (see Figure 6). The *Chart* component enables the creation of simple graphics such as bars, lines or pie charts, whereas the *DataTable* component is intended to present the data in a tabular format. Both components can be fed with a data stream from any sensor and they are customisable, for example, by configuring the category and value axes of charts.

Both the *Chart* and *DataTable* components were developed as Java classes that inherit from the *AndroidViewComponent* superclass, already included in App Inventor. In run-time, these components provide an embedded *WebViewer* for the app screen, which points to an external HTML. That web page receives a JSON string containing the app data and renders it via the Google Chart API.

## 4. Evaluating IoT Mobile App Development with Students

This section presents a usability study of the VEDILS components for IoT computing. This test is aimed at checking whether these IoT components are suitable for learners when programming end-user IoT mobile apps. The test was defined and executed by following the guidelines provided by Rubin et al. [47].

### 4.1. Study Design

**Figure 7:**
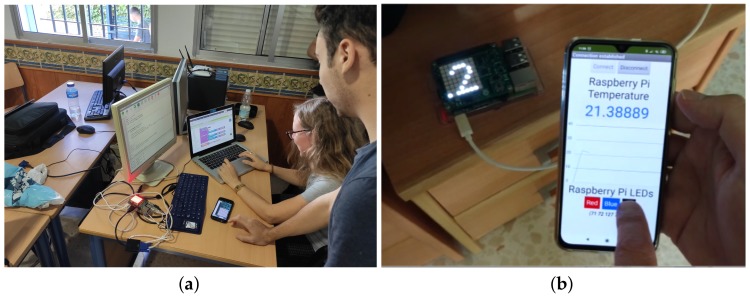
Developing the mobile app for interacting with the external Internet of Things (IoT) device. (**a**) Students programming the mobile app; (**b**) Android app communicating with the Raspberry and its Sense HAT add-on.

A three-hour workshop was conducted with students of a computer programming fundamentals course from a vocational education and training module. This workshop was implemented in the frame of a Code Week initiative (https://codeweek.eu/view/242496/desarrollo-sencillo-de-apps-moviles-para-iot). The research question for this study was the following: is it easier for students to develop IoT mobile apps using VEDILS than using App Inventor?. To evaluate the legibility degree and ease of use of the IoT blocks compared to App Inventor’s, we designed the following experimental scenario: During the first hour, students learned about IoT, its basic concepts, components and architectures. Later on, they learned about the features and applications of the Raspberry Pi single-board computer and Sense HAT add-on. Then, they were presented with the running IoT sample app described below. The instructor taught students how to design the UI of the app as well as the blocks required to connect and disconnect the Raspberry Pi device through Bluetooth. Later on, the students were provided with a base project for both App Inventor and VEDILS to be completed during the rest of the session (see Figure 7a). Finally, they were asked to fill out a short questionnaire, including both quantitative and qualitative question items.

A quasi-experimental study was conducted with the students. One half of the participants created first the app with App Inventor and then with VEDILS; whereas the other half did it in reverse order. To complete the app projects, the students were also provided with step-by-step tutorials to guide the development with both tools.

### 4.2. The Sample App

The proposed app shows the average temperature received from the IoT sensor for the last 10 s. In order to bring the IoT app development closer to a more realistic scenario, the temperature measurements of the Raspberry Pi were generated from a simulation server. The python server running in the device generated random value series in the [14, 104] interval, measured as degrees Fahrenheit. Occasionally, abnormal values (i.e., 127°F) were generated to emulate measurement errors. The app had to obtain the temperature data, convert it into Celsius degrees, remove outliers and compute the average of the series.

#### 4.2.1. User Interface Design

The app layout (see the picture in Figure 7b) is based on a vertical arrangement composed of several elements. At the top of the screen, there are buttons for connecting and disconnecting to/from the Raspberry Pi. A chart depicting the evolution of the temperature is included in the centre of the screen. At the bottom of the screen, there are buttons for sending commands to change the Sense HAT LED panel background colour. The LED panel in the picture shows the temperature measured every second.

From the user interface perspective, the only difference between the App Inventor and VEDILS versions is that the former requires a *Canvas* component, whereas the latter uses the new *Chart* component. Nevertheless, from the user programming perspective, there are some remarkable differences, as explained next.

#### 4.2.2. Programming with App Inventor

**Figure 8:**
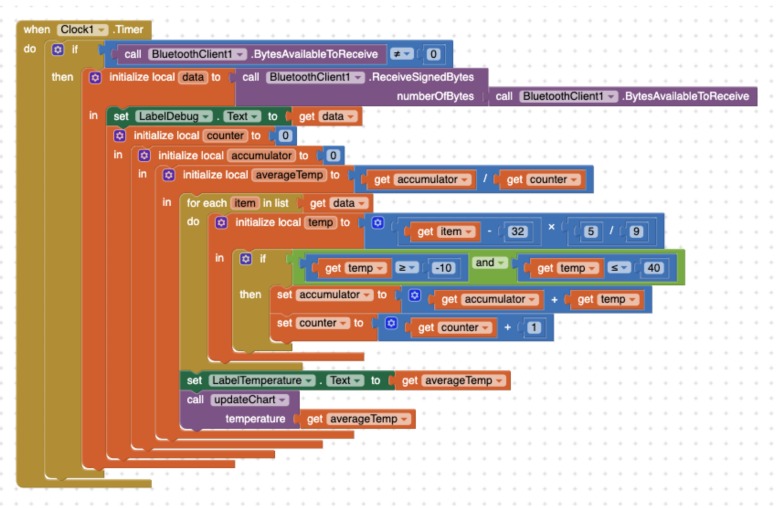
Processing temperature data with App Inventor.

**Figure 9:**
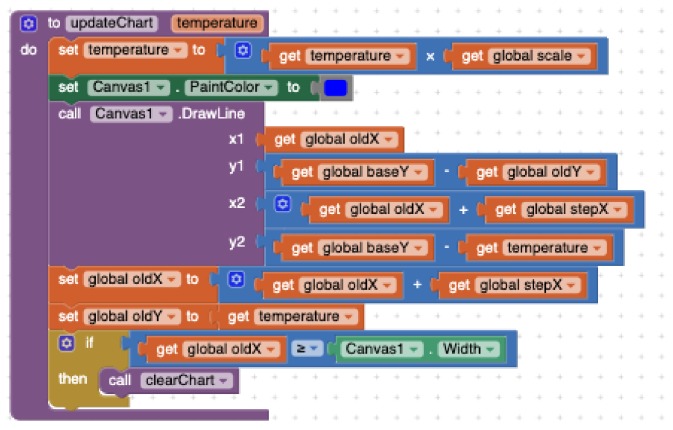
Drawing temperature data with App Inventor.

A *Clock* component must be used to periodically check if there is new data to receive via the Bluetooth connection. Then, the developer must call the *ReceiveSignedBytes* method and then iterate through the data collection. For each individual value, a local variable is used to store the result of applying the conversion formula between the two measurement scales. Later on, a conditional statement must be applied to check if the calculated value is not an outlier. If so, that value must be added to an *accumulator* variable and increment by one the *counter* of valid measurements. Later on, the accumulator variable must be divided by the counter to compute the average (see Figure 8). Two text labels are accordingly updated to show the received raw of data and the computed average. Finally, the *updateChart* procedure is called to update the visual representation. Figure 9 shows how the *DrawLine* block in the *Canvas* must be used to depict the temperature along time as a line chart. This block requires a pair of (x,y) coordinates for both source and target points. Since the (0,0) point of the Canvas corresponds with its left upper corner, it is necessary to turn the temperature values into the proper values for the Y-axis. Besides, the X-axis must be consequently moved forward for each time instant. Some other variables must also be used to control the coordinates. Furthermore, at the beginning of the drawing process and every time the canvas right-edge is reached, the drawing area must be cleared and some horizontal lines must be drawn to represent certain temperature milestones (0°, 10°, etc.). Besides, some variables must be reinitialised.

#### 4.2.3. Programming with VEDILS

**Figure 10:**
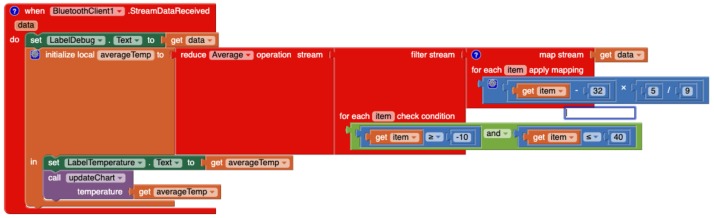
Processing temperature data with VEDILS.

**Figure 11:**
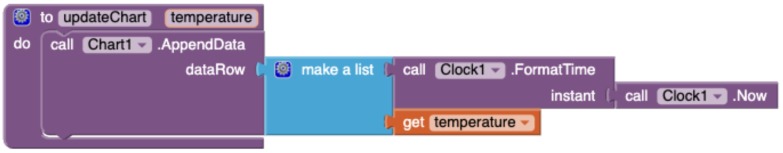
Drawing temperature data with VEDILS.

With VEDILS (see Figure 10), the developer must handle the *StreamDataReceived* block, which directly provides a data stream of temperature measurements. This data stream is pipelined through a series of processing steps. With the *Map* block, every item in the stream is mapped into its corresponding Fahrenheit value; with the *Filter* block, outliers are discarded according to the validity condition; and finally, with the *Reduce* block, the data stream is summarized by computing an average. The *AppendData* block is used to depict the average temperature along time in the line chart. Thus, the computed value must be sent to the *Chart* component, together with the current timestamp provided by *Clock*. Previously, the *Chart* must have been configured with the category and value axes (see Figure 11).

### 4.3. Data Compilation

The data collection was performed without interacting with the subjects during the experiment (i.e., an indirect method). The online questionnaire designed for the survey includes, in addition to the consent form, several questions to determine the initial status of participants as well as to compile the students’ opinions after the test. They were asked about their expertise level creating software programs with visual languages and with text-based programming languages. Regarding the post-test, some questions related to the perceived ease of use of App Inventor and VEDILS were included. They deal with the tool usability for (i) connecting/disconnecting via Bluetooth and sending commands to the IoT sensor; (ii) consuming temperature data from the sensor, applying a transformation for changing their measurement scale, removing the outliers and computing the average; and (iii) drawing a chart with the temperature evolution. The answers to these questions follow a five-level Likert scale (1-Strongly disagree, 2-Disagree, 3-Neither agree nor disagree, 4-Agree and 5-Strongly agree). The participants were also asked about their intention to use App Inventor or VEDILS to create more IoT projects. Study data as well as the resources used are linked in the Supplementary Materials.

### 4.4. Analysis and Findings

This study aimed at checking whether it is easier for students to develop the proposed IoT mobile app using VEDILS rather than with App Inventor. Ten students (eight men and two women) aged 24 (stddev = 2) participated in the study. All of them were first-year students of a vocational course in web development. By the time the workshop was conducted, they had not yet learned any textual programming tool. They had only studied and used the App Inventor platform. Only one of them had previous experience with traditional text-based coding languages.

All students agreed that it is easy (avg = 4.0, stddev = 0.0) to develop the routines for connecting/disconnecting via Bluetooth and sending commands to the IoT sensor with App Inventor and VEDILS (both tools share the same blocks for that purpose). Regarding the temperature data ingestion and processing steps, most students neither agree nor disagree (avg = 2.77, stddev = 0.40) that these steps are easy to develop with App Inventor. Nevertheless, most students agreed that it is easy to develop these routines with VEDILS (avg = 3.88, stddev = 0.26). This perceived ease of use is even more substantial when developing the temperature evolution chart: the App Inventor *Canvas* component (avg = 2.66, stddev = 0.44) versus the VEDILS *Chart* component (avg = 4, stddev = 0.23). Finally, regarding the question about which tool they would use to develop mobile apps that consume data from IoT sensors and depict them in a chart, 66.6% of the participants chose VEDILS, 11.1% chose App Inventor, whereas the rest did not indicate a preference. In short, all participants rated VEDILS better than App Inventor, except for the student who already had coding skills.

A total of 177 blocks were required for developing the App Inventor version of the app, whereas only 84 were required in VEDILS. In both cases, procedures were used to avoid as much as possible the number of duplicate blocks. Accordingly, the difference in the size of the projects may relate to the students’ perceived ease of use for both tools. That difference is particularly pronounced when it comes to presenting the temperature chart because developers must handle many details of the drawing process. The results are also consistent with the qualitative opinions expressed by the students, who highlighted the saving of programming effort required to consume, process and visualise IoT data thanks to the abstractions provided by VEDILS.

## 5. Evaluating VEDILS Data Processing Blocks with Academics

While the above section evaluates the components and language extensions provided by VEDILS for IoT computing, this case study solely focuses on the data processing blocks. The main objective is to check the development agility and the usability of the stream blocks compared to the standard built-in blocks for processing data. The design, implementation and analysis of the experimental study are presented below.

### 5.1. Study Design

The study was performed through six editions of an introductory course of mobile app development with App Inventor/VEDILS between January and February 2018. These courses are part of the Cádiz university’s docent innovation program, in which several IT-related courses are regularly delivered to their associated lecturers and researchers.

The reference framework for establishing the hypotheses of this study is based on the potential benefits of certain computer programming paradigms over others [48]. Some authors explored techniques for introducing parallelism concepts, anonymous procedures and higher-order functions into block languages [12,13,14]. In this particular case of application development, we analyse the ease and agility of using block-based versions of the map-reduce constructs from the functional programming paradigm versus the iterative constructs (i.e., loops) from the imperative programming paradigm. The research questions posed for this study are the following: RQ1—Is there any difference in users’ perception of the complexity of the stream processing blocks? RQ2—Is it easier for users to develop apps that collect and process data samples using functional blocks rather than using imperative blocks? and RQ3—Is it faster for users to develop apps that collect and process data samples using functional blocks rather than using imperative blocks?

To find answers to the research questions, the following scenario was carried out. First, all the academics interested in enrolling in the course were arbitrarily allocated in one of the (six) course editions. Each course lasted five hours and the participants were first taught with a short introduction to the educational applications of mobile devices. Next, the instructors explained the fundamentals of visual programming and the VEDILS tool’s features.

Second, to reinforce and consolidate what was learned, participants created a number of educational mobile apps. These apps leverage the smartphone sensory and multimedia elements provided by App Inventor as well as the augmented reality capabilities provided by VEDILS. During the course, all the participants had to develop the same apps, except for one that emulates dice rolling. In addition to simulating the dice, in three of the course editions attendants who represented the control groups had to include an additional routine to calculate the count of odd numbers in a sequence of dice roll samples, whereas in the other three editions, attendants who represented the experimental groups had to program the count of even numbers. For the control groups, the attendants were accordingly taught about the loop statements for data processing, whereas for the experimental groups, the participants were taught about stream blocks.

Finally, the course attendants were asked to develop a citizen science mobile app by themselves. In this vein, smartphones enable to automate data collection and enrich observations with photographs, sound recordings and global positioning system (GPS) coordinates using embedded sensors [49]. The app requirements were: (i) to simulate the input of a numerical measurement of an external phenomenon and (ii) to compute the average of the collected measurements, excluding values out of a permitted value range.

The development of the citizen science app was required to obtain the course completion certificate. The assignment delivery was due within two weeks of course completion. In addition to submitting the developed apps, an online questionnaire had to be filled out. Answers to the questionnaire were analysed using quantitative techniques.

### 5.2. Data Compilation

A total of 45 users attended the VEDILS course. Data collection was performed without interacting with the subjects through an online questionnaire. The survey included questions related to the participants’ knowledge area, age, gender, years of teaching and research experience, highest academic degree obtained and prior expertise in creating computer programs with a visual and/or text-based programming language. Regarding the post-test, questions related to the perceived ease of use of App Inventor and VEDILS were included. These questions pointed to several aspects, such as the use of variables and data lists, control flow statements and loop blocks (for the control groups) and the use of stream blocks (for the experimental groups). In addition, similar questions were included to check the participants’ self-confidence when developing the citizen science app. The answers to all the questions were on a five-level Likert scale.

Besides, all the app project files submitted to the learning management store (i.e., Moodle) for the instructor’s review were subsequently processed through a data integration process for analytic purposes. Among other data, the following were automatically extracted: time spent to develop the app, the number of blocks used, number of debugs and compilations required to complete the app. Study data as well as the resources used are linked in the Supplementary Materials.

### 5.3. Analysis and Findings

The 45 participants (17 women and 28 men) were, on average, 41 years old, had 13 years of teaching experience and 11 years of researching experience. Furthermore, 62.22% of the academics had a Ph.D. Their background is as follows: Arts and Humanities (2.22%), Computer Science (20%), Engineering and Architecture (4.44%), Health Sciences (17.78%), Laws and Social Sciences (28.89%) and Natural Sciences (26.67%). In terms of their previous programming experience, from nothing (1) to expert (5), they had scarce visual (avg = 1.82) and textual (2.15) programming skills. Overall, 25 subjects were part of the control groups, whereas the experimental groups were composed of 20 subjects.

Table 1 and Table 2 show the users’ perception of the stream processing blocks complexity and the ease of development of the app created to collect and process data samples. Data are grouped in the table according to the participants’ gender, academic degree, knowledge area and previous experience with visual and textual programming languages.

Concerning the participants’ gender, women perceived that the stream blocks were easier to use (avg = 4.4) than for men (avg = 3.69) but interestingly enough, men were the ones who found the app development more comfortable with those blocks (man’s avg = 4.08 vs. woman’s avg = 3.33). Furthermore, non-doctorates found the development much easier with stream blocks (avg = 4.25) than the traditional ones (avg = 2.88). Besides, Social Sciences and Humanities (SSH) academics perceived the stream blocks easier to use (avg = 4.75) compared to the Earth & Health Sciences and Engineering (EHSE) lecturers (avg = 3.64). SSH academics also found it difficult (avg = 2.22) to develop the app with the loop blocks, whereas they did not have that much trouble with the stream ones (avg = 3.6).

It is interesting to note (*p* < 0.05) that users with previous experience in visual programming languages perceived loop blocks (avg = 4.57) easier than stream blocks (avg = 3.56). Nevertheless, there is a significant difference (*p* < 0.05) in the fact that academics without experience with visual languages developed the app easier with the map-reduce blocks (avg = 3.71) than with the standard loop blocks (avg = 2.55). As expected, there is also a significant difference (*p* < 0.05) concerning the ease of development of the proposed app with the map-reduce blocks (avg = 3.54) compared to the standard loop blocks (avg = 2.07) for academics without experience with textual languages.

Regarding the apps the lecturers had to create as final assignment of the course, 39 out 45 were correctly developed: 16 apps use the traditional loop blocks and 23 use the stream blocks. Table 3 shows the direct metrics obtained from the app projects. As can be observed, all the apps which had to be developed with stream blocks were completed. The remaining six apps were expected to be developed using the traditional loop blocks. On average, three hours were needed to develop the app with the standard loop blocks, whereas fewer than two hours were required to create the same app with the new stream blocks. That is also tested with a significant difference (*p* < 0.05). Furthermore, the average number of builds and debugs performed for the stream-based apps is fewer than for the loop-based one.

To sum up, with regard to the question (RQ1), there is no difference in the users’ perception of the complexity of the stream processing blocks (avg=3.89) and the loop blocks (avg = 3.72). Concerning whether it is easier for users to develop apps which collect and process data samples with functional blocks rather than with imperative blocks (RQ2), the participants agreed that the development of the requested app was easier with the map-reduce blocks (avg = 3.84) than with loop ones (avg = 2.96). Finally, the indicators obtained for RQ3 point that it is faster for users (100% completion of the projects, a fewer number of debugs required to develop the app and a significant difference (around 38%) in saving development time) to collect and process data samples with functional blocks rather than with imperative blocks.

## 6. Discussion and Conclusions

Developing smart user experiences based on IoT technologies is a very complicated task, especially for non-IT professionals. To address these barriers, some of the popular block-based tools aimed at learners in computer programming (e.g., Scratch or App Inventor) were extended with modules to communicate with external hardware. However, they do not provide adequate support for easily ingesting, processing and visualising data from sensors.

In this research, some components and blocks developed explicitly for a custom version of App Inventor, called VEDILS, were proposed. They are devised to facilitate the ingestion of data from sensors in time intervals, to process received data by using a pipelined sequence of mapping, filtering and reducing operations, and finally, to represent them graphically or in a tabular format.

Two studies, namely a quasi-experimental study conducted with students and an experimental with academics, were conducted to evaluate the contribution. From the first study, students considered that it was easier for them to develop the routines for ingesting, processing and visualising data from the external temperature sensor with the VEDILS IoT features rather than with the equivalent components and blocks in App Inventor. From the second study, aimed at only checking the data processing blocks, participants did not perceive stream blocks easier than the loop blocks. Nevertheless, with statistical significance, it was faster for academics to develop the proposed app with the stream blocks, and easier specifically for novice programmers.

Additionally, threats to validity must be taken into account. To maximise the internal validity and the construct validity, we maintained a detailed protocol for both studies. Peer researchers reviewed them, and actions were considered to minimise bias. In the first study, the students completed the app development projects for both App Inventor and VEDILS but in reverse order to minimise the learning effect on the subjects. In the study conducted with academics, they were randomly distributed into different groups. In addition, every course edition was taught with the same instructors (also the authors of this paper). Furthermore, all course attendants were required to develop the same app with the same requirements to ensure the count of minutes spent, debugs and builds required to create the apps were not affected by other factors. Apart from the data automatically extracted from the developed projects (time spent, number of builds and number of debugs), the rest of the variables used for our experiments to measure user perceptions are subjective so that they can also be considered as validity threats.

The limited size of the student sample can be viewed as an external validity threat. Moreover, although the second study has a user sample more extensive than the first one, it is only aimed at academics. As a result, we cannot assure that the obtained findings can be generalised to professionals of other disciplines or conventional users. Hence, more experimentation and analysis are required to evaluate to what extent the findings presented in this work are of relevance for other cases.

It is necessary to consider the limitations of the current work. First, since the new type of language block for providing app developers with a data stream according to a predefined period of time was only incorporated for the standard *BluetoothClient* component and the *BrainwaveSensor* of VEDILS, it is not currently possible to harness it for other built-in App Inventor sensors. In addition, the extension component for using Bluetooth Low Energy (BLE) technology, which is not part of the App Inventor main distribution, is not yet supported for our contribution. Second, the current implementation of the components for visualising data do not allow developers to customise colours, lines widths, font sizes, etc., which are format aspects usually required when designing charts.

Smart homes and buildings, smart cities, mobility and transportation, healthcare, agriculture and industry are some of the main areas of IoT application [1]. The study conducted with students illustrated the potential application of our approach to smart buildings, e.g., for monitoring room temperature. Nevertheless, the contribution presented in the paper is expected to be useful to create IoT applications for other areas. Thus, for example, non-expert programmers (researchers, patients and healthcare professionals) will be able to develop apps for wellness and healthcare purposes without struggling with the complexities of the common mobile programming languages, namely Java or Swift. These kinds of apps are usually data-intensive and require to process users’ biometric data, which is ingested from wearable devices, such as smart bands or chest straps, among others.

This research tried to investigate whether the components and extensions presented in the paper contribute to the popularisation of IoT-based mobile app development. In this vein, EUD platforms and, in particular, enriched block-based authoring tools as VEDILS, can simplify development tasks of novice end-user programmers. Furthermore, according to the obtained results, the use of blocks based on the map-reduce paradigm from functional programming streamlines the development of data processing functions in IoT consumer apps, although more experimentation is required. As future work, we plan to support the BLE extension for App Inventor to improve the customising features of the *Chart* and *DataTable* components.

## Figures and Tables

**Table 1:** Results of the survey with academics: perceived ease of use (the italic font shows the average and chi-squared values whereas the bold one indicates significant differences).

User Profile	Loop Blocks	Stream Blocks	*Average*	*Chi-Squared*
*Gender*				
Man	3.71	3.69	*3.71*	*0.72*
Woman	3.73	4.40	*3.94*	*0.55*
*Chi-squared*	*0.61*	*0.41*		
*Academic degree*				
Non-doctorate	3.77	4.00	*3.88*	*0.09*
Doctorate	3.68	3.82	*3.75*	*0.24*
*Chi-squared*	*0.46*	***0.04***		
*Knowledge area*				
EHSE	4.00	3.64	*4.00*	*0.48*
SSH	3.22	4.75	*3.22*	*0.29*
*Chi-squared*	*0.46*	*0.17*		
*Experience with visual programming languages*				
Non-experienced	3.89	4.00	*3.67*	*0.31*
Experienced	4.57	3.60	*4.17*	***0.02***
*Chi-squared*	*0.19*	***0.01***		
*Experience with textual programming languages*				
Non-experienced	3.21	3.83	*3.54*	*0.58*
Experienced	4.36	4.00	*4.24*	*0.46*
*Chi-squared*	*0.17*	*0.32*		
*All academics*				
Academics	*3.72*	*3.89*	*3.80*	*0.83*

**Table 2:** Results of the survey with academics: ease of development of the app (the italic font shows the average and chi-squared values whereas the bold one indicates significant differences).

User Profile	Loop-Based	Stream-Based	*Average*	*Chi-Squared*
*Gender*				
Man	3.07	4.08	*3.57*	*0.17*
Woman	2.81	3.33	*3.00*	*0.37*
*Chi-squared*	*0.48*	***0.05***		
*Academic degree*				
Non-doctorate	2.88	4.25	*3.53*	*0.31*
Doctorate	3.00	3.54	*3.25*	*0.60*
*Chi-squared*	*0.23*	*0.32*		
*Knowledge area*				
EHSE	3.37	3.93	*3.65*	*0.63*
SSH	2.22	3.60	*2.71*	*0.18*
*Chi-squared*	*0.23*	*0.29*		
*Experience with visual programming languages*				
Non-experienced	2.55	3.71	*3.09*	***0.05***
Experienced	4.00	4.20	*4.08*	*0.48*
*Chi-squared*	*0.06*	*0.52*		
*Experience with textual programming languages*				
Non-experienced	2.07	3.54	*2.82*	***0.01***
Experienced	4.09	4.50	*4.24*	*0.54*
*Chi-squared*	***0.00***	*0.11*		
*All academics*				
Academics	*2.96*	*3.84*	*3.36*	*0.13*

**Table 3:** Indicators of the developed apps (the italic font shows the average and Mann-Whitney U Test values whereas the bold one indicates significant differences).

		% Completion	Minutes Spent	Number of Debugs + Builds
Loop-based		72%	180.71	13.25
Stream-based		100%	111.97	9.74
	*Average*	*86.66%*	*140.17*	*11.18*
	*Mann-Whitney U Test*		***0.024***	*0.16*

## Supplementary Materials

The software for authoring IoT apps with data processing and visualising capabilities can be used through the VEDILS web site http://vedils.uca.es/. Besides, to guarantee the reproducibility of the studies, all the resources developed are available online at https://www.mdpi.com/1424-8220/19/24/5467/s1. This link provides a ZIP package containing: questionnaires and results of both studies, PDF presentations (in Spanish) for the course with academics and the workshop with students and solutions for the different exercises and tutorials.

## Abbreviations

The following abbreviations are used in this manuscript:

BLE	Bluetooth Low Energy
EEG	electroencephalography
EHSE	Earth & Health Sciences and Engineering
EUD	end-user development
EUP	end-user programming
FFT	fast Fourier transform
GPS	global positioning system
HMI	Human Machine Interface
IoT	Internet of Things
MIT	Massachusetts Institute of Technology
PL	pocket lab
SSH	Social Sciences and Humanities
TAP	trigger-action programming
VEDILS	Visual Environment for Designing Interactive Learning Scenarios
YAIL	Young Android Intermediate Language
