# Supplementary material for: Block-Based Development of Mobile Learning Experiences for the Internet of Things
Source: Sensors (Basel). 2019 Dec 11;19(24):5467. doi: 10.3390/s19245467 (PMC6960931; doi:10.3390/s19245467)
Supplement: Supplementary file 1 [file sensors-19-05467-s001.zip › Study with academics/Slides (in spanish).pdf]

Desarrollo fácil y rápido de apps para dispositivos Android (Nivel **inicial**)

Curso de innovación docente. 2018

José Miguel Mota Macías josemiguel.mota@uca.es
Iván Ruiz Rube ivan.ruiz@uca.es

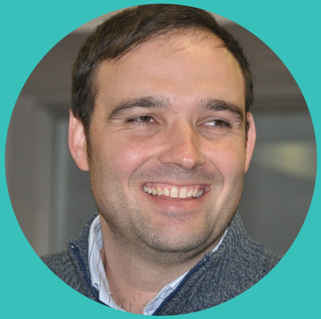

Hola!

IVÁN RUIZ RUBE

Profesor del *Dpto. de Ingeniería Informática*
Investigador en *Software Process Improvement, Linked Open Data* y *Technology-Enhanced Learning*

@iruizrube

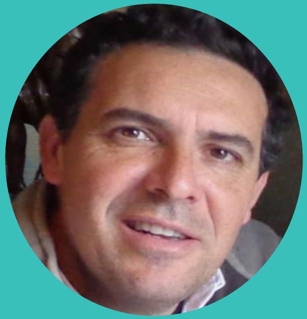

Hola!

JOSÉ MIGUEL MOTA MACÍAS

Profesor del *Dpto. de Ingeniería Informática*
Investigador en *Technology-Enhanced Learning*, mobile learning, realidad aumentada, real y mixta

@jmiguelmota

Material del curso

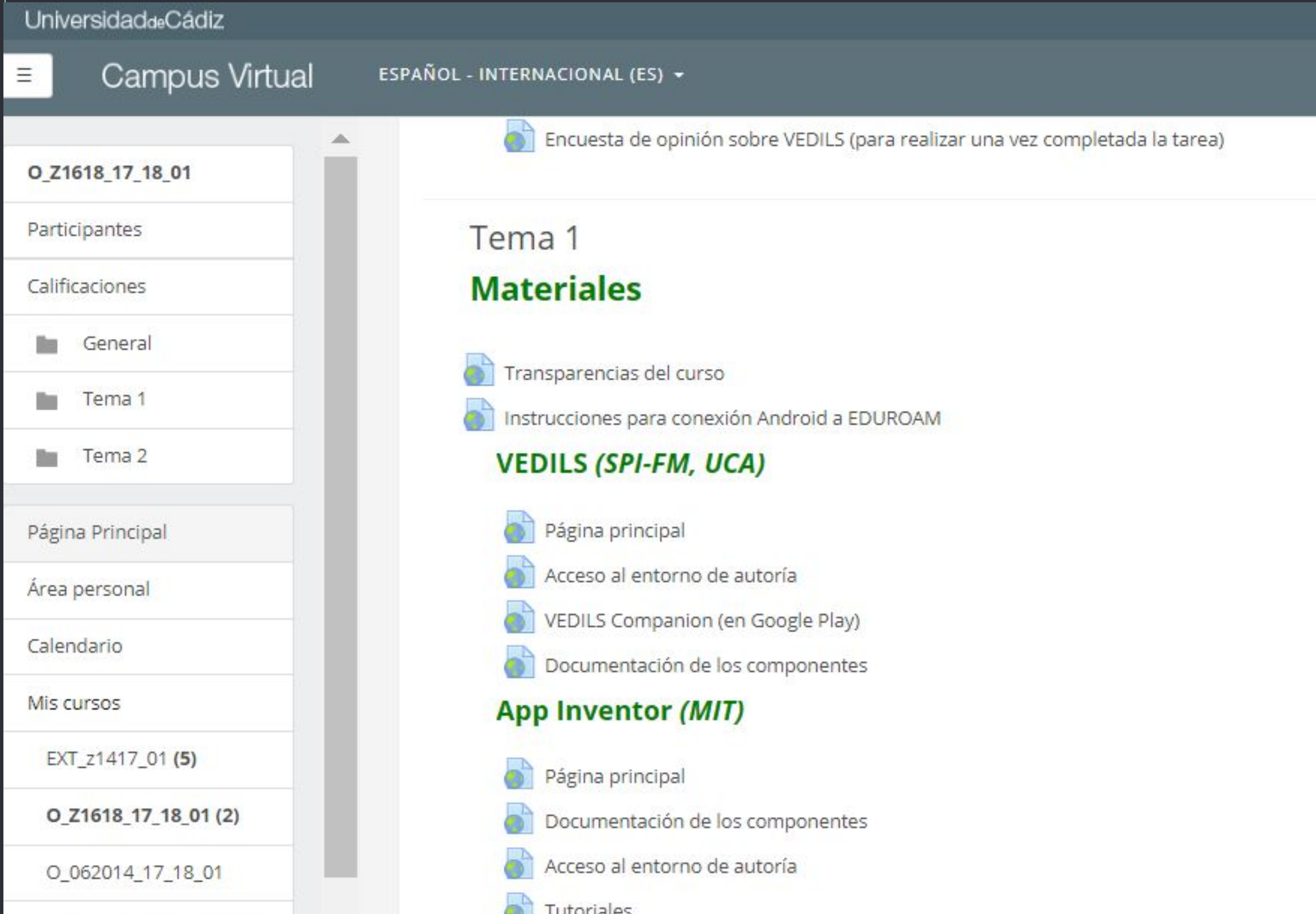

Índice

- Introducción
- Experiencias realizadas
- Autoría de aplicaciones
- Ejercicios
- Consejos y conclusiones

1

Introducción

Breve introducción a las nuevas tecnologías aplicables a la docencia

Dispositivos móviles

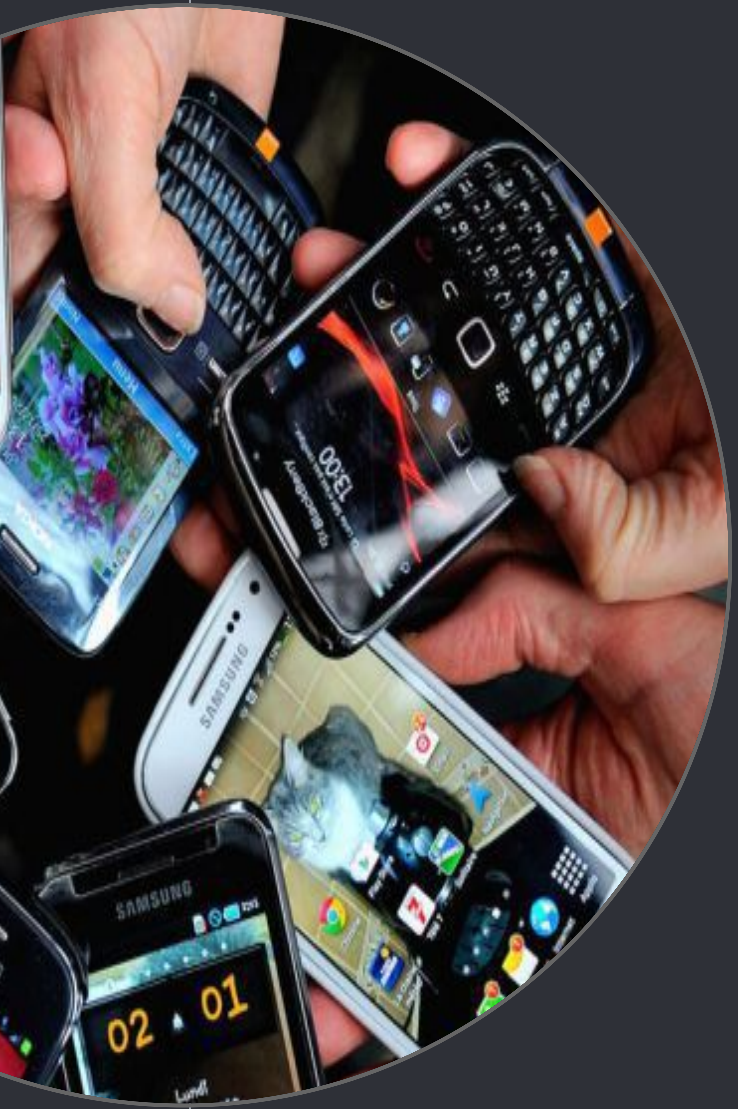

Los dispositivos móviles superarán al número de ordenadores en los próximos años

Comparativa con ordenadores

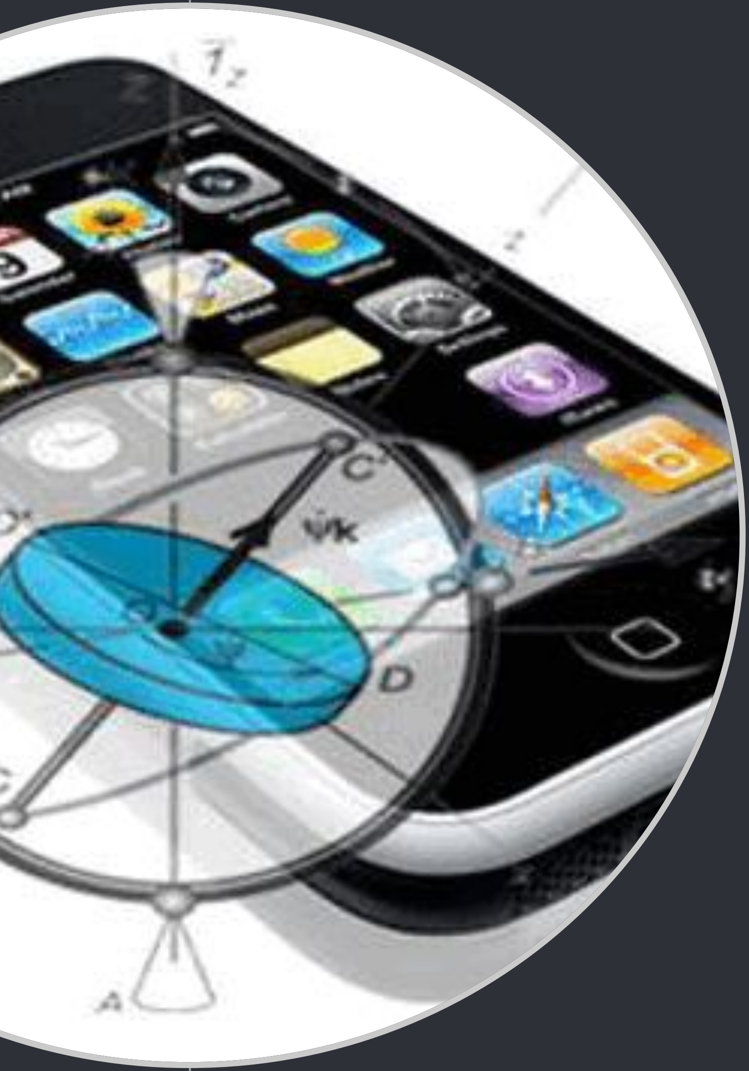

Los smartphones ganan en movilidad y conectividad. Además disponen de sensores: acelerómetro, barómetro, lector de huellas, magnetómetro, podómetro, pulsómetro, sensor de luz, sensor de proximidad, ….

https://medium.com/adventures-in-consumer-technology/smartphones-will-replace-pcs-soon-541b5c8a4f48

Soportan nuevas tecnologías

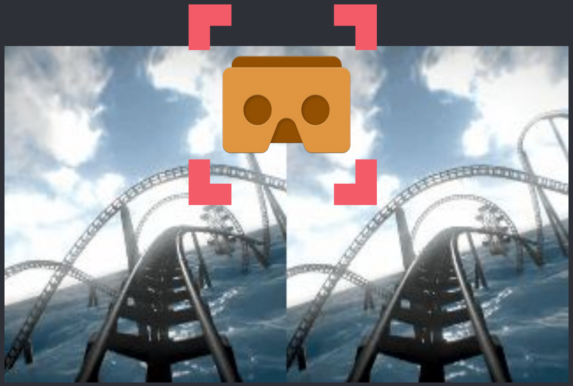

Virtual Reality

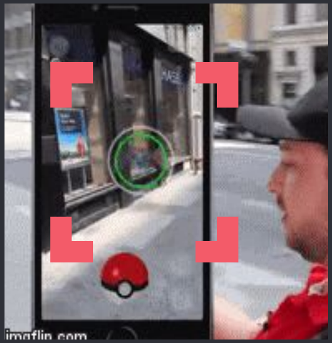

Augmented reality

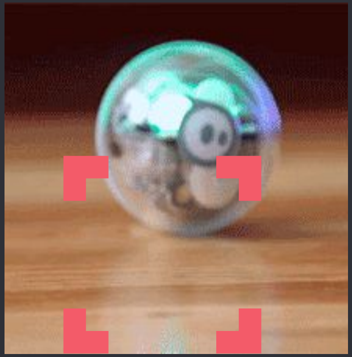

Robot

Sphero

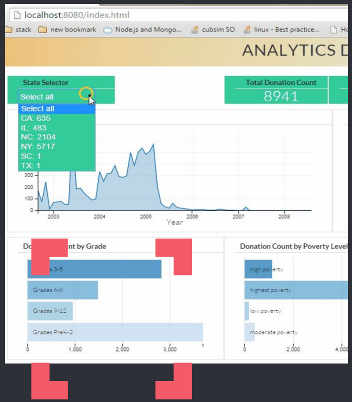

Learning analytics

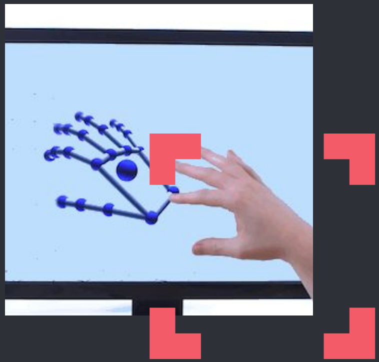

Leap Motion

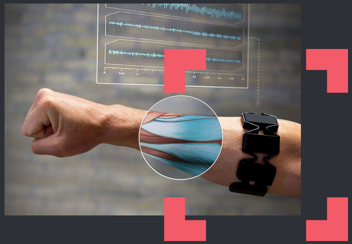

Myo armband

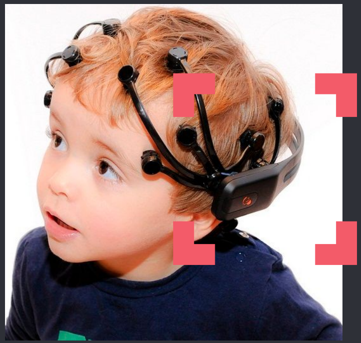

Emotiv Epoc+

Interacción multimodal

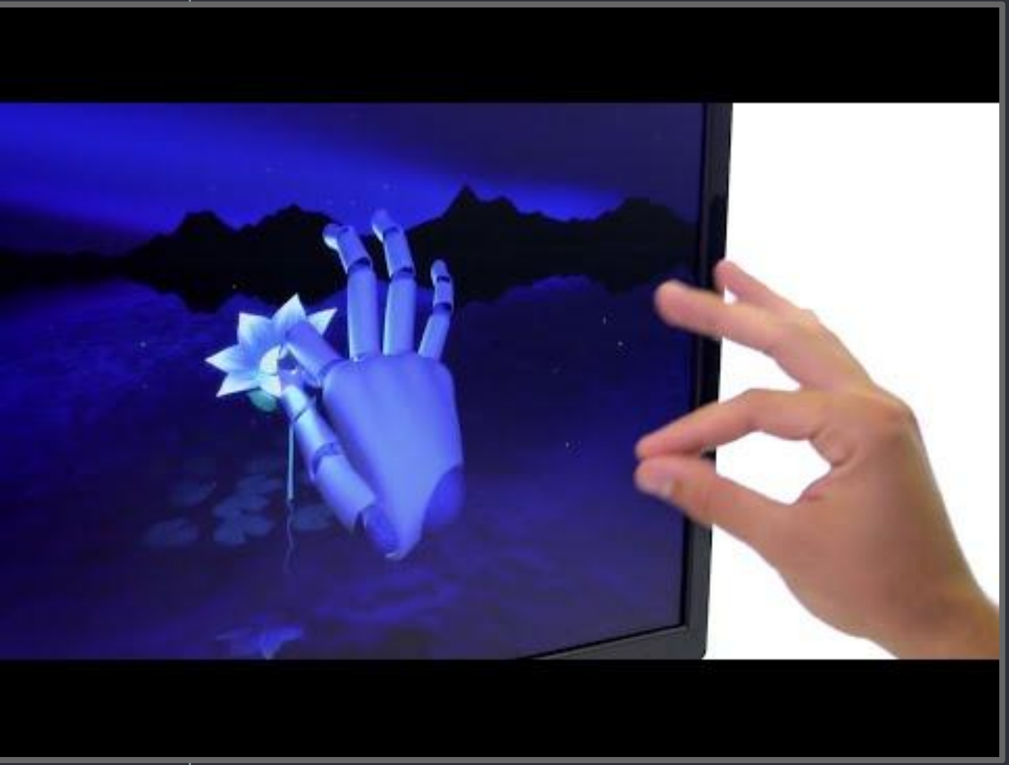

Nuevas maneras de interactuar con los dispositivos físicos

Robótica

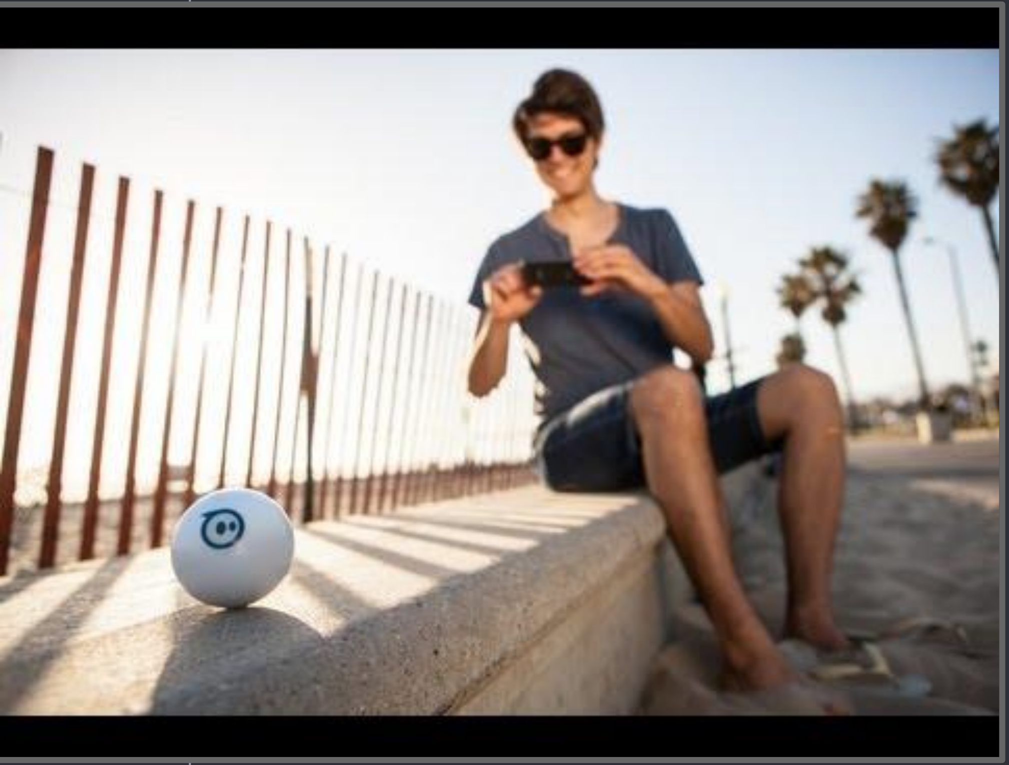

Nuevos dispositivos con los que interactuar:

- **Sphero**
- **Raspberry PI**
- **Arduino**
- ....

Realidad Aumentada

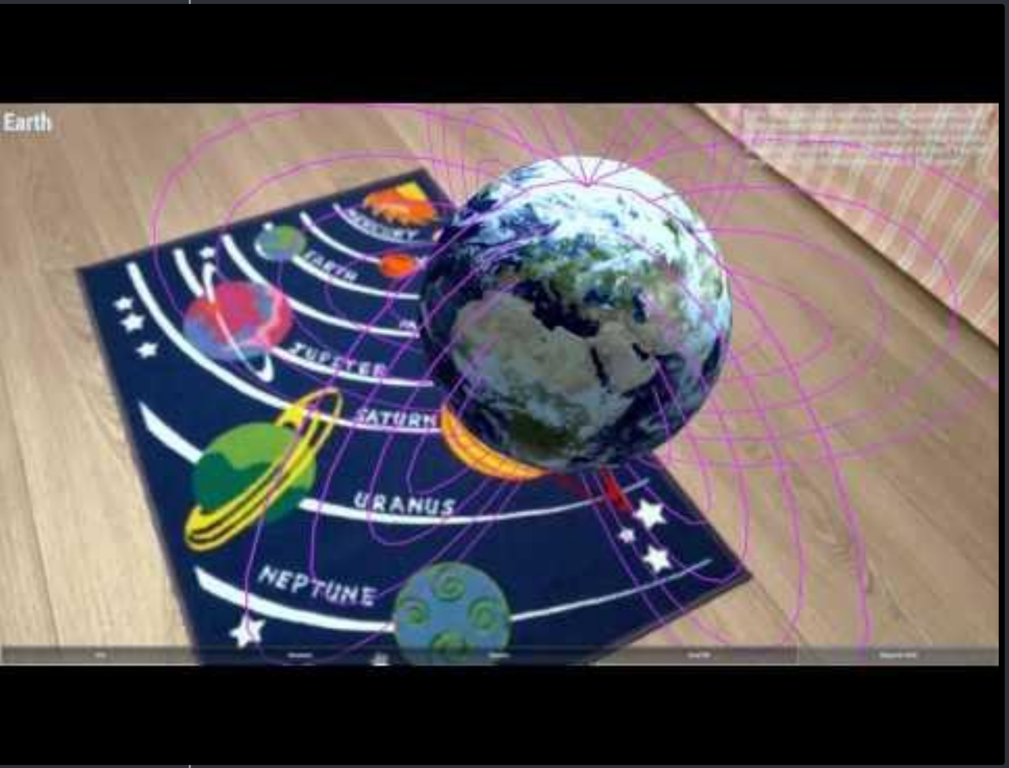

Elementos virtuales son **superpuestos** sobre en el mundo real

Realidad Virtual

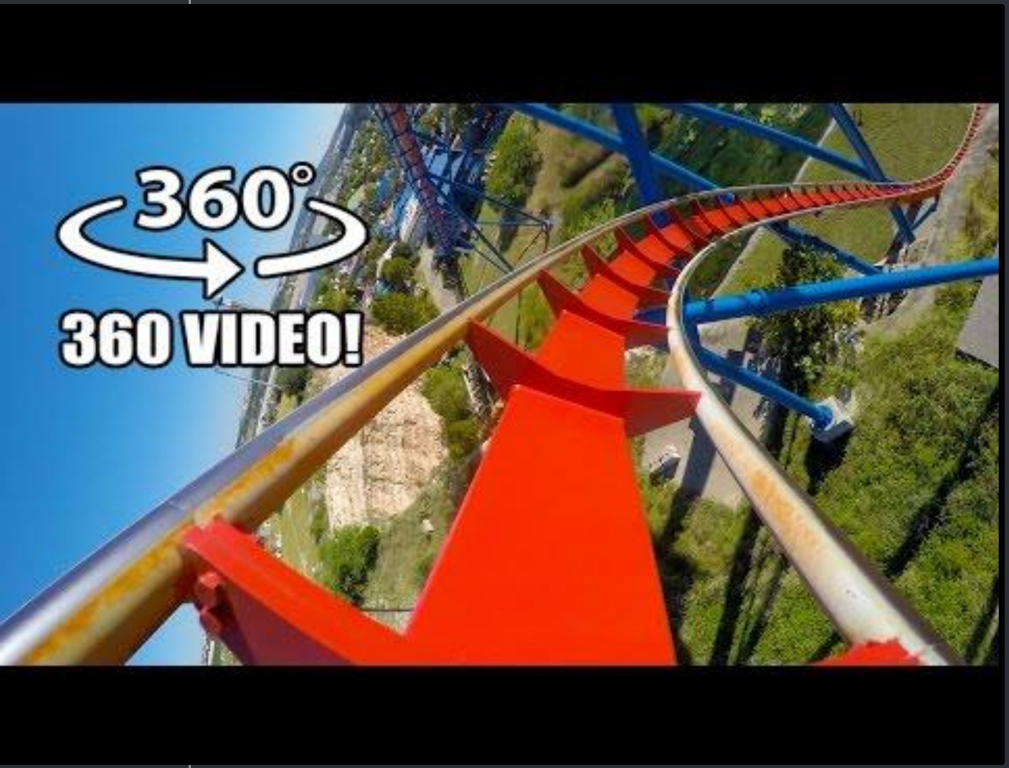

Se **sumerge al usuario en un mundo virtual** con imágenes 3D o vídeos de 360°

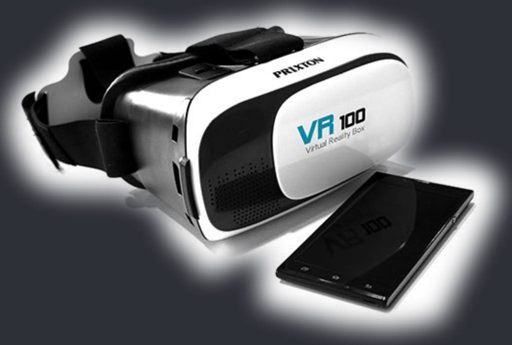

Análisis de datos

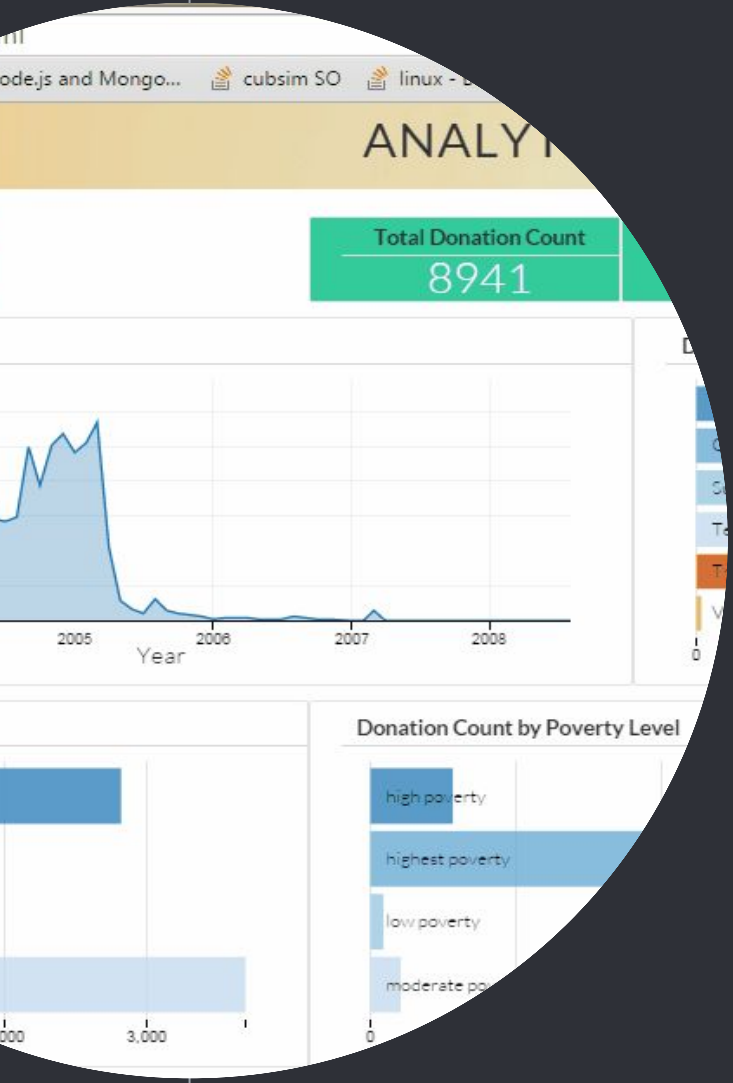

Las interacciones con aplicaciones y dispositivos pueden **recogerse, analizarse, y revertir en modificaciones** en las aplicaciones

Y lo más importante

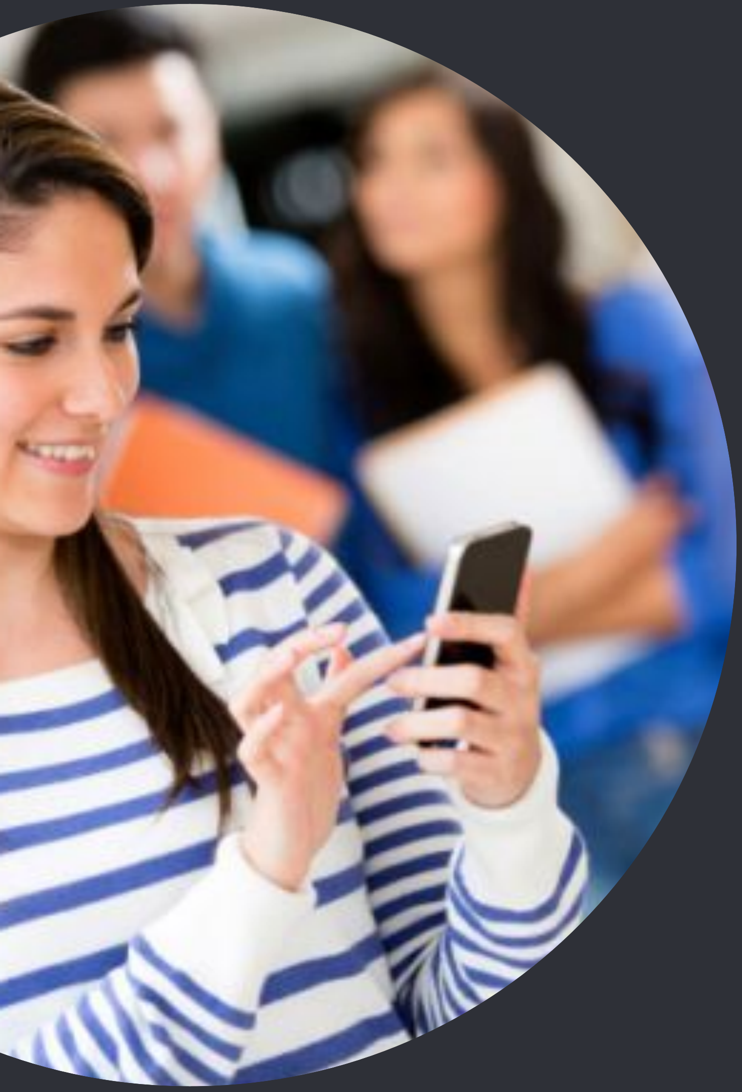

Los alumnos cuentan ahora con dispositivos móviles.

https://www.pinterest.es/pin/309974386828491348/

Nueva modalidad de enseñanza

Aparecen nuevos conceptos aplicados al aprendizaje basado en dispositivos móviles.

- **mLearning** Mobile learning
- **BYOD** Bring your own device
- **PLE** Personal Learning Environment
- **PLN** Personal Learning Network

https://www.pinterest.es/pin/309974386828491348/

¿Se utilizan estas nuevas tecnologías en clase?

Las tecnologías son usadas de una manera **tradicional en clase**

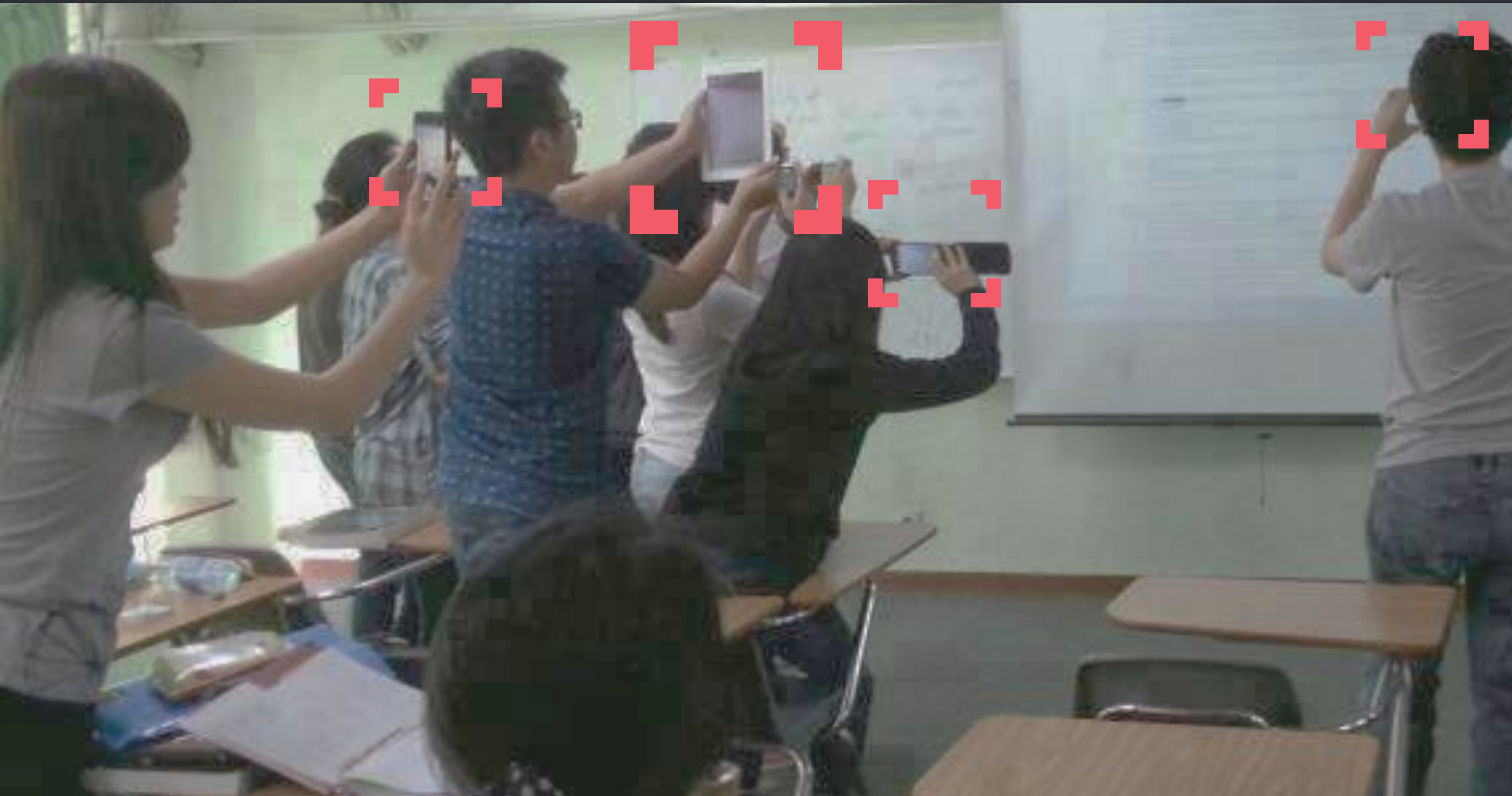

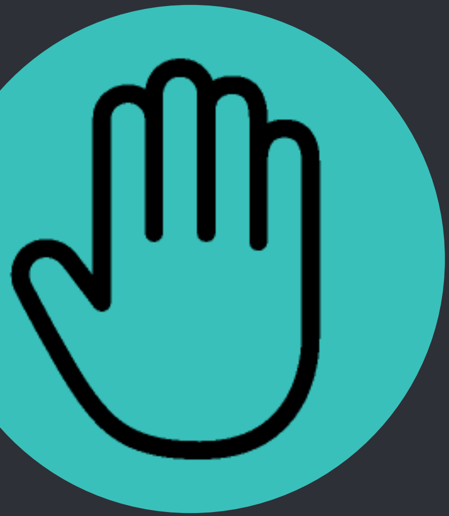

Personalización

Muchos de estos dispositivos vienen con aplicaciones que nos permiten utilizarlos, pero estamos **restringidos** a las posibilidades sus programas nos permiten.

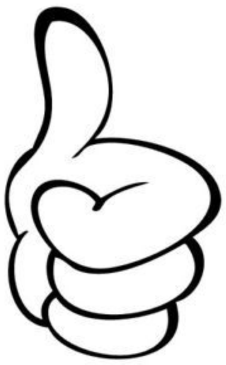

Aprendiendo a programar

La programación nos permite poner la **tecnología a nuestro servicio**, para así formar a productores de contenidos digitales, no solo consumidores.

Programas para introducir a los jóvenes en la programación

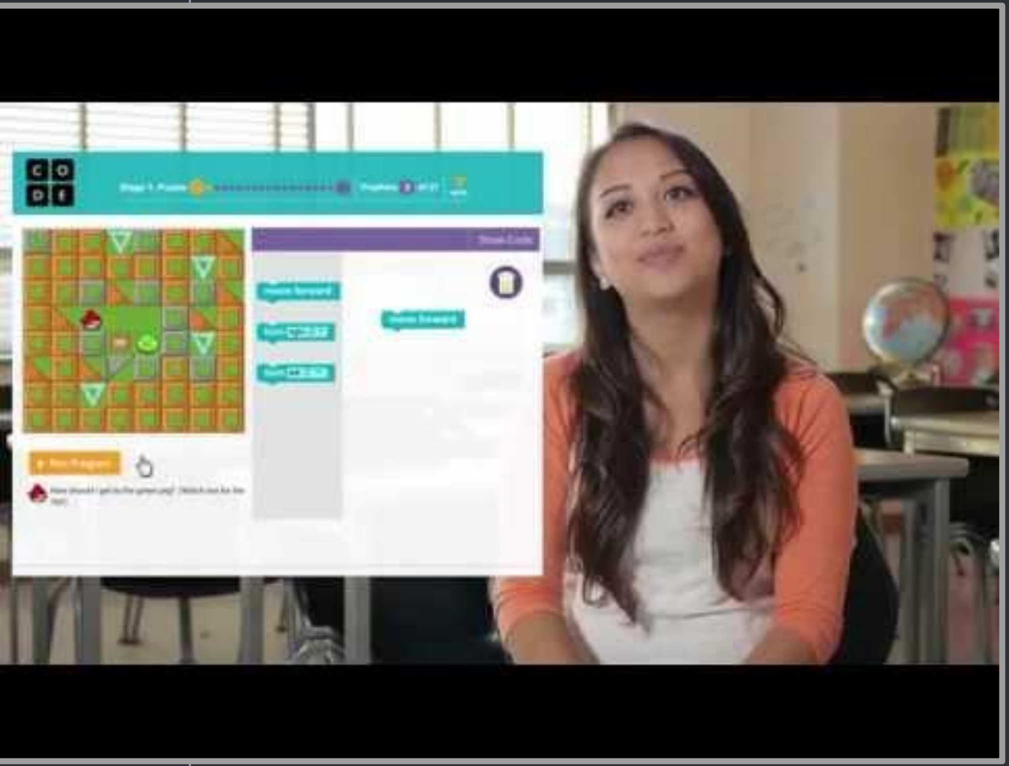

Utilizan temáticas especiales para hacer las aplicaciones más atrayentes

Clases aplicadas UCA

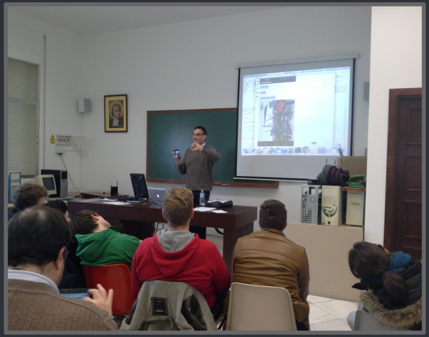

Clases aplicadas en centros de enseñanzas medias desde 2015

- Cádiz, Barbate, Chiclana de la Frontera, Puerto de Santa María, Jerez de la Frontera, San Fernando

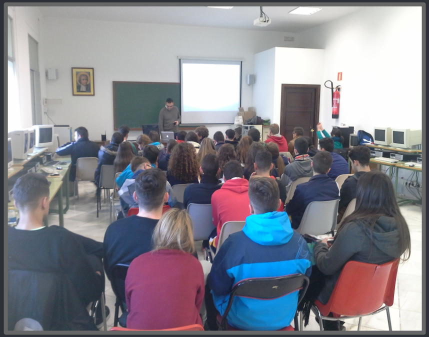

Perfil del alumnado

- Alumnos de Bachillerato
- Ciclos formativos
- Alumnos de 4º de ESO

Cursos de innovación docente UCA

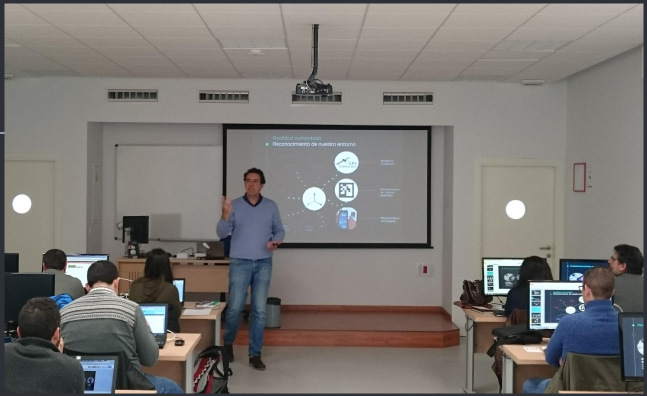

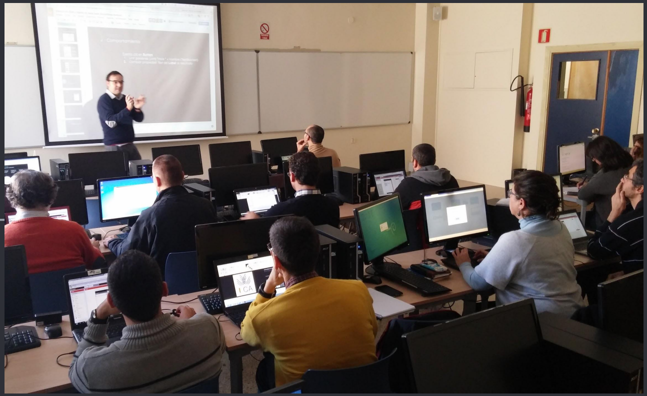

Desarrollo fácil y rápido de apps para dispositivos móviles Android (2017).

- Campus de Puerto Real y Algeciras

Perfil del alumnado

- + 50 docentes e investigadores de la Universidad

Programación para todo el mundo

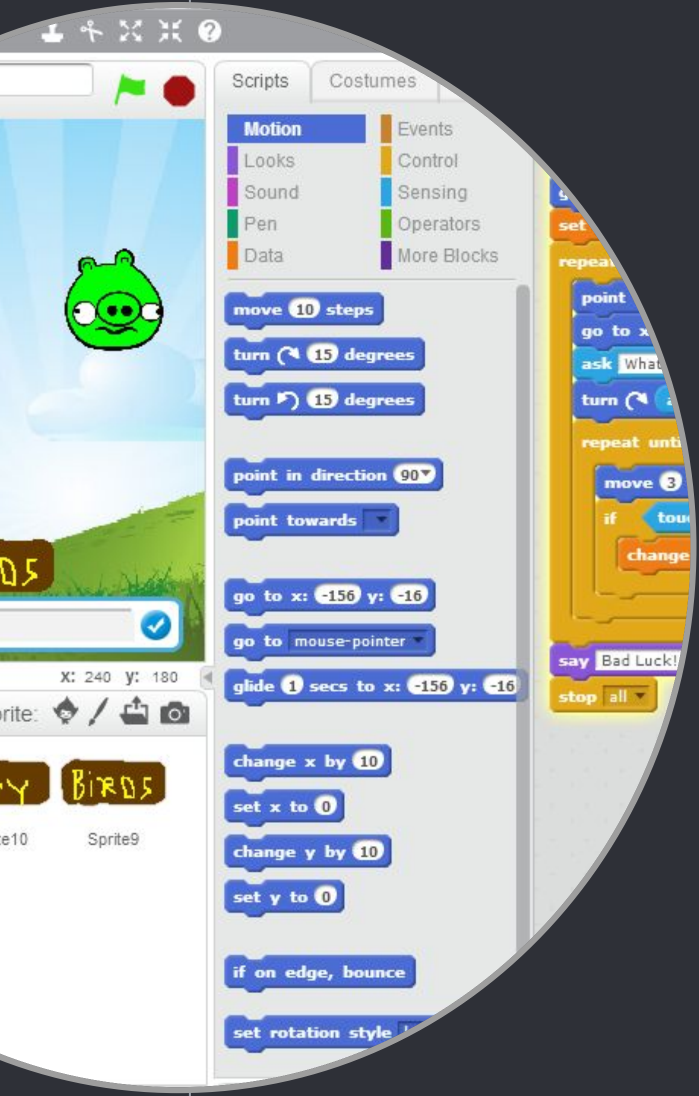

Han aparecido nuevos entornos para el desarrollo de aplicaciones haciendo uso de **lenguajes de programación visuales**

Objetivo

El **objetivo principal** es involucrar a los expertos en la **creación de sus propios escenarios educativos** con los recientes **avances tecnológicos** para conseguir una mejor inmersión de los estudiantes en las actividades de aprendizaje.

Hipótesis

Los **lenguajes de programación visuales** pueden ayudar a la construcción de escenarios educativos y hacer uso de estas nuevas tecnologías

2

Experiencias realizadas

Estas nuevas tecnologías requieren conceptos de programación si se quiere desarrollar aplicaciones propias

¿Todo esto para qué?

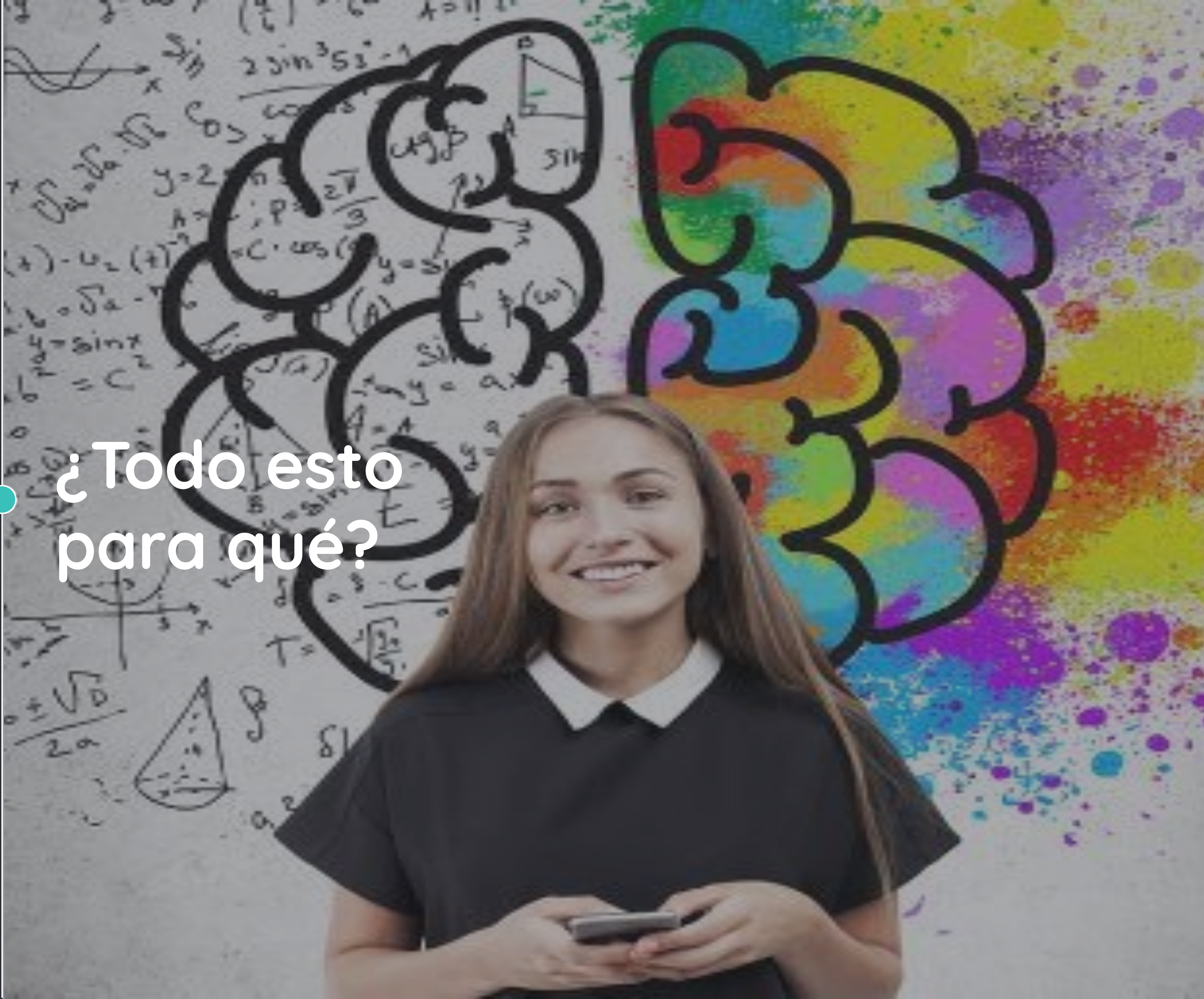

DIBUJO TÉCNICO

En la ***Universidade do Algarve*** en Portugal, los alumnos de Ingeniería Mecánica aprenden en la asignatura *Design 1* los conceptos del dibujo técnico incluyendo las perspectivas

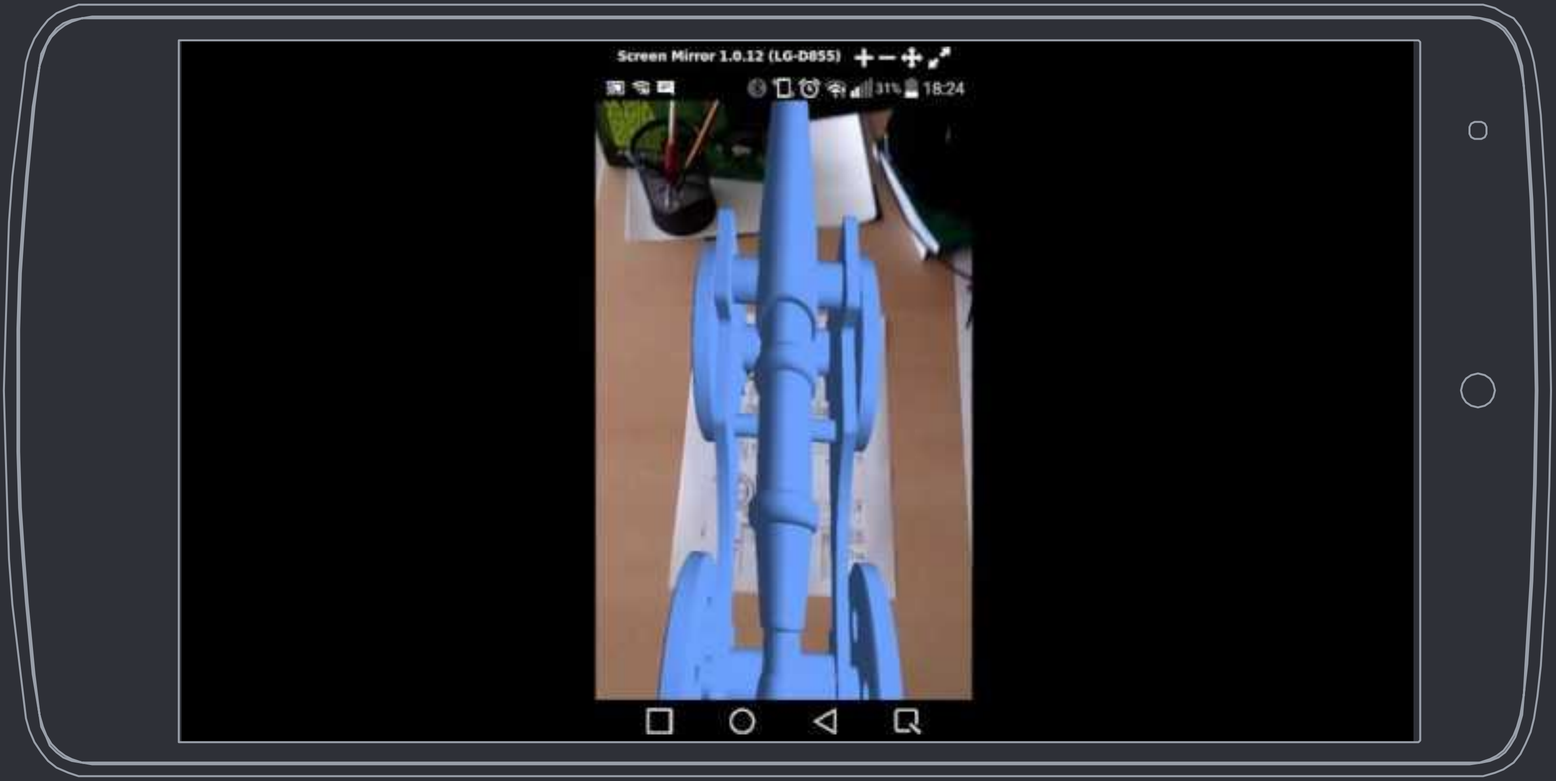

EXPRESIÓN GRÁFICA

En la asignatura Expresión Gráfica y Diseño Asistido del Grado de Ingeniería, los alumnos aprenden dentro de los sistemas de representación el sistema diédrico.

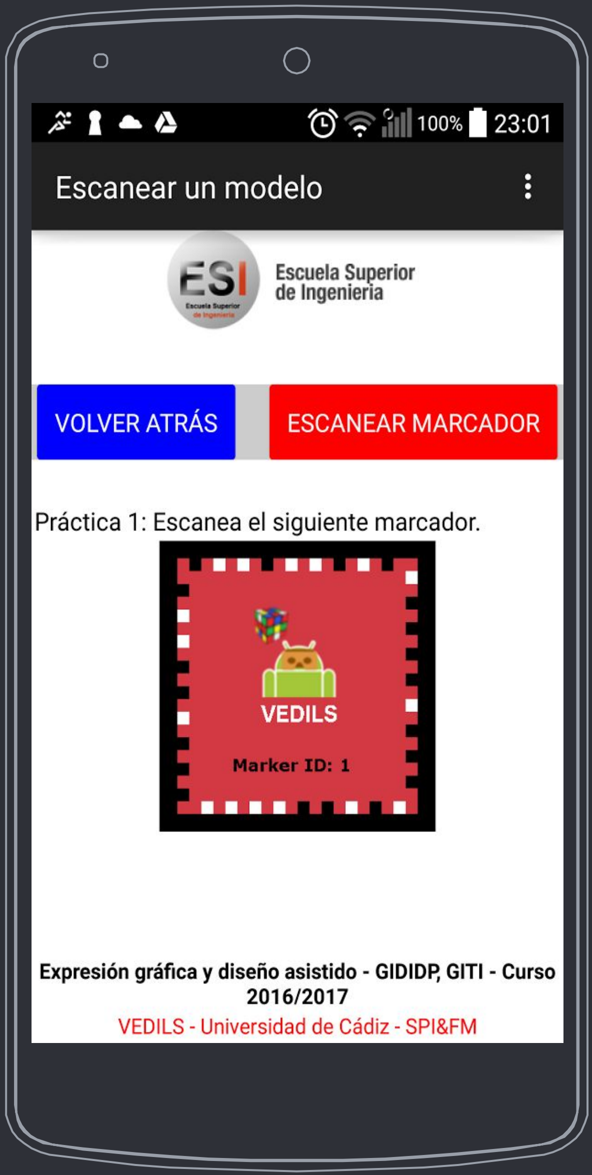

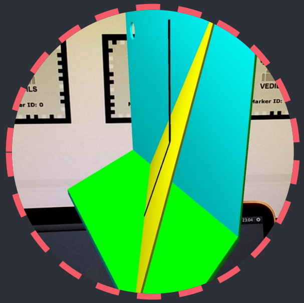

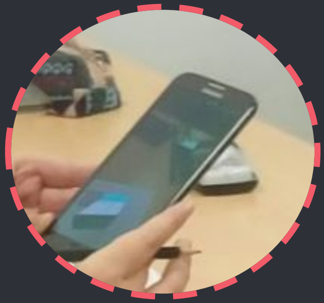

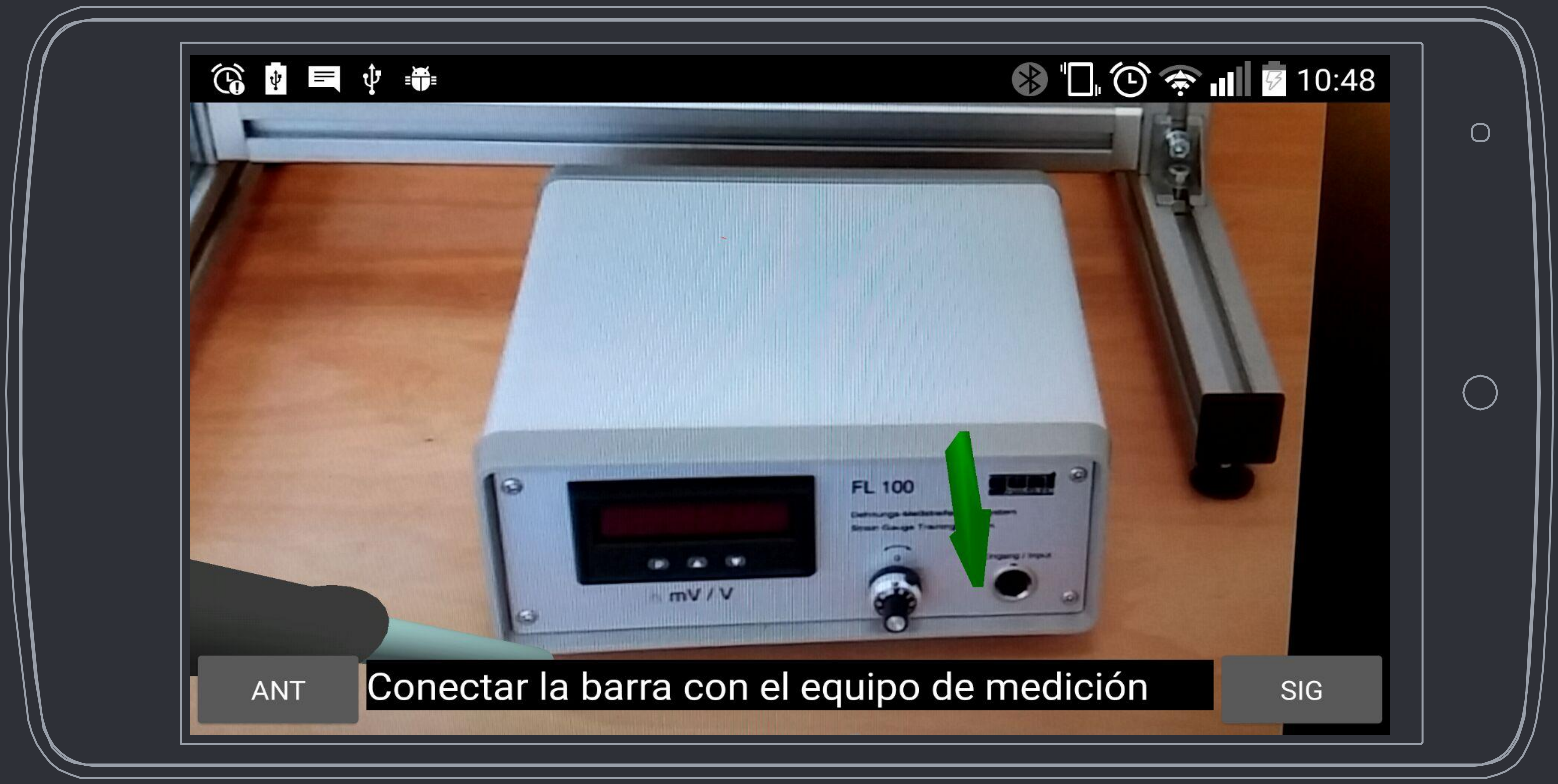

MANUALES INTERACTIVOS

En la asignatura Resistencia de Materiales del Grado de Ingeniería en Diseño Industrial y Desarrollo del Producto en la Universidad de Cádiz, los alumnos aprenden a utilizar las herramientas/máquinas utilizando un manual con Realidad Aumentada

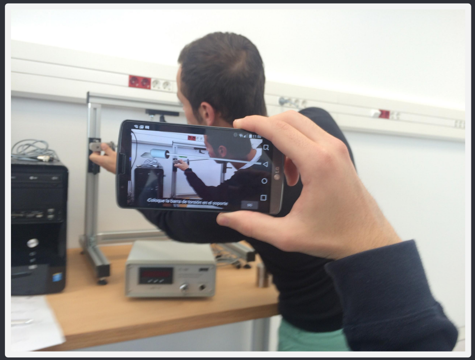

APRENDIZAJE LENGUA EXTRANJERA

Para las prácticas de la asignatura “Alemán 1” se implementó el juego “¿Quien soy yo?” con realidad aumentada, para que los alumnos practicaran la descripción de personajes con sus compañeros

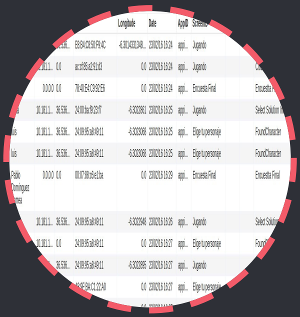

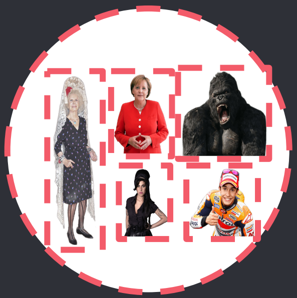

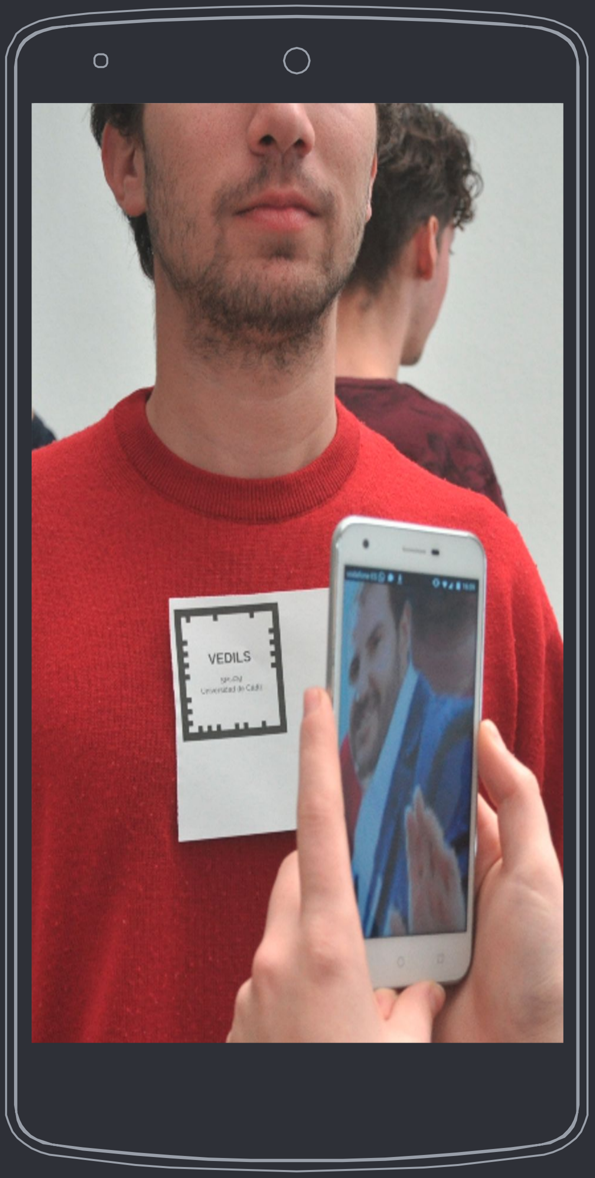

TELÉFONO CHINO

Uso del sistema de reconocimiento y sintetización de voz para “susurrar mensajes”

Se usa durante el aprendizaje de alemán como lengua extranjera en la Facultad de Filosofía y Letras

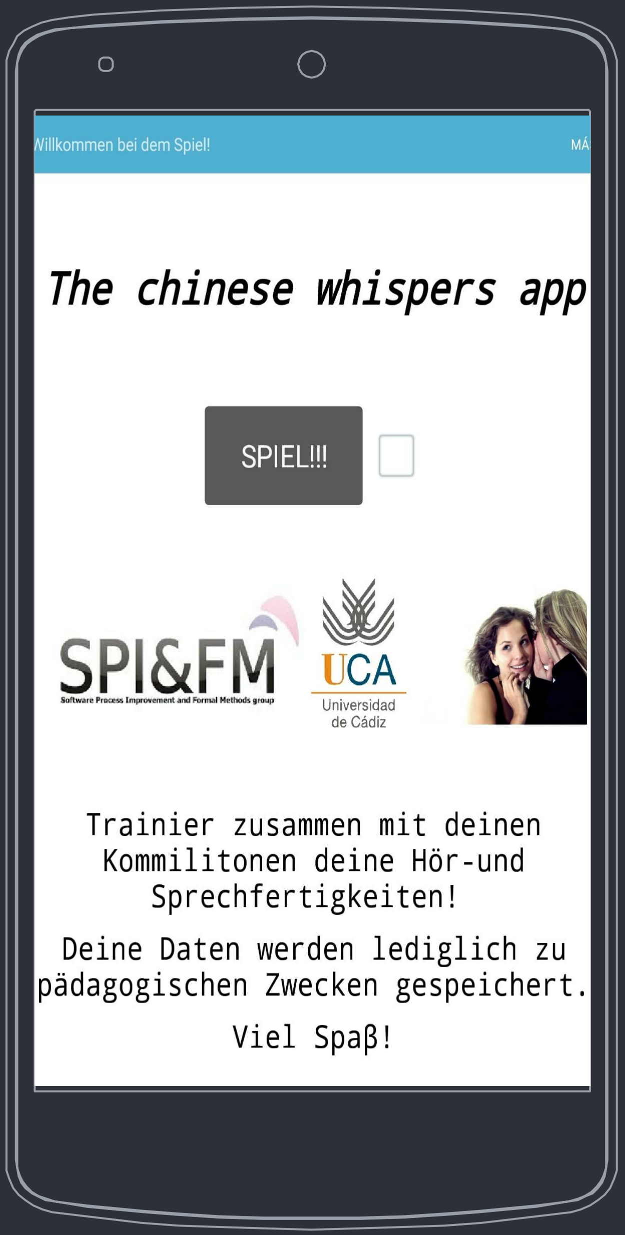

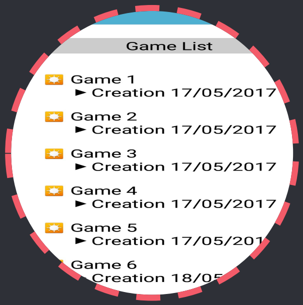

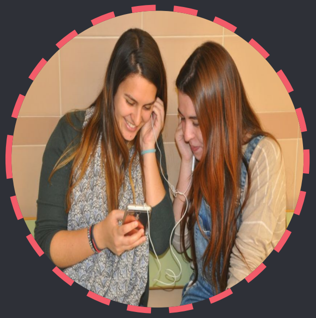

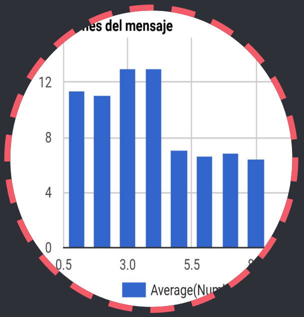

Google Play

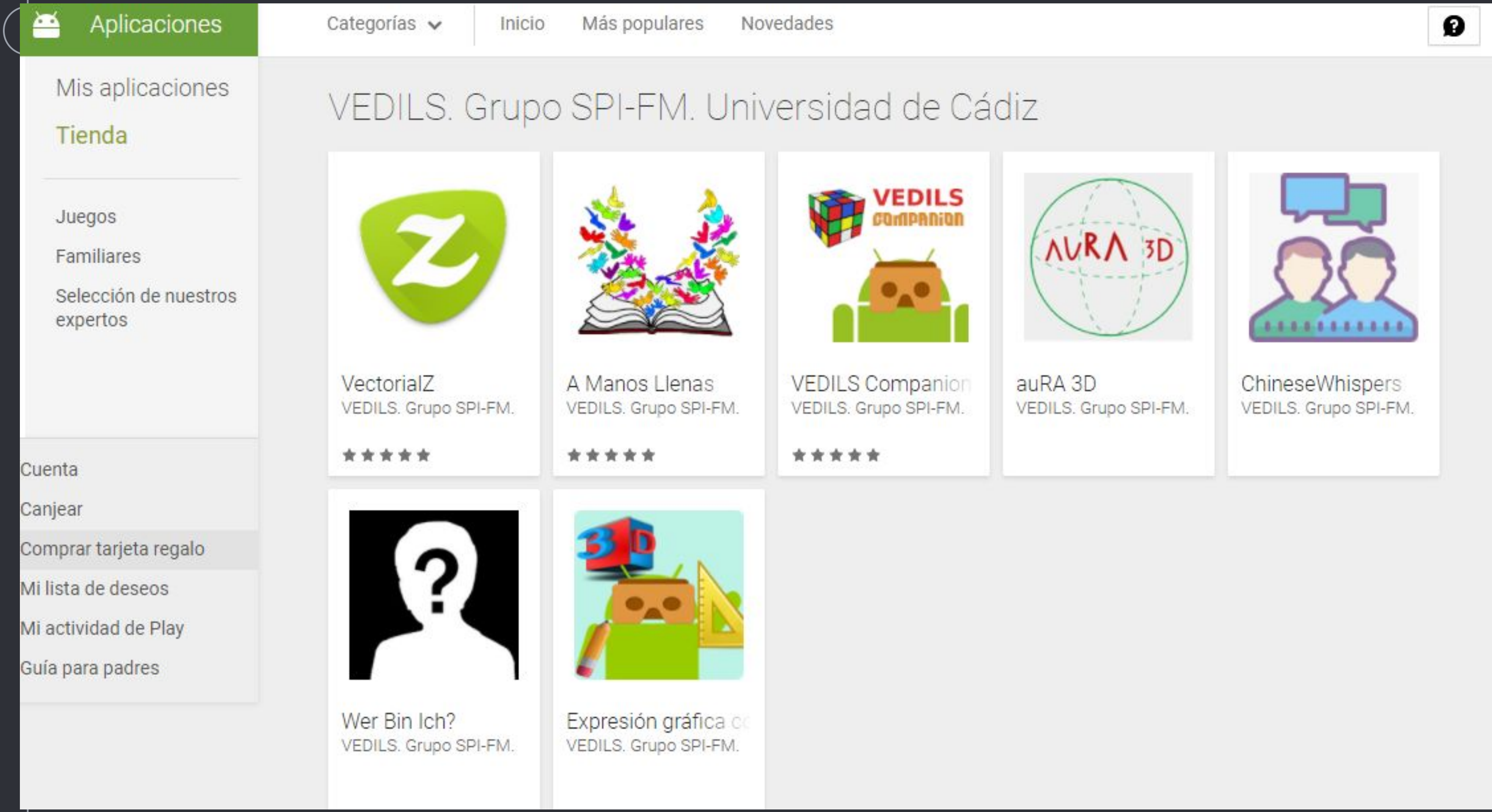

Aplicaciones
Categorías
Inicio
Más populares
Novedades
Mis aplicaciones
Tienda
Juegos
Familiares
Selección de nuestros expertos
Cuenta
Canjear
Comprar tarjeta regalo
Mi lista de deseos
Mi actividad de Play
Guía para padres
VEDILS. Grupo SPI-FM. Universidad de Cádiz
VectorialZ
VEDILS. Grupo SPI-FM.
A Manos Llenas
VEDILS. Grupo SPI-FM.
VEDILS
COMPANION
VEDILS Companion
VEDILS. Grupo SPI-FM.
AURA 3D
auRA 3D
VEDILS. Grupo SPI-FM.
ChineseWhispers
VEDILS. Grupo SPI-FM.
Wer Bin Ich?
VEDILS. Grupo SPI-FM.
3D
Expresión gráfica
VEDILS. Grupo SPI-FM.

3

Autoría de aplicaciones

Vamos a ver cómo podemos realizar nuestras aplicaciones

“

Desarrollo de apps en Android

Android es el sistema operativo para dispositivos móviles de Google basado en Linux.

Las aplicaciones se construyen con el lenguaje **Java**, pero nosotros lo desarrollaremos usando un **lenguaje visual basado en bloques.**

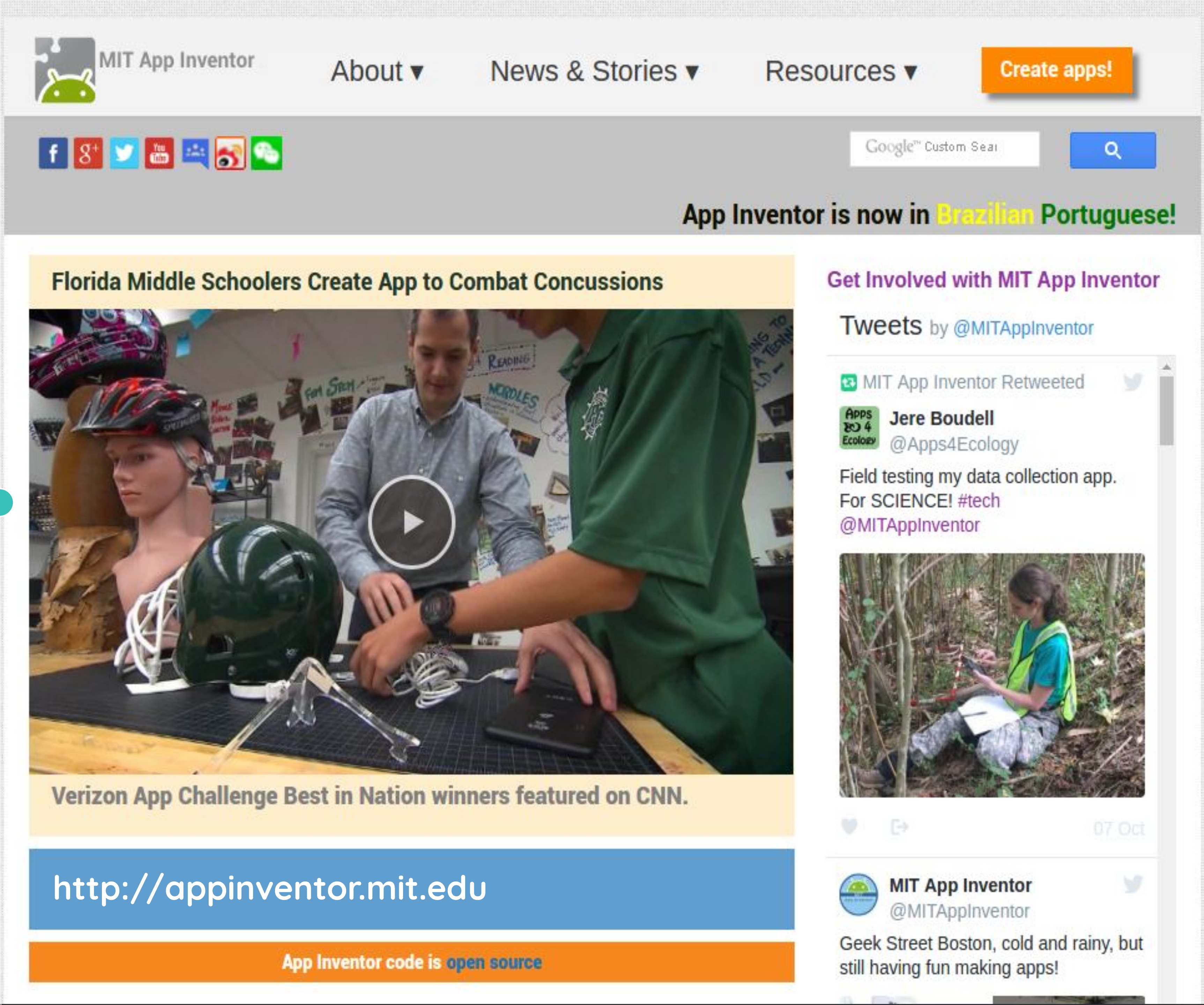
MIT App Inventor
About ▾
News & Stories ▾
Resources ▾
Create apps!
App Inventor is now in Brazilian Portuguese!
Florida Middle Schoolers Create App to Combat Concussions
Verizon App Challenge Best in Nation winners featured on CNN.
http://appinventor.mit.edu
App Inventor code is open source
Get Involved with MIT App Inventor
Tweets by @MITAppInventor
MIT App Inventor Retweeted
Jere Boudell
@Apps4Ecology
Field testing my data collection app. For SCIENCE! #tech @MITAppInventor
07 Oct
MIT App Inventor
@MITAppInventor
Geek Street Boston, cold and rainy, but still having fun making apps!

Flujo de trabajo

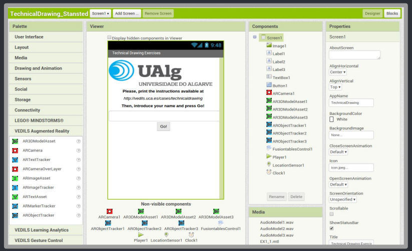

Diseño de la interfaz de usuario

En un **navegador WEB** diseñamos y configuramos los elementos del interfaz de usuario.

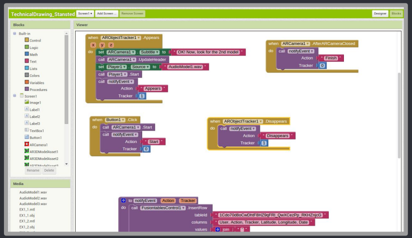

Diseño del comportamiento

Usando un **lenguaje visual de programación** definimos la lógica de la aplicación.

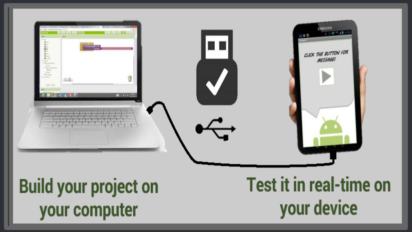

Pruebas de la aplicación

Podemos realizar pruebas en **tiempo real** de nuestra aplicación, para comprobar su comportamiento.

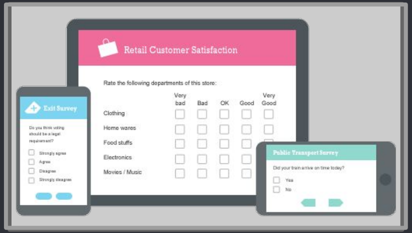

Despliegue en un dispositivo

Generamos un fichero **APK**, que son las aplicaciones que se instalan en el dispositivo Android.

Flujo de trabajo
Diseño de la interfaz de usuario

Paleta de componentes

Editor de interfaz de usuario

Mis componentes

Propiedades de los componentes

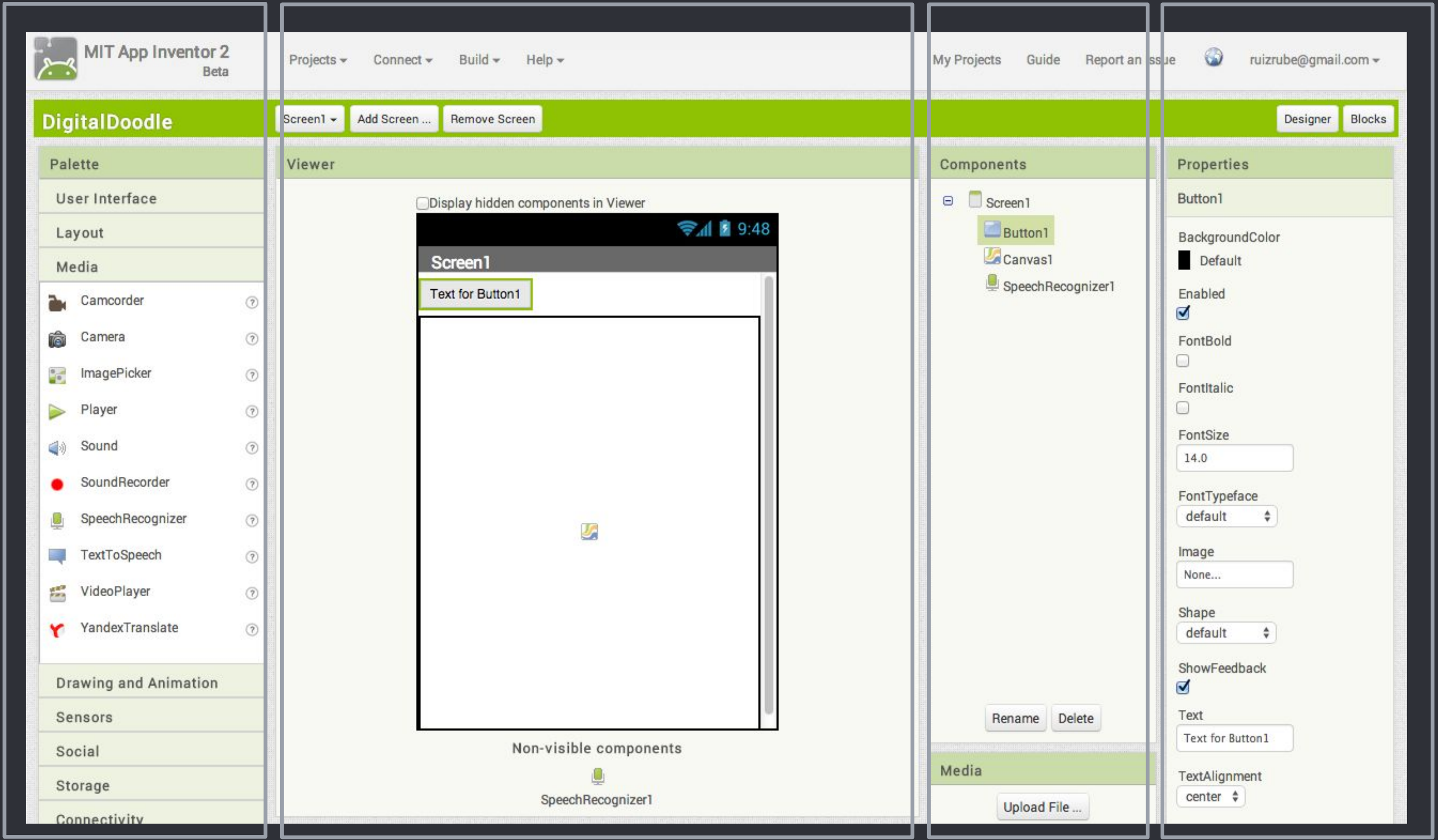

Flujo de trabajo
Diseño de la interfaz de usuario

Componentes

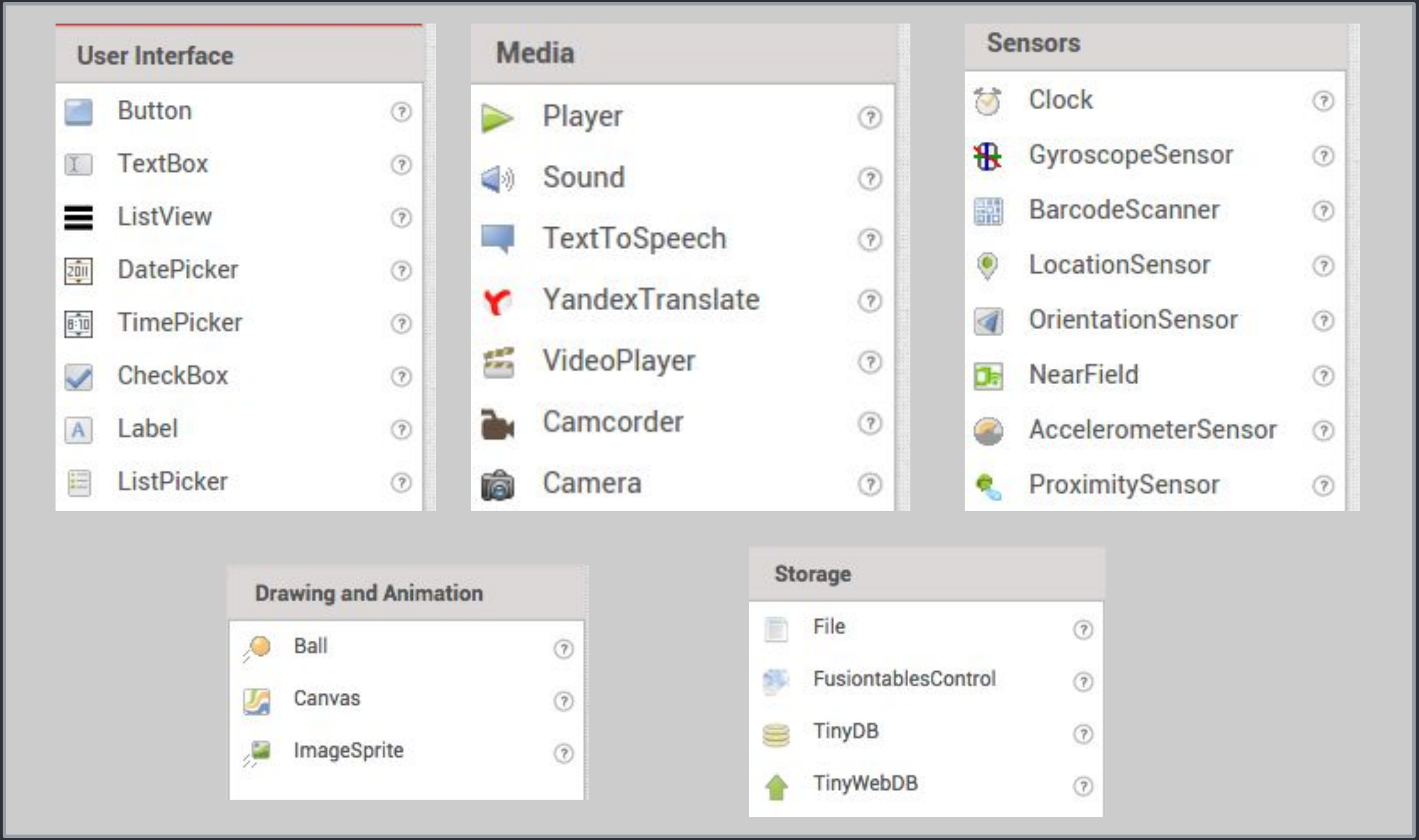

Flujo de trabajo
Diseño del comportamiento

Categoría de bloques

Bloques disponibles

Espacio de trabajo

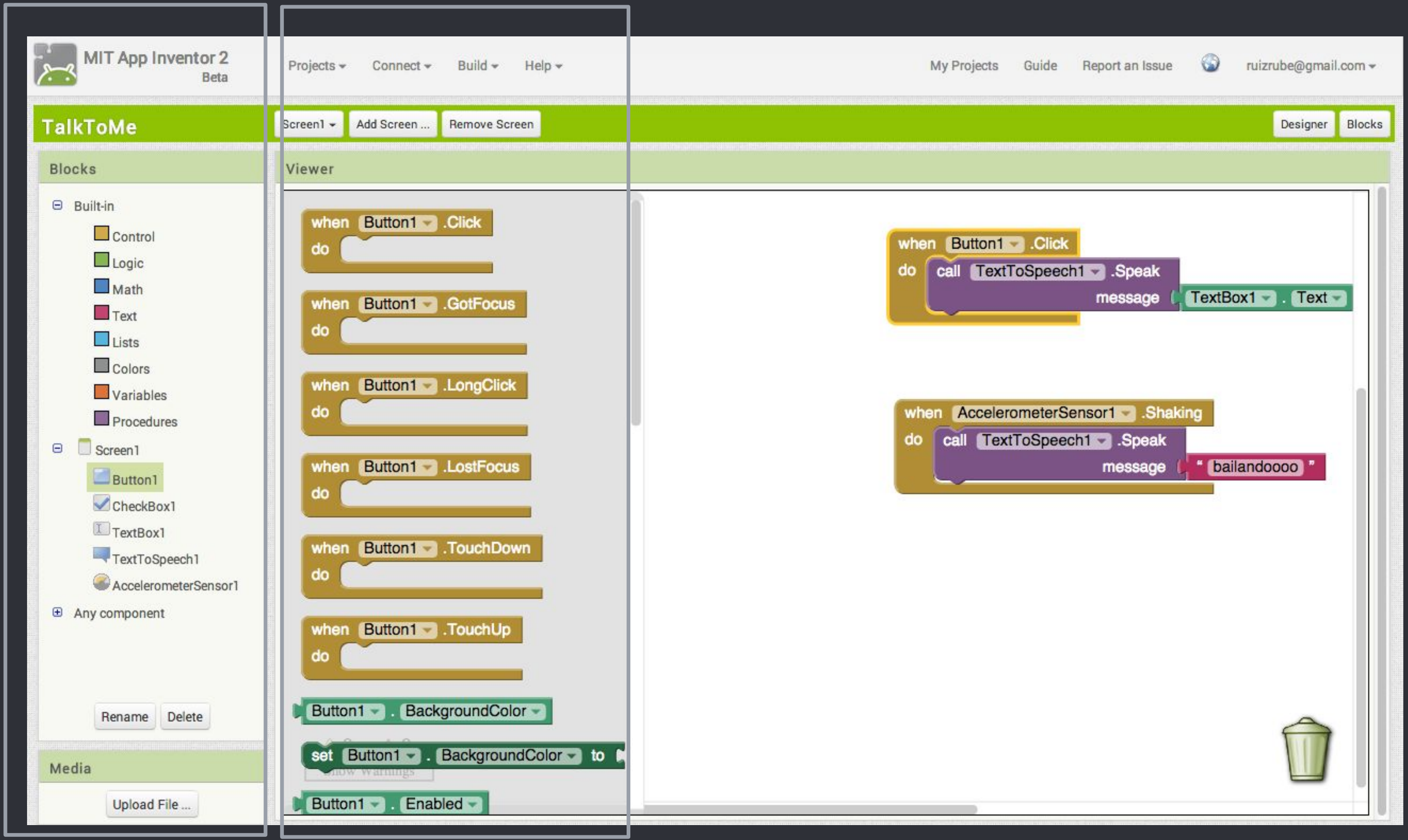

Flujo de trabajo
Diseño del comportamiento

Bloques

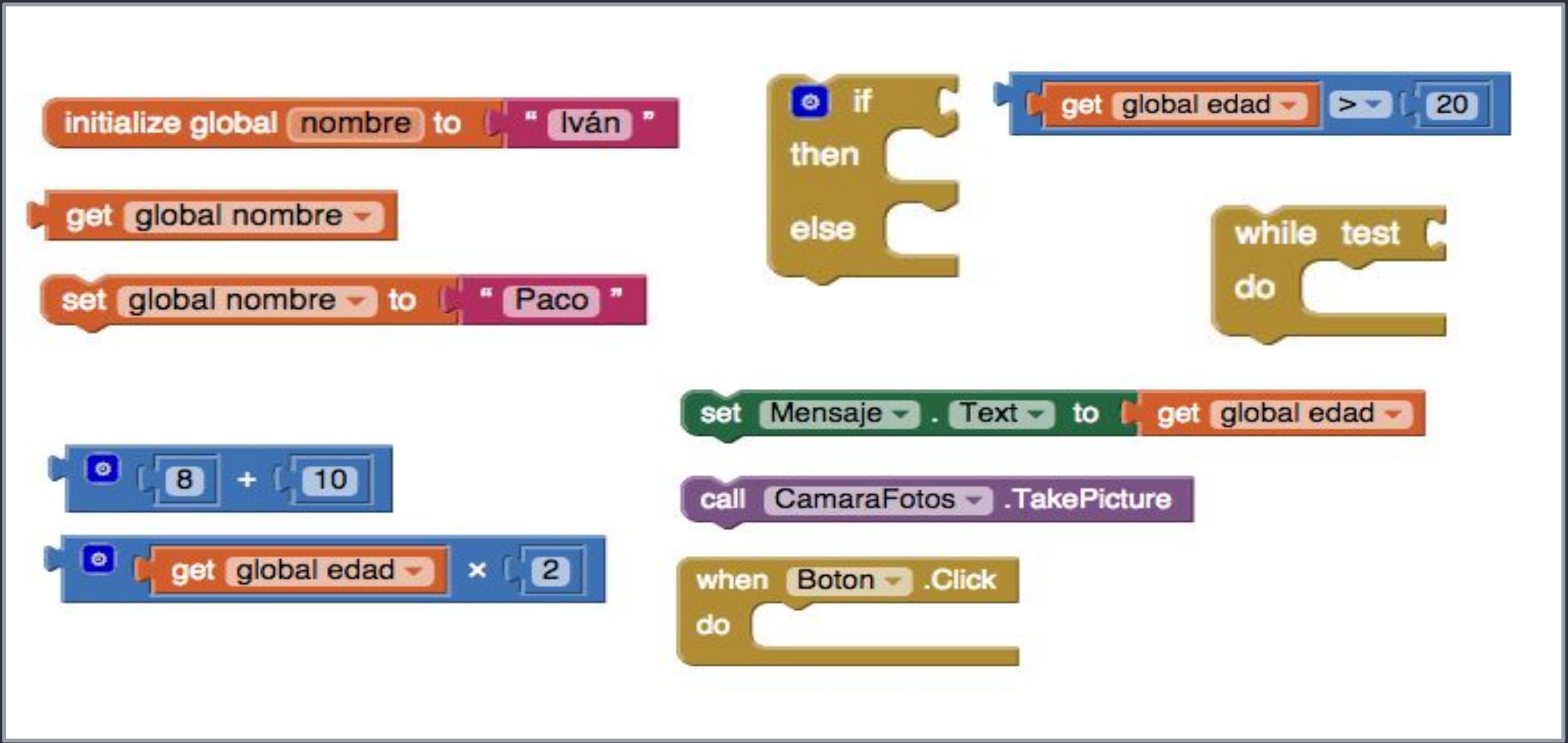

Eventos y métodos
Ciclo de procesamiento de eventos

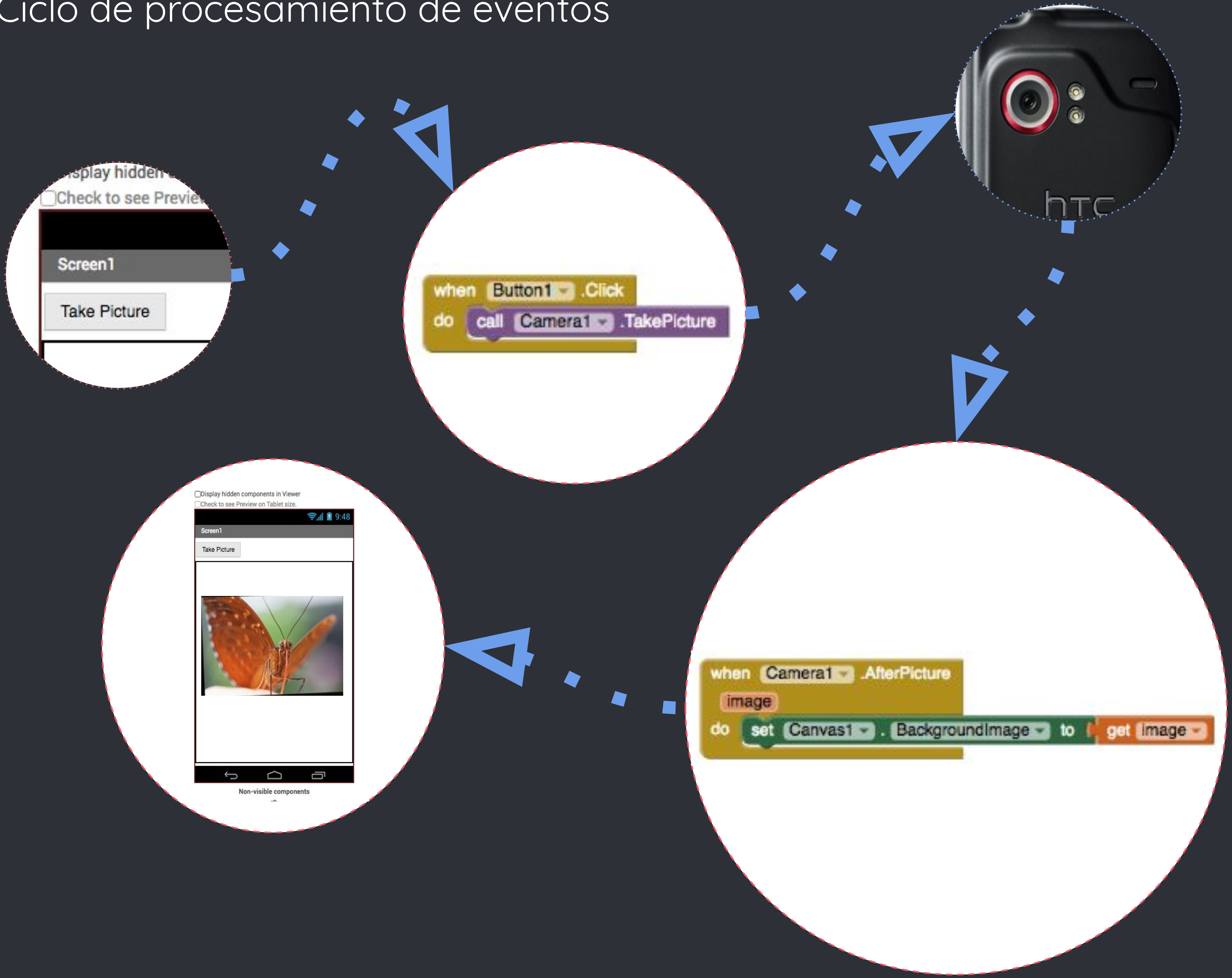

Flujo de trabajo
Pruebas de la aplicación

Nos conectamos por AI Companion dentro de la opción Connect.

Esto nos genera un código QR para nuestra aplicación

Con Vedils Companion escaneamos el código QR.

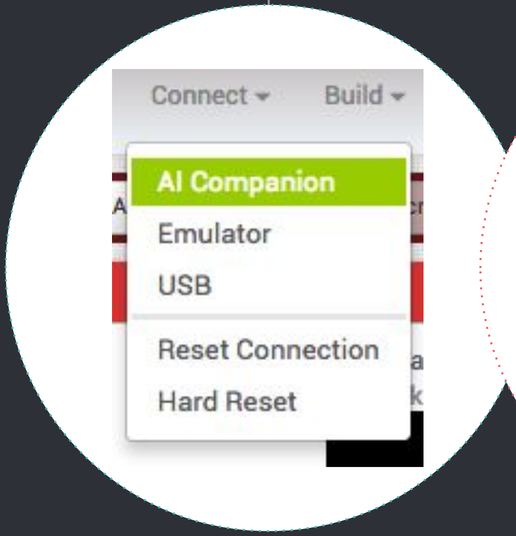

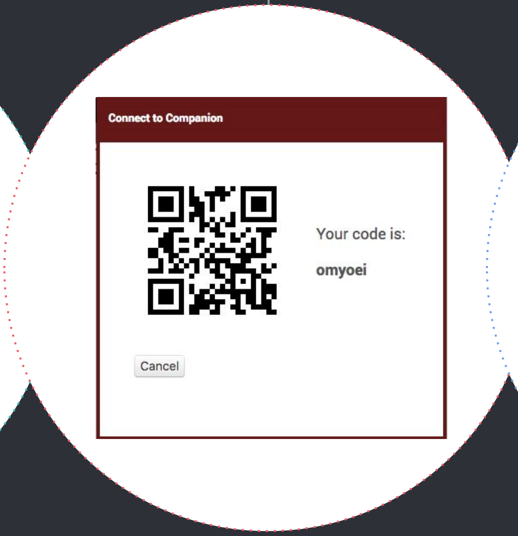

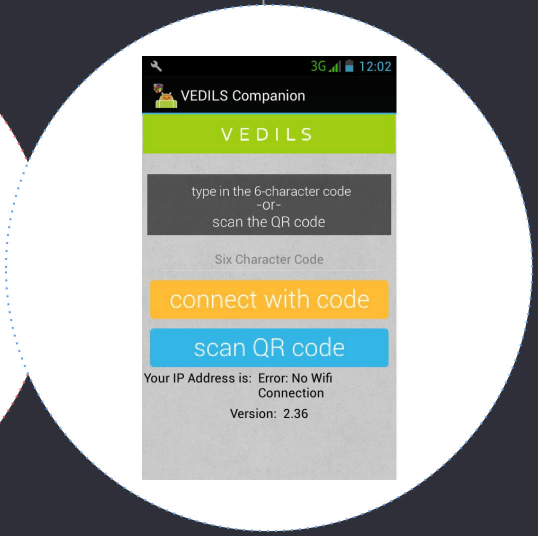

https://play.google.com/store/apps/details?id=es.uca.vedils

Flujo de trabajo
Despliegue de la aplicación

Generamos código QR. Entramos en Build (provide QR code for .apk)

Con app para leer código QR leemos el código. Podemos utilizar Vedils Companion

La aplicación estará instalada en nuestro dispositivo

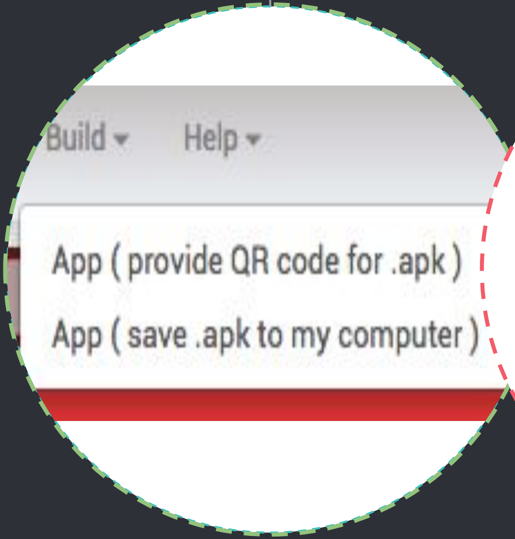

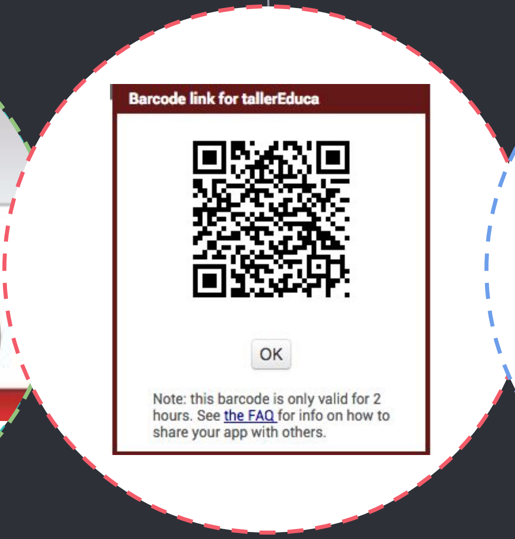

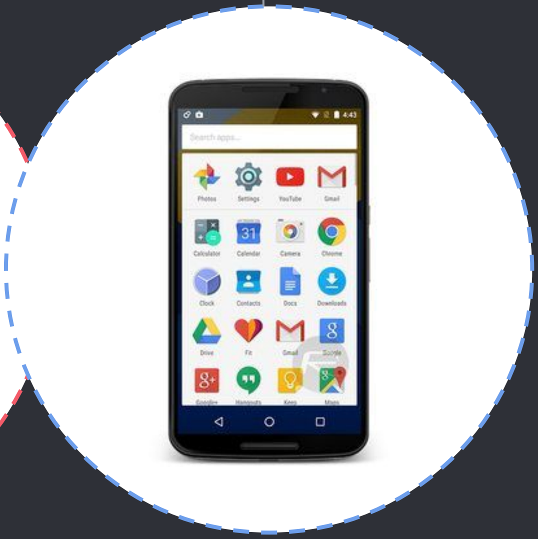

Entorno

Primero debemos preparar nuestro entorno de desarrollo y pruebas

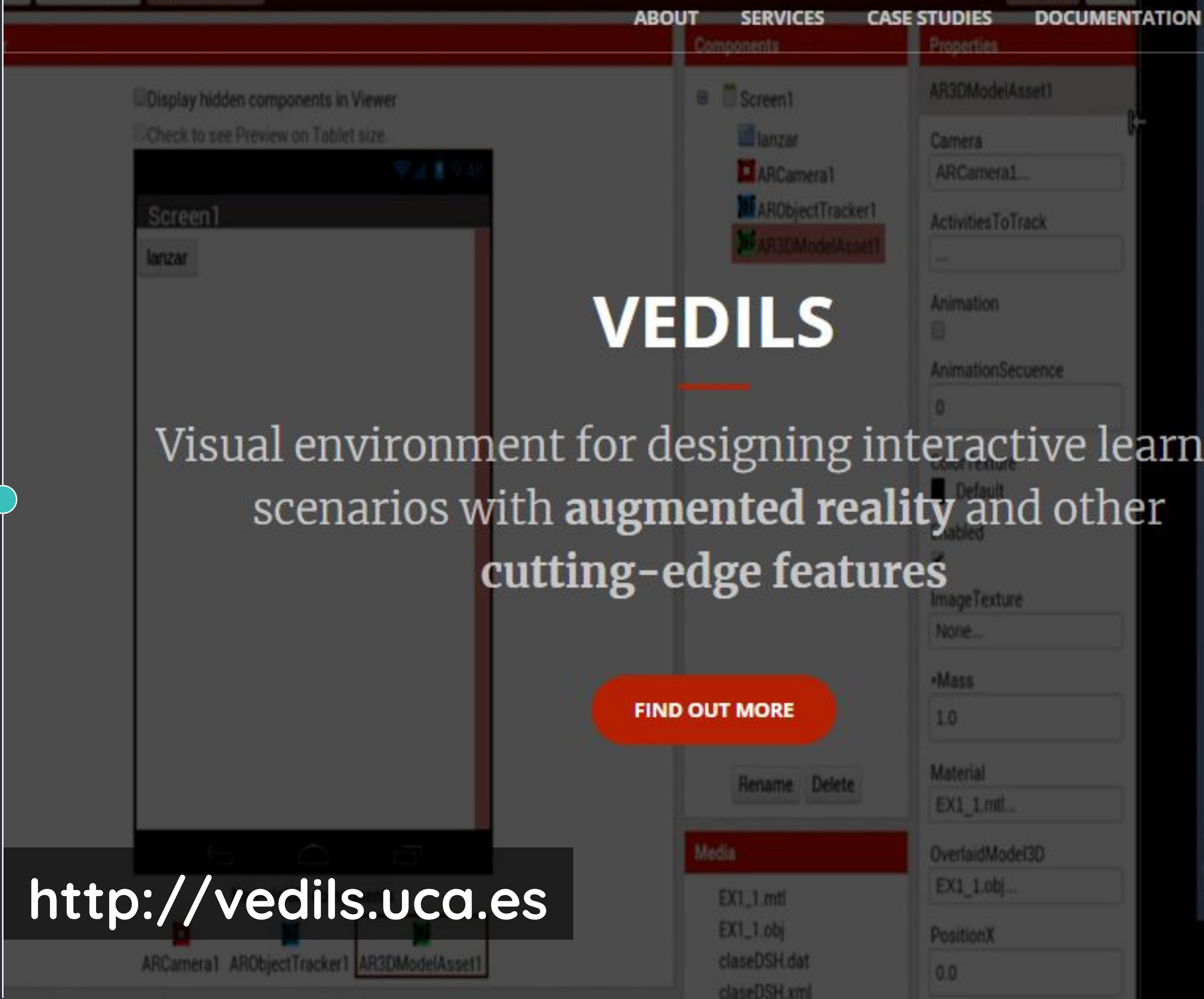

http://vedils.uca.es

AppInventor vs. VEDILS

Realidad Aumentada
Analíticas de aprendizaje
Interacciones multimodales
Comunicaciones
Robótica
Conocimiento
Realidad Virtual

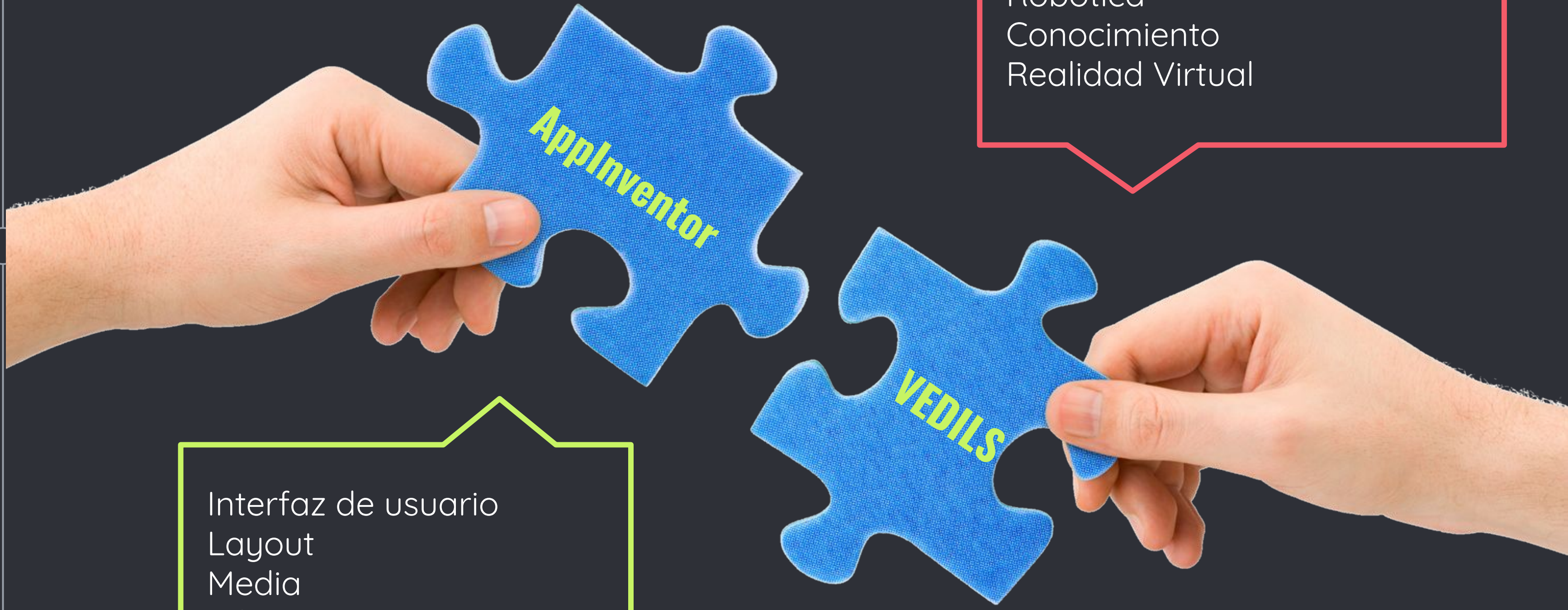

Interfaz de usuario
Layout
Media
Dibujos y animaciones
Sensores
Social
Almacenamiento
Conectividad

DISCLAIMER

VEDILS es una herramienta desarrollada por miembros del grupo de investigación SPI-FM

Es una plataforma en versión **BETA**, esto es, en proceso de pruebas y mejora

No se ofrece ningún CAU, ni soporte técnico

Pero sí nuestra **colaboración** para participar en proyectos conjuntos

SPI&FM
Software Process Improvement and Formal Methods group

Entorno de pruebas (dispositivo móvil)

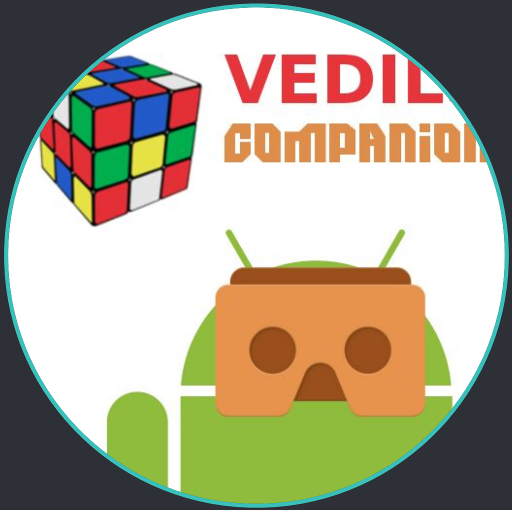

Instalar VEDILS Companion

Para poder depurar errores o ver como va quedando nuestra aplicación en mientras la diseñamos necesitamos instalar esta aplicación desde Google Play. Deberemos buscar **VEDILS companion**

Permitir aplicaciones de terceros

Para poder instalar nuestras aplicaciones debemos permitir en nuestro dispositivo la instalación de aplicaciones de terceros. La localización de esta opción depende de la versión de Android y del fabricante del dispositivo. Suele estar en:
Ajustes -> General -> Seguridad -> Orígenes desconocidos

Comprueba que estás conectado a alguna red WIFI de la UCA

Entorno de desarrollo (dispositivo de escritorio)

Acceder desde un navegador web (Google Chrome, preferiblemente) a vedils.uca.es
Pulsar en **Try It!** y luego en VEDILS **Authoring tool.**
Posteriormente deberá acceder con las credenciales proporcionadas.

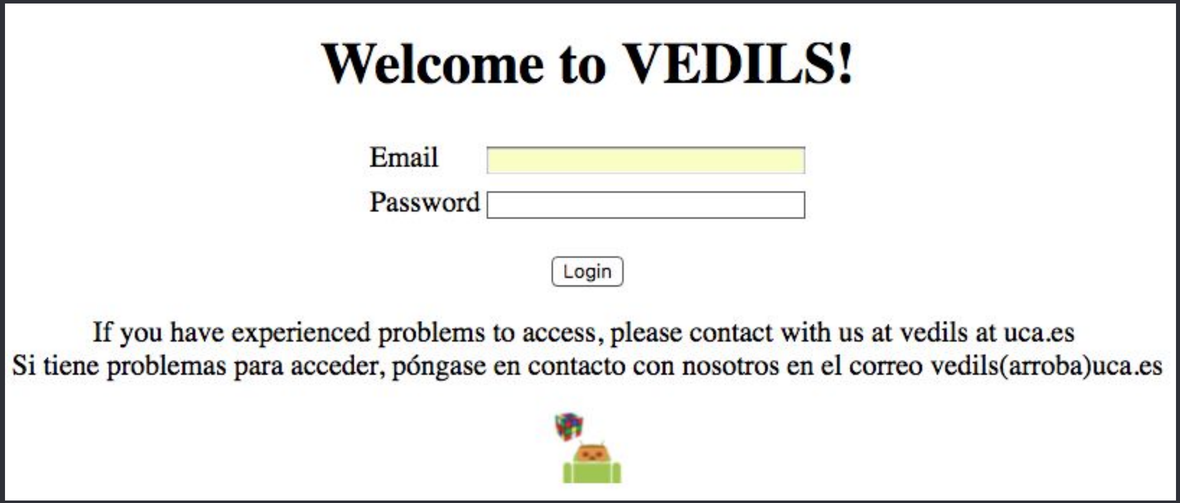

4 Ejercicios

Realizaremos ahora algunos casos prácticos

1

Ejercicio

Elementos que vamos a ver en este ejercicio

- Fundamentos de programación
  - Componentes e instancias
  - Propiedades
  - Invocación a funciones
  - Manejo de eventos
- Programación para Android
  - Controles de IU: botones, cajas de texto, etiquetas
  - Layout: vertical y horizontal

Ejercicio 1
Resultado

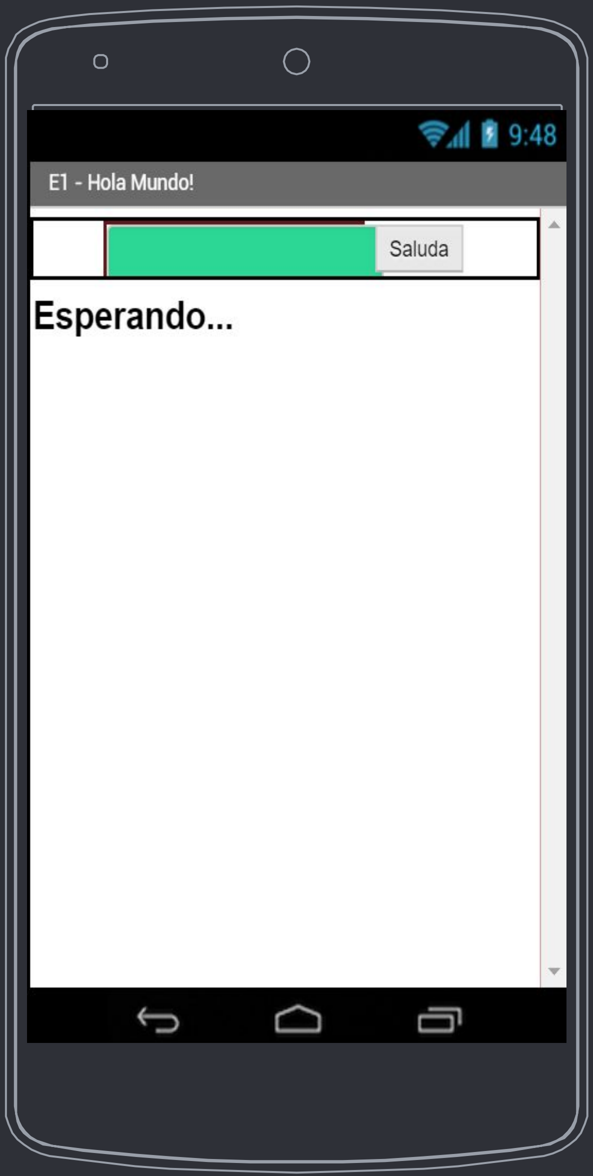

Ejercicio 1

Componentes

- Screen
  - HorizontalArrangement
    - TextBox
    - Button
  - Label

Ejercicio 1
Comportamiento

<u>Evento *clic* en **Button**</u>

1. Unir palabras (*join*) “Hola ” + nombre (TextBox.text)
2. Cambiar propiedad *Text* del **Label** al resultado

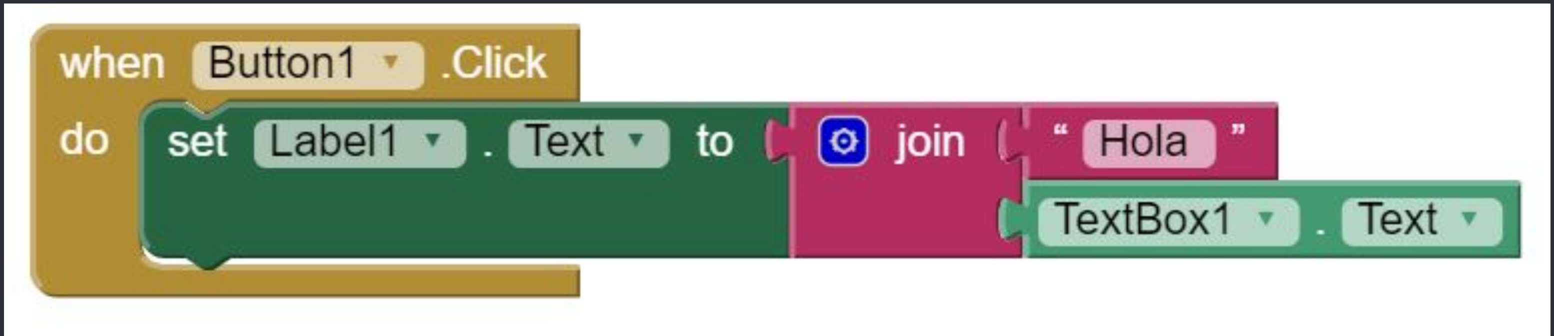

Flujo de trabajo
Pruebas de la aplicación

Nos conectamos por AI Companion dentro de la opción Connect.

Esto nos genera un código QR para nuestra aplicación

Con Vedils Companion escaneamos el código QR.

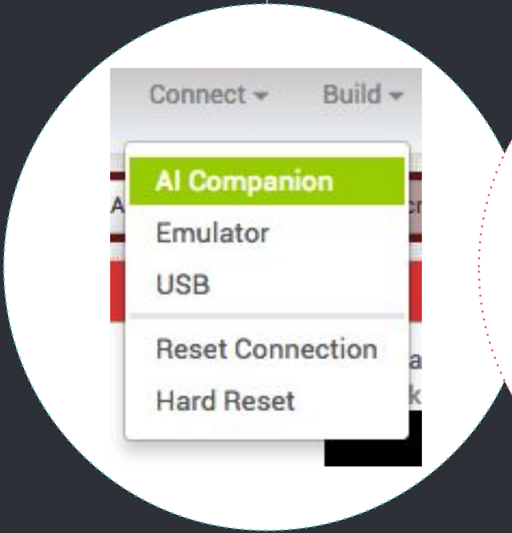

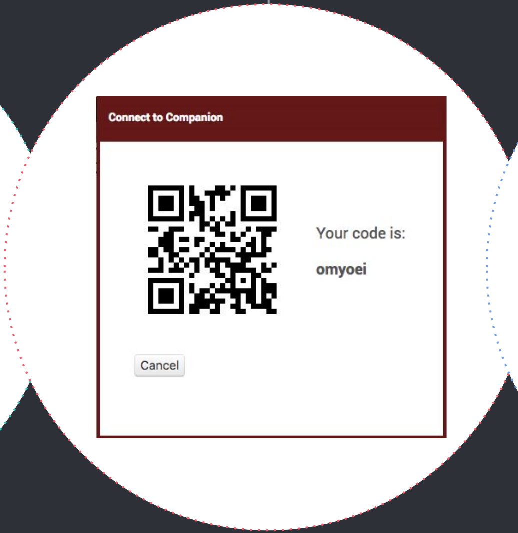

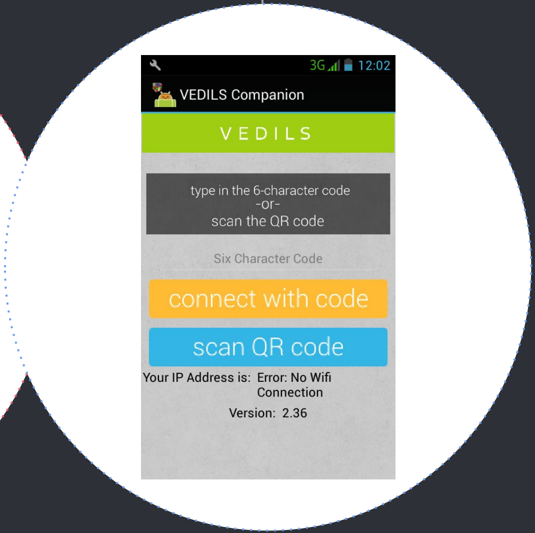

https://play.google.com/store/apps/details?id=es.uca.vedils

Flujo de trabajo
Despliegue de la aplicación

Generamos código QR. Entramos en Build (provide QR code for .apk)

Con app para leer código QR leemos el código. Podemos utilizar Vedils Companion

La aplicación estará instalada en nuestro dispositivo

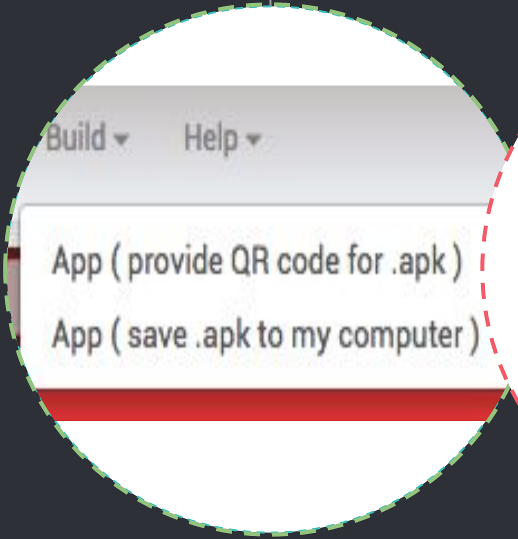

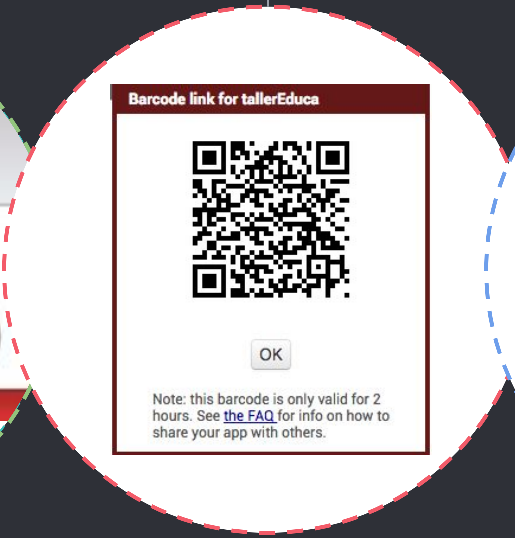

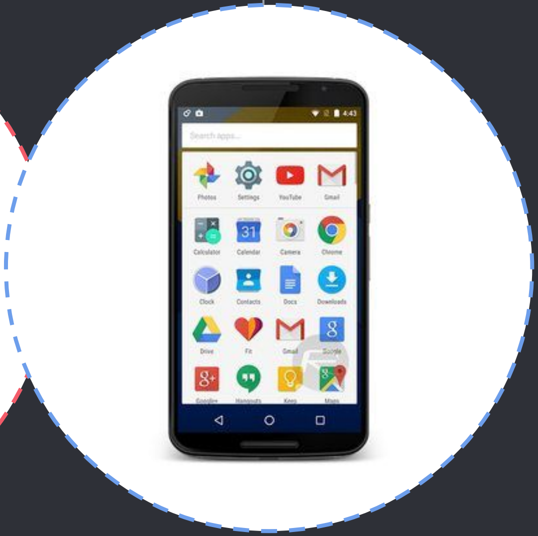

2

Ejercicio

Elementos que vamos a ver en este ejercicio

- Fundamentos de programación
    - Instrucciones condicionales
- Programación para Android
    - Personalización de la aplicación: icono, botones, colores, logo, etc.
    - Multimedia: cámara, reconocedor y sintetizador de voz
    - Social: compartir

Ejercicio 2
Resultado

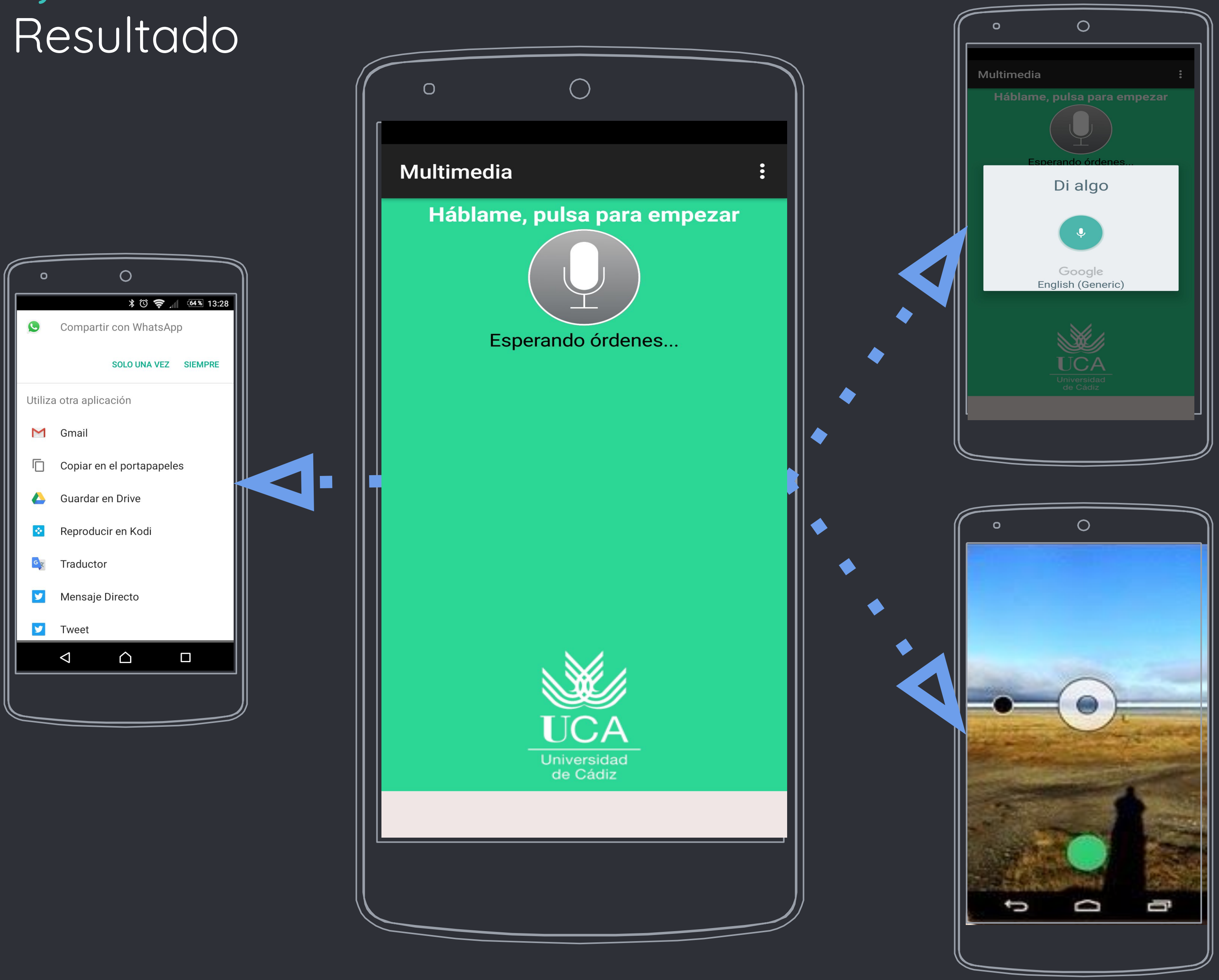

Ejercicio 2
Componentes

- Screen
  - Vertical Arrangement
    - Label
    - Button (Con imagen de 100x100)
    - Label
  - Vertical Arrangement
    - Image
- Componentes no visibles
  - SpeechRecognizer
  - Camera
  - TextToSpeech
  - Sharing
- Recursos
  - Fichero icono de la app
  - Logo de la UCA

Ejercicio 2
Comportamiento

<u>Evento *clic* en **Button**</u>

1. Invocar a *GetText* del **SpeechRecognizer**

<u>Evento *AfterGettingText* en **SpeechRecognizer**</u>

1. Si el usuario ha dicho “saluda”
   a. Entonces invocar a *Speak* del **TextToSpeech**
2. Si el usuario ha dicho “foto”
   a. Entonces invocar a *TakePicture* del **Camera**
3. Si el usuario ha dicho “compartir”
   a. Entonces invocar a *ShareMessage* del **Sharing**
4. En otro caso
   a. Cambiar propiedad *Text* del **Label**

<u>Evento *AfterPicture* en **Camera**</u>

1. Establecer la imagen de fondo Background de la Screen

Ejercicio 2
Bloques

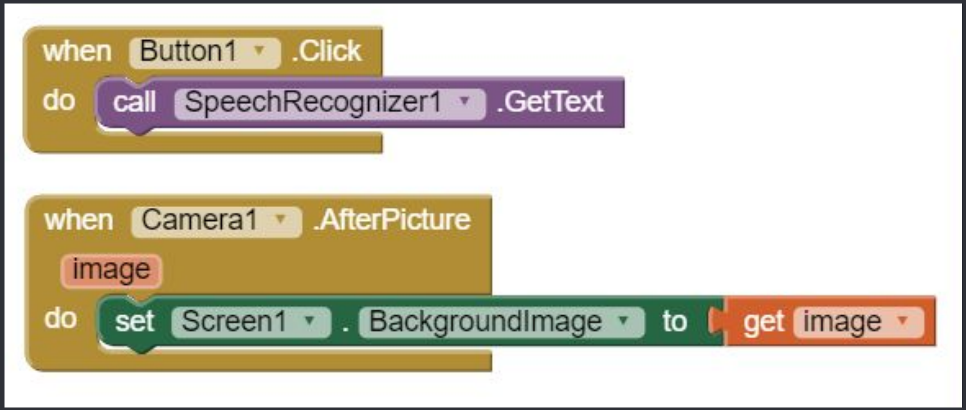

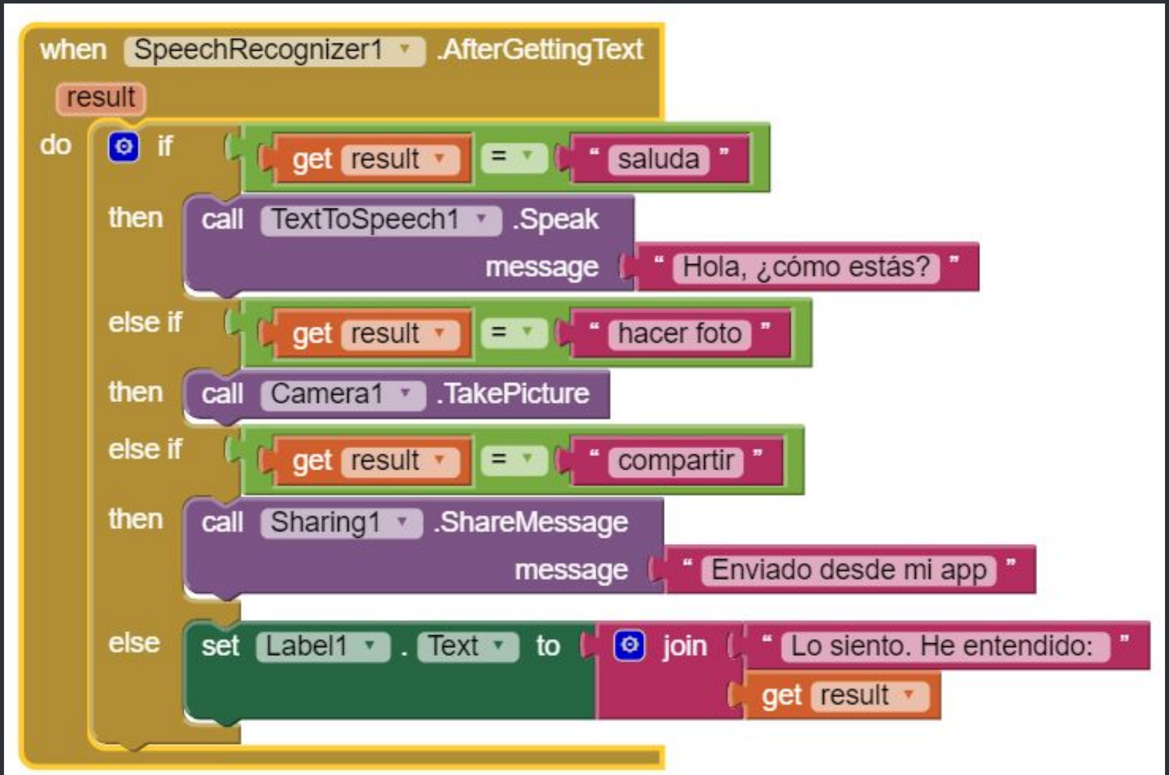

3

Ejercicio

Elementos que vamos a ver en este ejercicio

- Fundamentos de programación
  - Variables
  - Procedimientos
  - Funciones matemáticas, de listas y de flujo de datos
  - Instrucciones repetitivas
- Programación para Android
  - Interfaz de usuario: notificaciones
  - Multimedia: sonido
  - Sensores: acelerometro

Ejercicio 3
Resultado

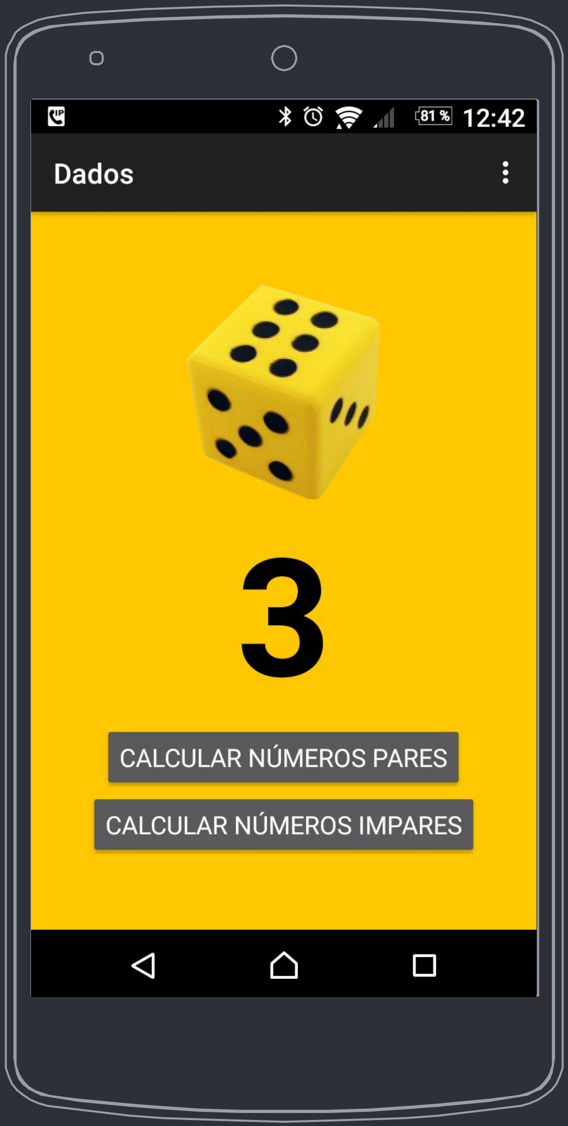

Ejercicio 3
Componentes

- Screen
  - Button (x2)
  - Label
- Componentes no visibles
  - Notifier
  - Accelerometer
  - Sound
- Recursos
  - Imagen del *dado*
  - Fichero de sonido del *dado*

Ejercicio 3
Comportamiento y bloques

¿Donde guardamos cada una de las tiradas?

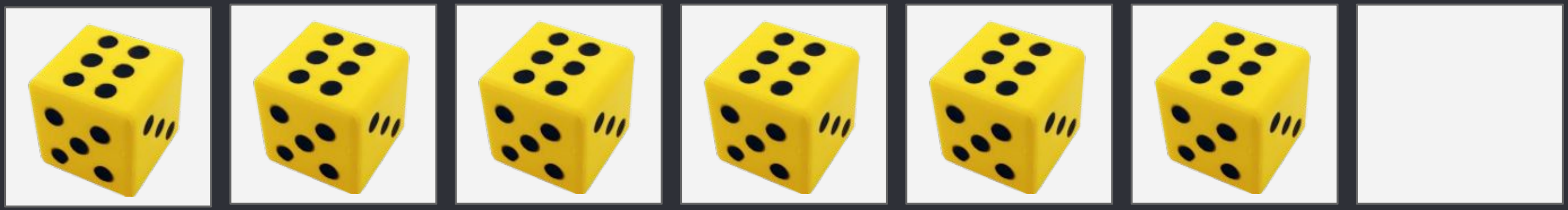

__Variable:__ espacio (en bytes) en la memoria del dispositivo. Ese espacio contiene un valor que puede ser numérico, textual o compuesto (por ejemplo, una lista de palabras o de números). La variable tiene asociada un identificador (nombre simbólico) con el cual poder acceder a su contenido.

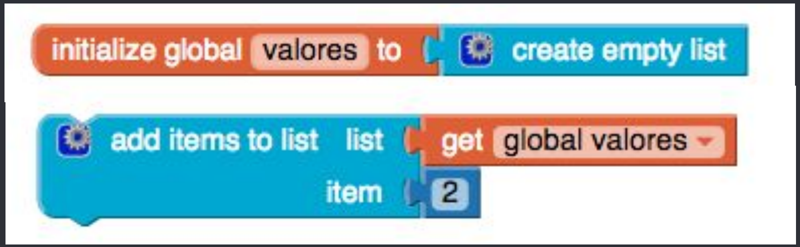

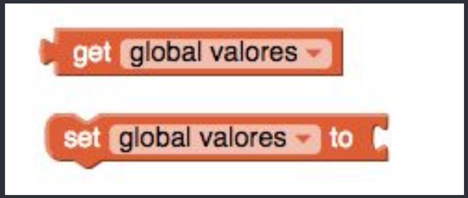

Ejercicio 3

Comportamiento y bloques

Tirar dado al pulsar el botón

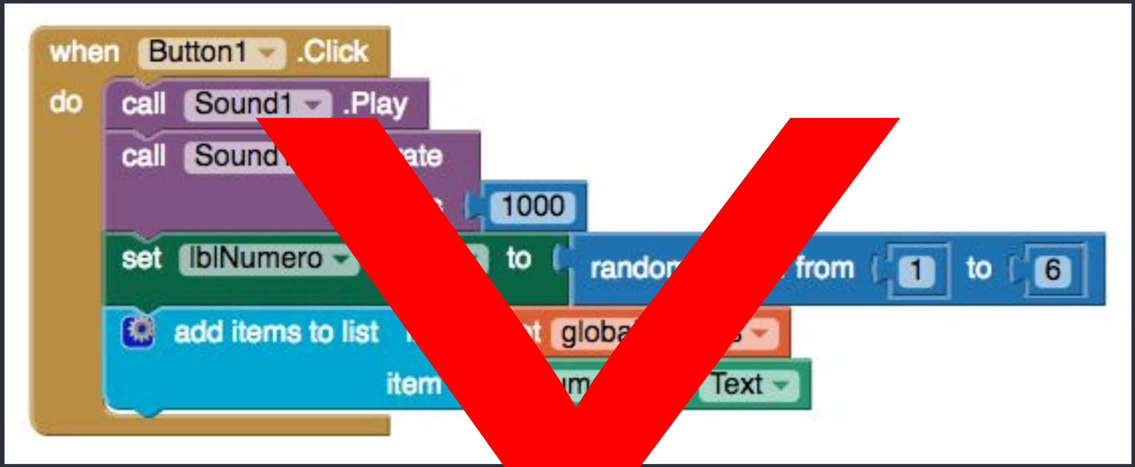

Tirar dado al “ag[illegible]óvil

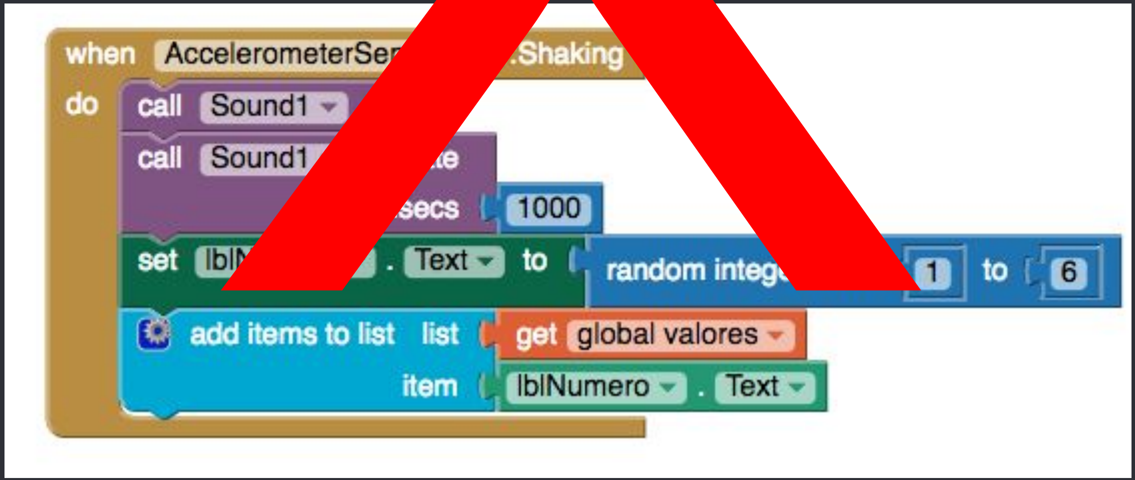

duplicación!!

Ejercicio 3
Comportamiento y bloques

Procedimiento: manera cómoda de reutilizar bloques

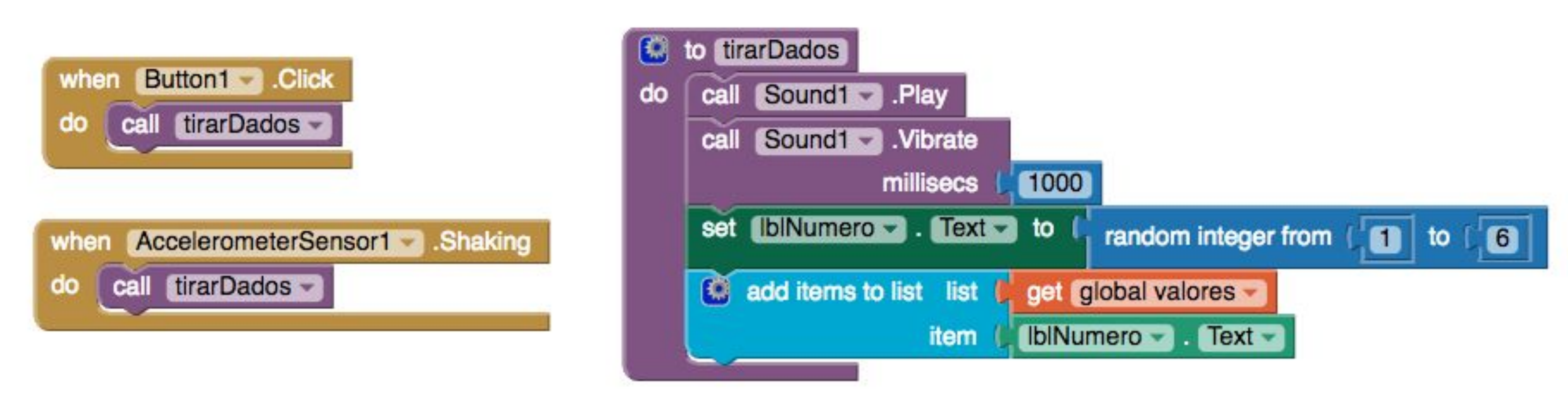

Ejercicio 3
Comportamiento y bloques

[grupos Cádiz (25/01/18), Puerto Real (01/02/18), INDESS (23/02/18)]

¿Cómo calcular cuántos **pares** han salido?
Tenemos que quedarnos sólo con aquellas tiradas que sean pares (resto -módulo- de la división entre 2 es 0) y luego contar cuántas tenemos

Filtrar flujo de datos: permite seleccionar los valores que cumplan una determinada condición

Reducir conjunto de datos: aplica una función de agregación (max, min, count, sum, avg, etc.) sobre los valores

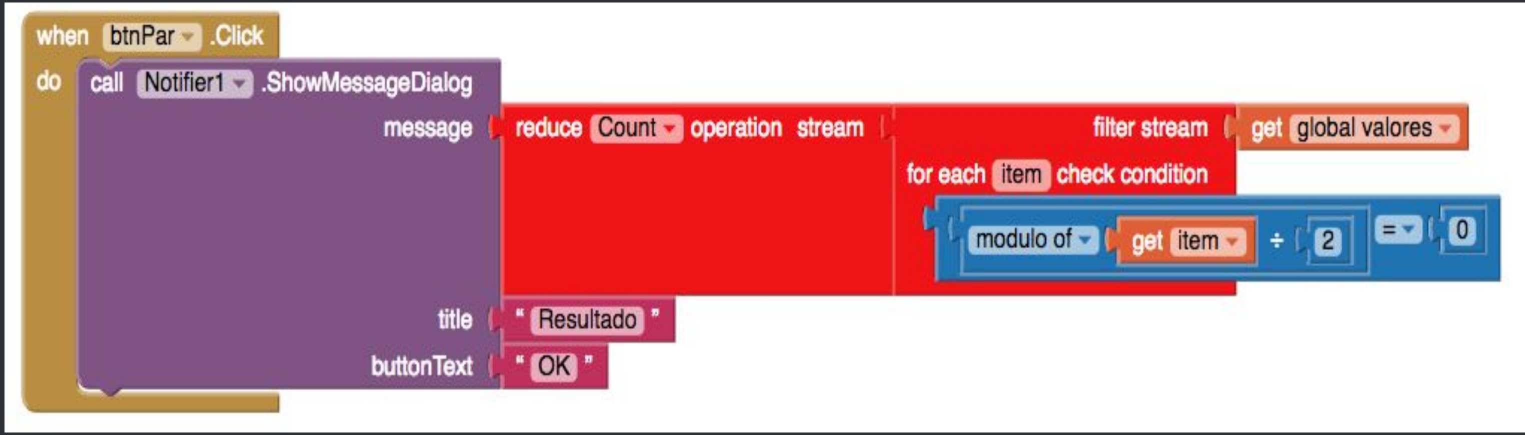

Ejercicio 3
Comportamiento y bloques

[grupos Algeciras (22/01/18), Puerto Real (23/01/18), Jerez (24 Enero 2018)]

- ¿Cómo calcular cuántos **impares** han salido?
  Tenemos que recorrer cada una de las tiradas y comprobar si el valor es impar (resto -modulo- de la división entre 2 no es 0). En caso positivo, tenemos que incrementar el contador en 1

- **Bucle**: permite para repetir acciones un número determinado o indeterminado de veces

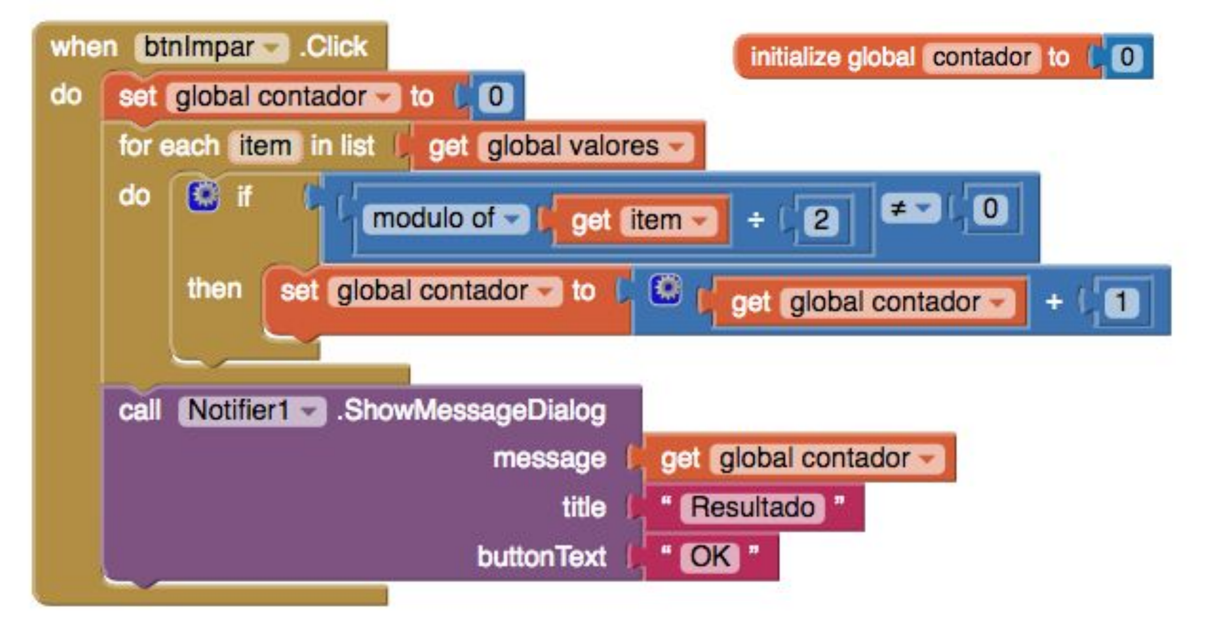

4

Ejercicio

Elementos que vamos a ver en este ejercicio

- Programación para Android
    - Múltiples pantallas
    - Sensores: ubicación y orientación
    - Dibujos y animaciones

Ejercicio 4
Resultado

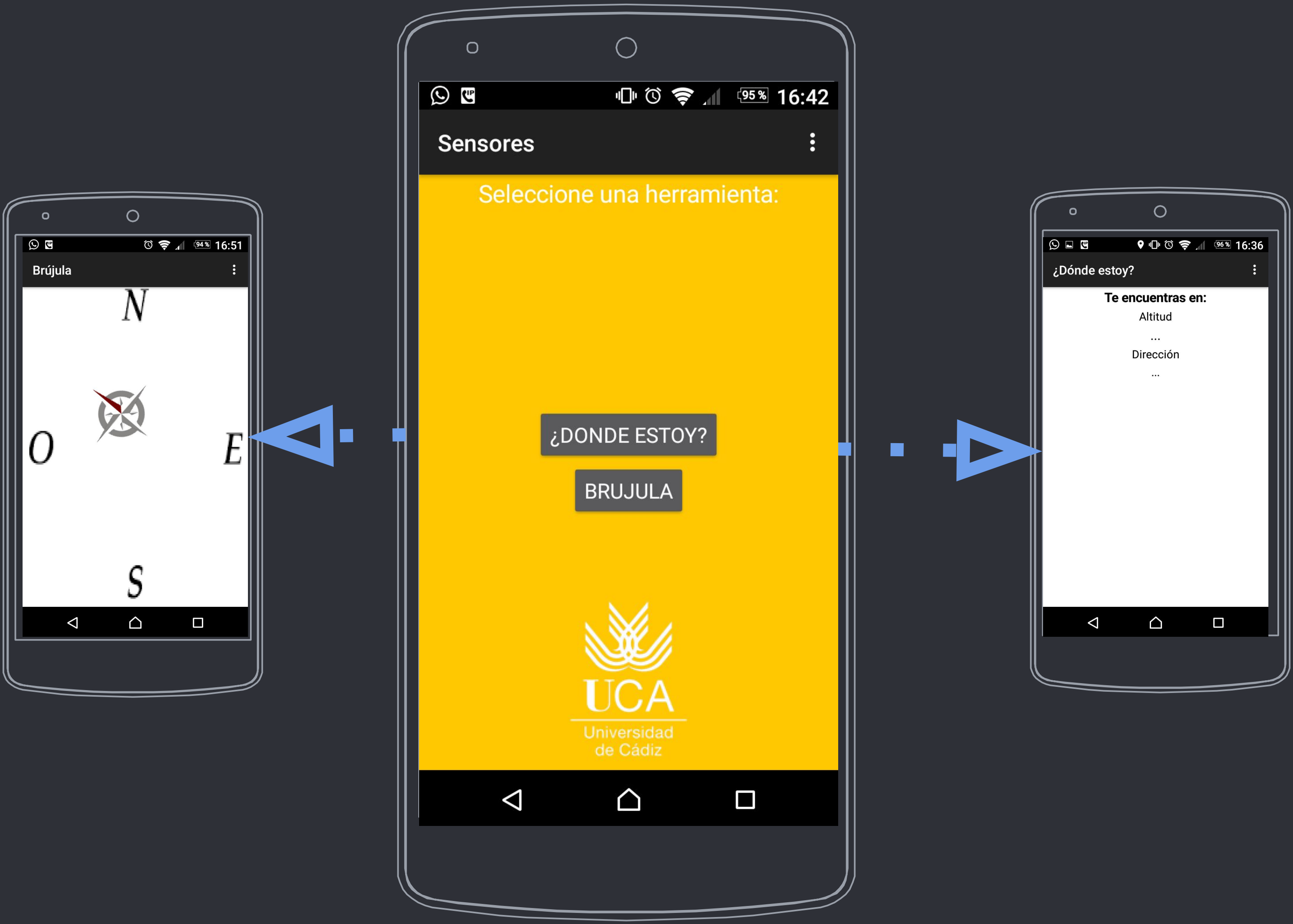

Ejercicio 4
Componentes

- Screen1
  - Label
  - Button (x2)
  - Vertical Arrangement
  - Image
- ScreenDondeEstoy
  - Label
  - LocationSensor
- ScreenBrujula
  - Canvas
  - ImageSprite
  - OrientationSensor
- Recursos
  - Fichero imagen de la *brújula*
  - Fichero imagen de la *rosa de los vientos*

Ejercicio 4

Comportamiento

Pantalla: Screen1

Evento *Click* en **ButtonDonde**

1. Abrir ventana *ScreenDondeEstoy*

Evento *Click* en **ButtonBrujula**

1. Abrir ventana *ScreenBrujula*

Pantalla: Donde

Evento *LocationChanged* en **LocationSensor**

1. Establecer la propiedad *Text* del **Label1** y **Label2** con las propiedades *Altitude* y *CurrentAddress* del **LocationSensor**

Pantalla: Brujula

Evento *OrientationChanged* en **OrientationSensor**

1. Establecer el *Heading* de la **ImageSprite** con el ángulo comprendido entre el punto cardinal Norte y el dispositivo (*Azimuth*).

Ejercicio 4
Bloques

PANTALLA Screen1

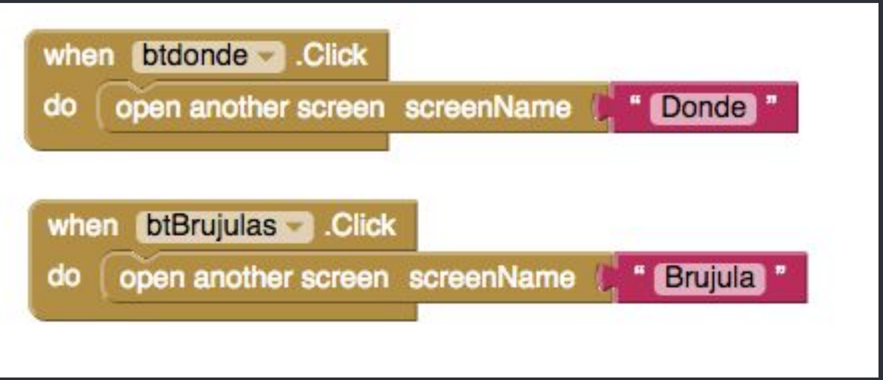

PANTALLA Brujula

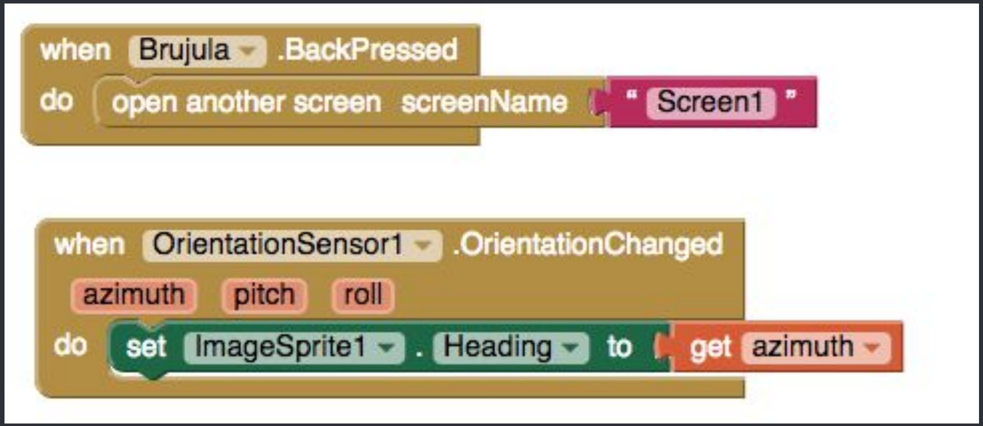

PANTALLA Donde

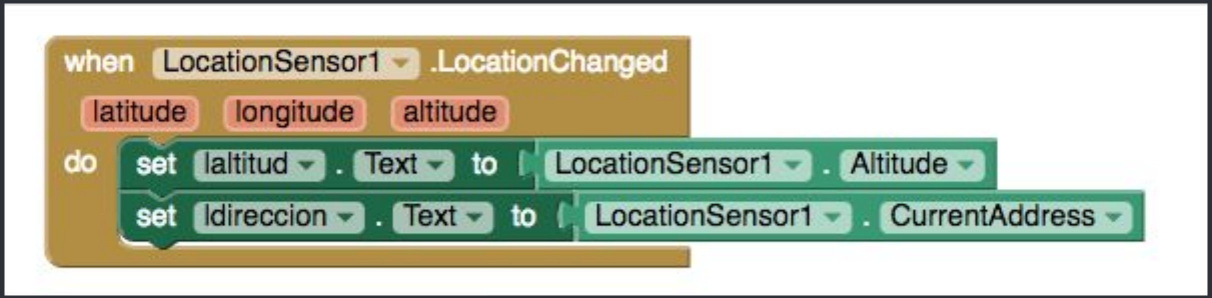

5

Ejercicio

Elementos que vamos a ver en este ejercicio

- Conceptos básicos Realidad aumentada
- Componentes de VEDILS para realidad aumentada

Demostración del app

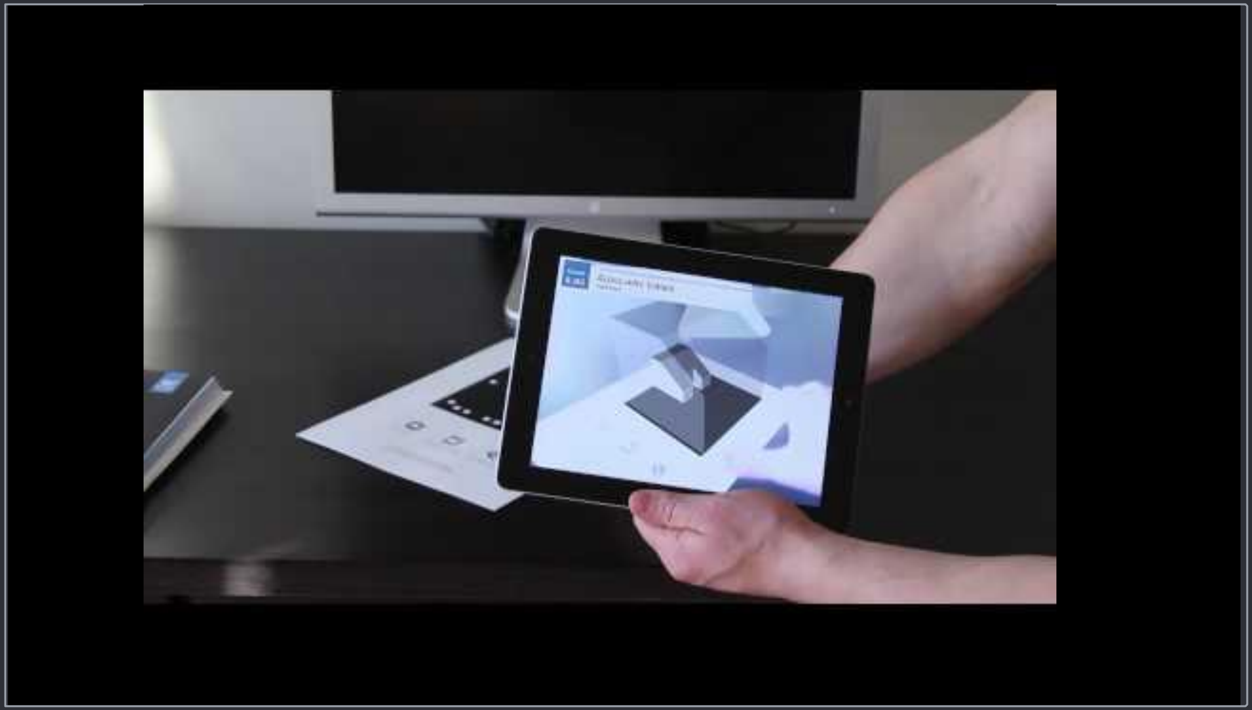

Realidad aumentada

Y otros conceptos interesantes

http://vedils.uca.es/web/vedilsAR.html

Realidad aumentada, mixta virtual

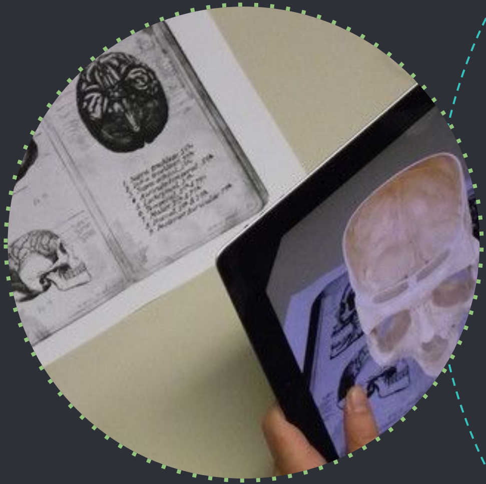

Realidad aumentada

Superponemos información sobre el mundo real

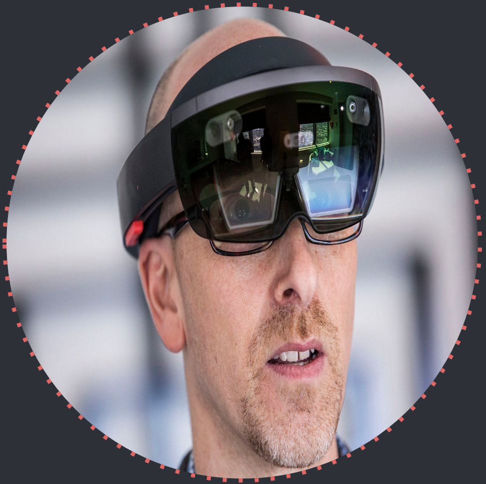

Realidad Mixta

El mundo real es reconocido, permitiendo a los elementos virtuales interactuar con este

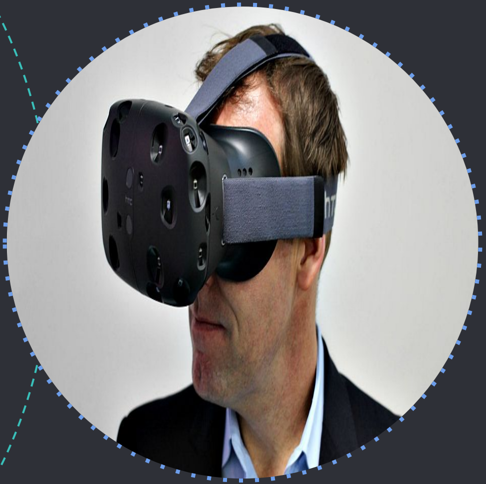

Realidad Virtual

Se sumerge al usuario en un mundo virtual ocultándose el mundo real

Realidad aumentada

Reconocimiento de nuestro entorno

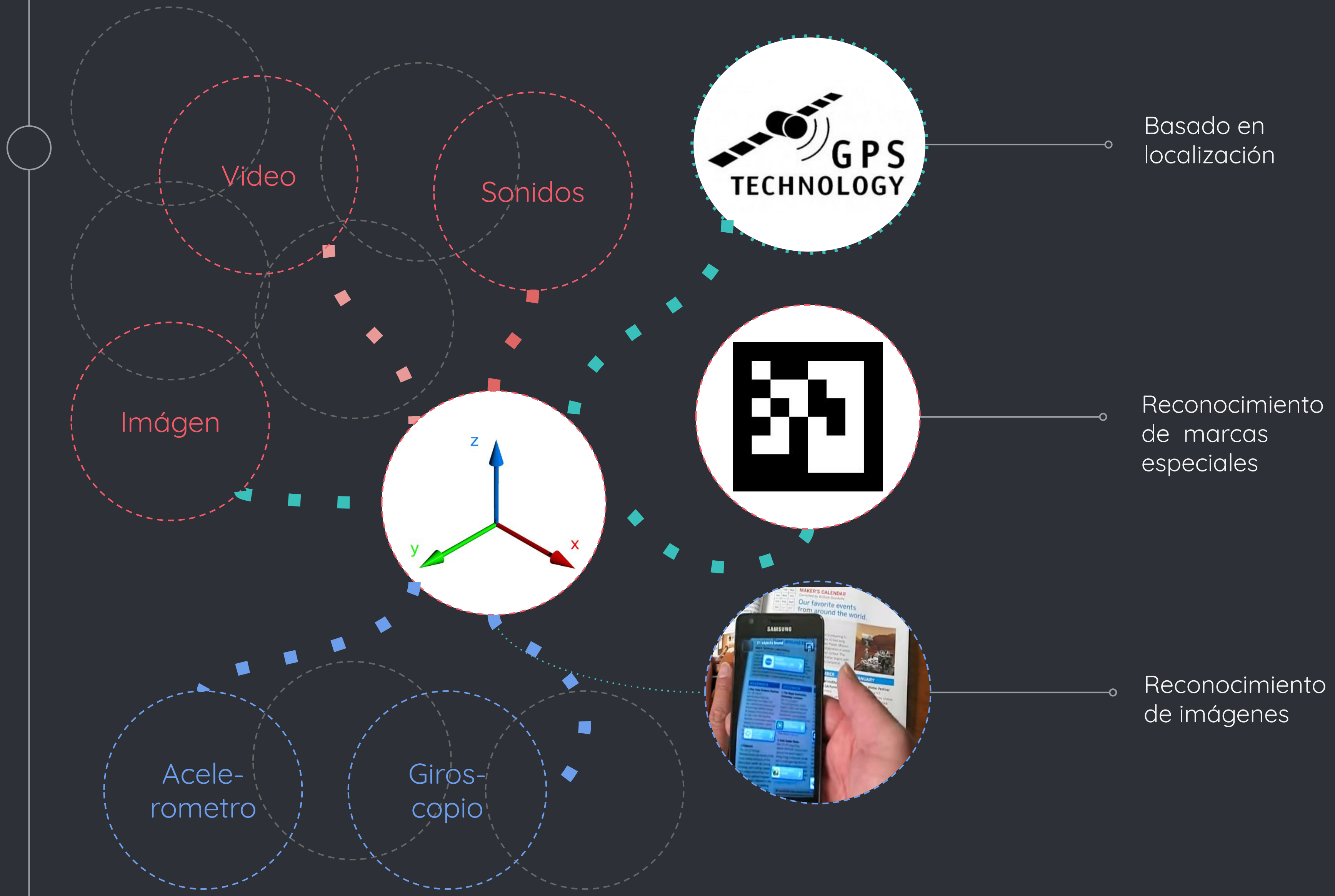

Ejercicio 5

Requisitos previos

- Deberemos tener marcas de realidad aumentada.
- Descargar la imágenes desde el campus del curso.

Ejercicio 5
Componentes

- Screen1
  - VerticalArrangement
    - Label
    - Button
- Screen2
  - VerticalArrangement
    - Label
    - Button
  - Componentes no visibles
    - ARCamera
    - ARMarkerTracker
    - AR3DModelAsset
- Recursos
  - Modelo geometría
  - Modelo material

Ejercicio 5

Lógica de la aplicación

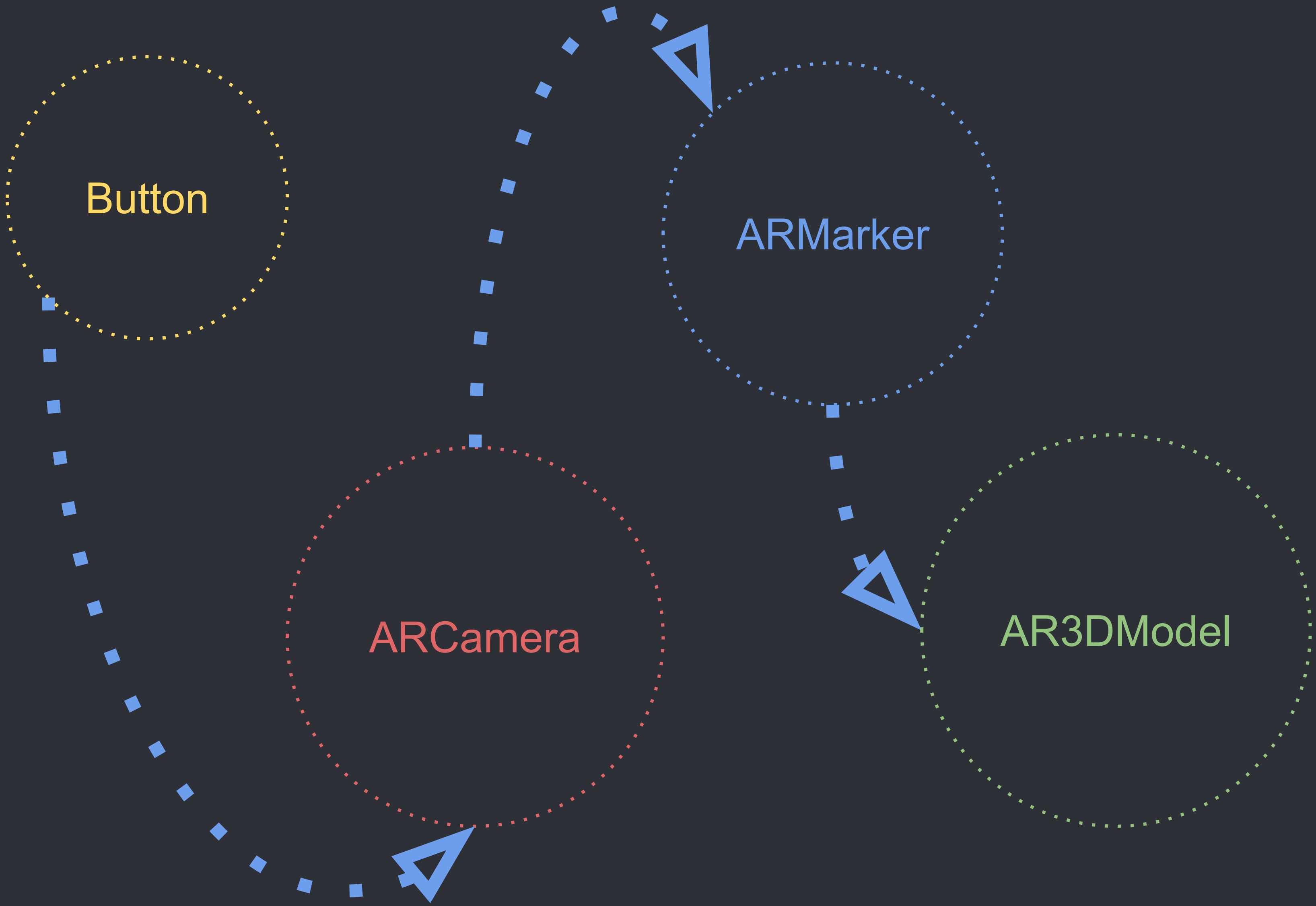

Ejercicio 5
Resultado

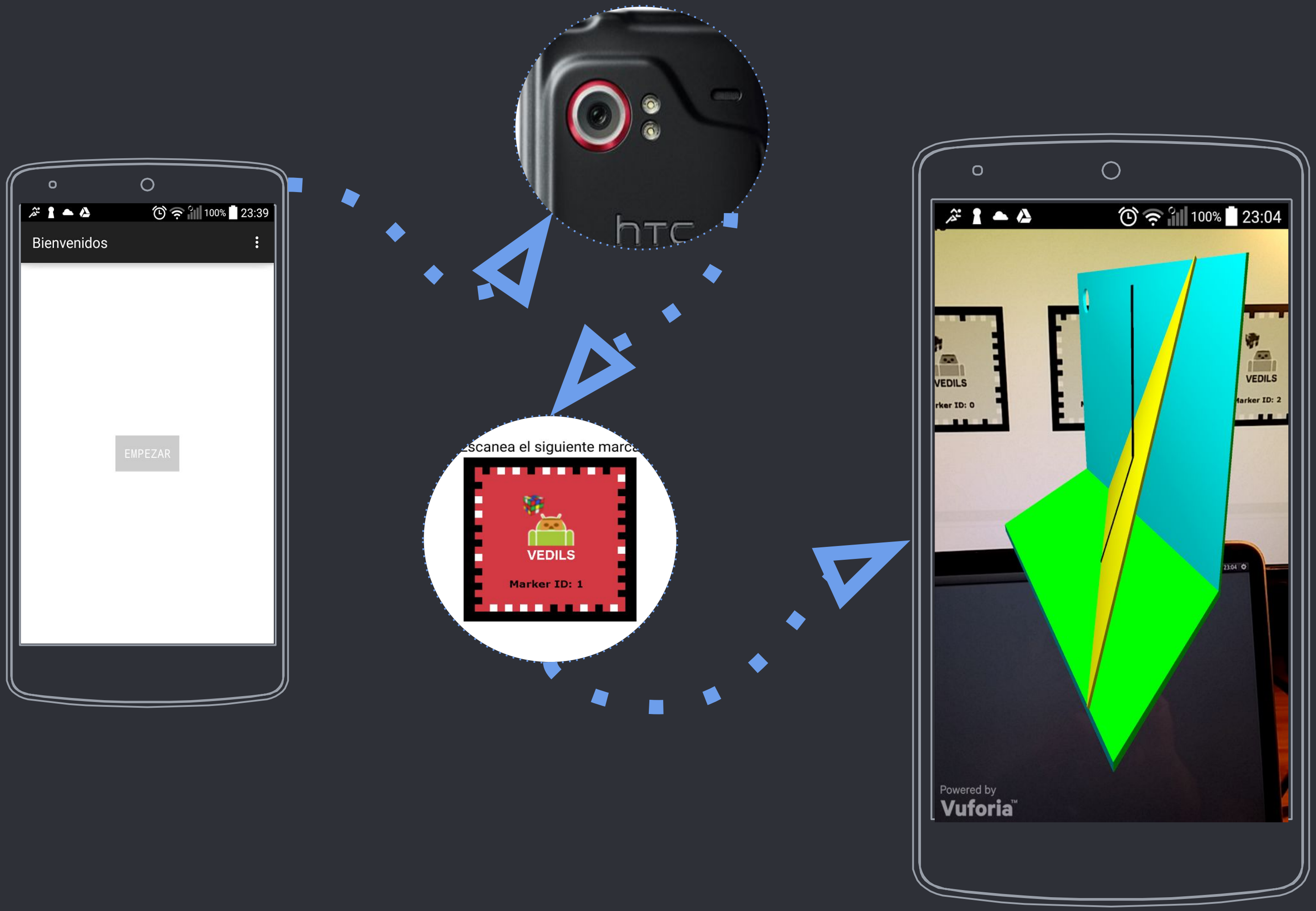

Ejercicio 5
Diseño y comportamiento

Componente AR3DModelAsset

1. Establecemos *Camera* a **ARCamera1**
2. Establecemos *Material* a **graph1.mtl**
3. Establecemos *OverlaidModel3D* a **graph1.obj**
4. Establecemos *StickTo* a **ARMarkerTracker1**

Evento *clic* en **Button**

1. Invocar a *Start* del **ARCamera**

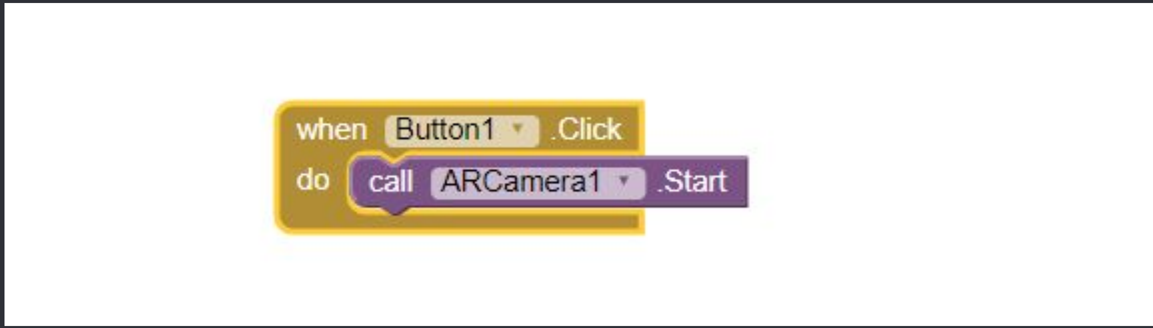

X

Tarea de evaluación

Elementos que vamos a poner en práctica

- Fundamentos de programación
  - Variables (listas)
  - Funciones de flujo de datos / bucles
- Programación para Android
  - Controles de IU: botones, etiquetas
  - Multimedia: cámara

Tarea de evaluación

Resultado esperado

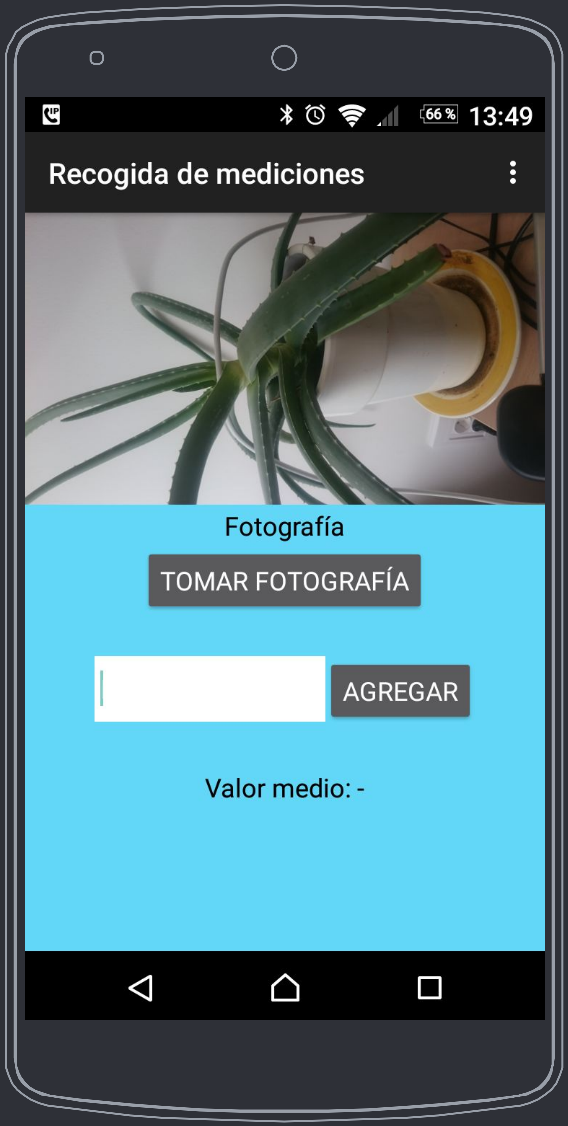

- Aplicación para la recogida de mediciones que permita:
  - Tomar una fotografía de la muestra
  - Añadir un valor numérico (medición)
  - Calcular automáticamente la media aritmética (suprimiendo los valores superiores a 10)

5

Consejos y conclusiones

Algunas ideas para mejorar nuestros desarrollos

Consejos

Construir un prototipo

Realizar un prototipo en papel o con alguna aplicación, para tener claro el flujo de nuestra aplicación.

- http://zurb.com/playground/responsive-sketchsheets

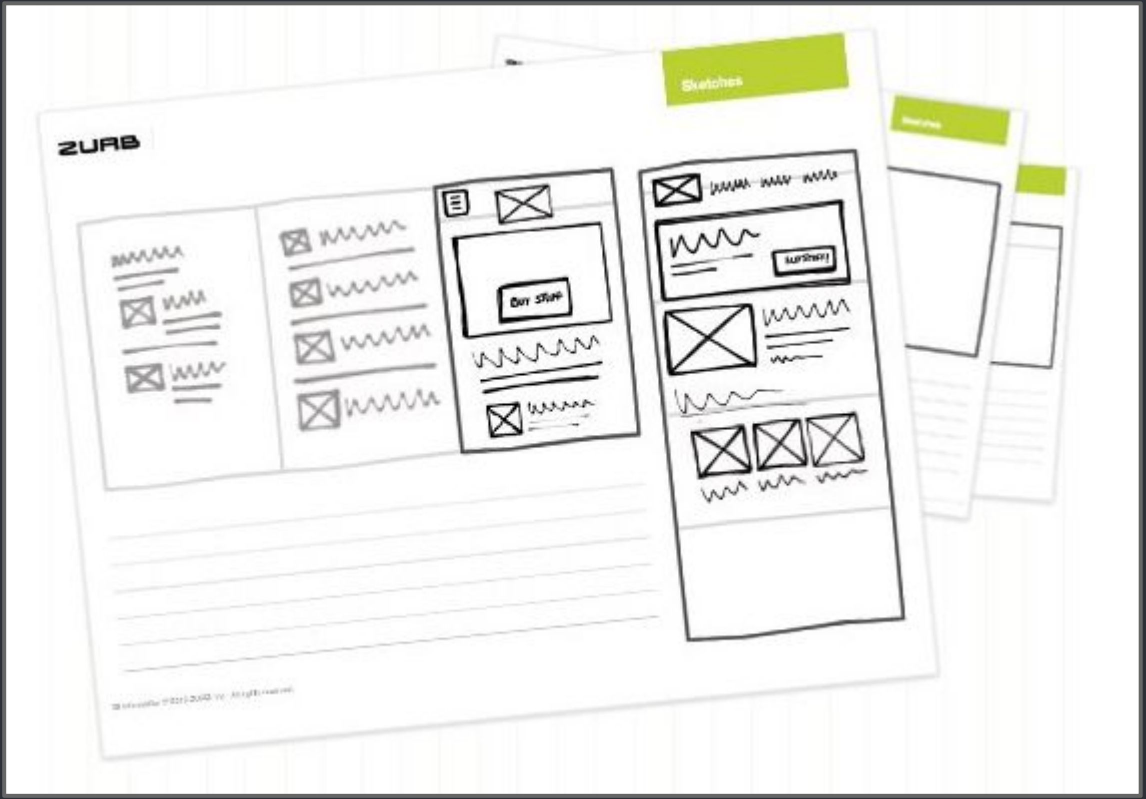

Consejos
Comentar el código

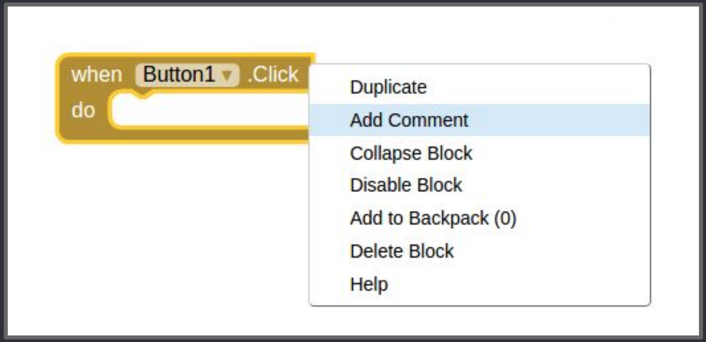

Add comment
Hacemos click con el botón derecho sobre el bloque que queramos comentar

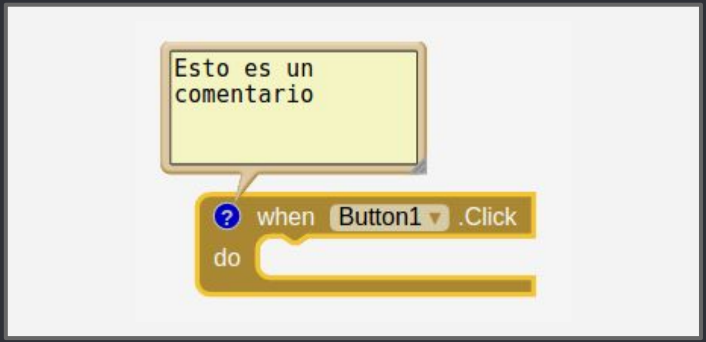

Escribir comentario
Pulsando el símbolo de interrogación podemos añadir un comentario o visualizarlo

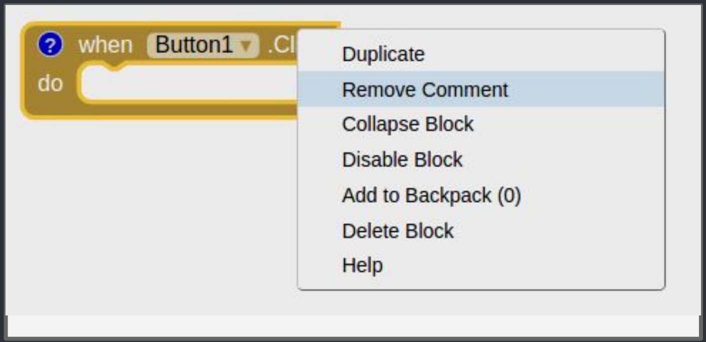

Remove comment
También podemos borrar nuestro comentario

Consejos

Otras opciones a tener en cuenta

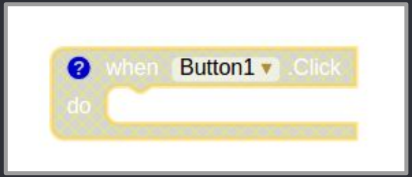

Disable Blocks

Permite deshabilitar bloques para que no se tengan en cuenta en nuestra aplicación

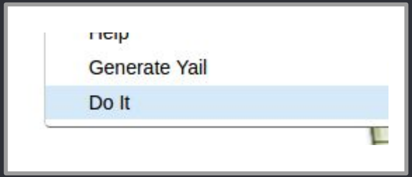

Do it

Nos permite realizar operaciones sin tener que esperar que se realice un evento que lo lance.

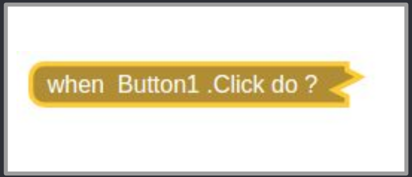

Collapse Blocks

Agrupa los bloques para mejorar la visión general de la aplicación

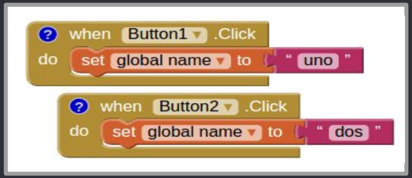

Duplicate Blocks

A veces es más sencillo duplicar un bloque que crearlo de nuevo otra vez

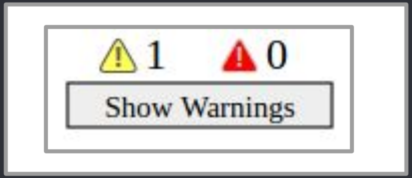

Show Warnings

Nos indica el número de advertencia y errores de nuestro código

Conclusiones finales

Con **AppInventor** podemos desarrollar de forma sencilla aplicaciones para Android.

“

VEDILS ofrece extensiones para desarrollar apps educativas enriquecidas con realidad aumentada, analíticas de aprendizaje, nuevas vías de interacción, etc

Rellenar la encuesta para poder mejorar VEDILS.
[https://goo.gl/forms/nOXJZgNBAV98cblv2]
Gracias!

¿Alguna pregunta antes de terminar?

Más información en el campus
