# Supplementary material for: Block-Based Development of Mobile Learning Experiences for the Internet of Things
Source: Sensors (Basel). 2019 Dec 11;19(24):5467. doi: 10.3390/s19245467 (PMC6960931; doi:10.3390/s19245467)
Supplement: Supplementary file 1 [file sensors-19-05467-s001.zip › Study with academics/Survey (in spanish).pdf]

VEDILS

Herramienta de autoría visual para el diseño de escenarios educativos interactivos

*Required

Encuesta sobre el desarrollo de aplicaciones para dispositivos móviles con VEDILS.

Esta encuesta es totalmente anónima, quedando garantizada la confidencialidad de los datos contenidos en ella. Por favor dedique unos minutos a rellenarla para conocer su opinión sobre la utilización de las nuevas tecnologías en el ámbito educativo. Nos interesa también saber su opinión sobre el uso de VEDILS para la creación de actividades educativas usando dispositivos móviles. Le agradecemos de antemano su colaboración.

1. **Edad** *

____

2. **Sexo** *

Mark only one oval.

- Mujer
- Hombre

3. **Años de experiencia como docente**

____

4. **Área principal en la que has impartido docencia** *

Mark only one oval.

- Arte y Humanidades
- Ciencias
- Ciencias de la Salud
- Ciencias Sociales y Jurídicas
- Ingeniería y Arquitectura

5. **Titulación superior que posees** *

Mark only one oval.

- Doctor
- Master
- Ingeniero
- Licenciado
- Ingeniero técnico
- Diplomado

En las actividades educativas uso tecnologías como ...

6. **... dispositivos móviles.**
(1) Nunca - (2) Raramente - (3) A veces - (4) Frecuentemente - (5) Siempre
Mark only one oval.

1	2	3	4	5
⬭	⬭	⬭	⬭	⬭

7. **... cuestionarios on-line.**
(1) Nunca - (2) Raramente - (3) A veces - (4) Frecuentemente - (5) Siempre
Mark only one oval.

1	2	3	4	5
⬭	⬭	⬭	⬭	⬭

8. **... redes sociales como medio de comunicación.**
(1) Nunca - (2) Raramente - (3) A veces - (4) Frecuentemente - (5) Siempre
Mark only one oval.

1	2	3	4	5
⬭	⬭	⬭	⬭	⬭

9. **... realidad aumentada.**
(1) Nunca - (2) Raramente - (3) A veces - (4) Frecuentemente - (5) Siempre
Mark only one oval.

1	2	3	4	5
⬭	⬭	⬭	⬭	⬭

10. **...aplicaciones específicas que el alumnado debe instalarse en sus ordenadores o dispositivos móviles.**
(1) Nunca - (2) Raramente - (3) A veces - (4) Frecuentemente - (5) Siempre
Mark only one oval.

1	2	3	4	5
⬭	⬭	⬭	⬭	⬭

Tengo conocimientos tecnológicos para ...

11. **... programar actividades educativas usando lenguajes como Java, C#, Javascript, etc.**
(1) Nada - (2) Poco - (3) Algo - (4) Bastante - (5) Mucho
Mark only one oval.

1	2	3	4	5
⬭	⬭	⬭	⬭	⬭

12. **... desarrollar aplicaciones para dispositivos móviles (Android, IOS, etc…)**
(1) Nada - (2) Poco - (3) Algo - (4) Bastante - (5) Mucho
Mark only one oval.

1	2	3	4	5
⬭	⬭	⬭	⬭	⬭

13. **... realizar actividades educativas que incluyan realidad aumentada.**
(1) Nada - (2) Poco - (3) Algo - (4) Bastante - (5) Mucho
Mark only one oval.

1	2	3	4	5
⬭	⬭	⬭	⬭	⬭

14. **... desarrollar aplicaciones que utilizan algún gadget (dispositivos que detecten el movimiento, el pulso, latidos del corazón, ondas cerebrales, ...)**
(1) Nada - (2) Poco - (3) Algo - (4) Bastante - (5) Mucho
Mark only one oval.

1	2	3	4	5
⬭	⬭	⬭	⬭	⬭

FACILIDAD DE USO PERCIBIDA

Pienso que VEDILS permite de manera sencilla ...

15. **... aprender a utilizarlo.**
(1) Totalmente desacuerdo (2) En desacuerdo (3) Ni de acuerdo ni en acuerdo (4) De acuerdo (5) Totalmente de acuerdo
Mark only one oval.

1	2	3	4	5
⬭	⬭	⬭	⬭	⬭

16. **... usarse gracias a su entorno visual de desarrollo.**
(1) Totalmente desacuerdo (2) En desacuerdo (3) Ni de acuerdo ni en acuerdo (4) De acuerdo (5) Totalmente de acuerdo
Mark only one oval.

1	2	3	4	5
⬭	⬭	⬭	⬭	⬭

17. **... programar por el uso de un lenguaje visual basado en bloques.**
(1) Totalmente desacuerdo (2) En desacuerdo (3) Ni de acuerdo ni en acuerdo (4) De acuerdo (5) Totalmente de acuerdo
Mark only one oval.

1	2	3	4	5
⬭	⬭	⬭	⬭	⬭

18. **... crear actividades educativas.**
(1) Totalmente desacuerdo (2) En desacuerdo (3) Ni de acuerdo ni en acuerdo (4) De acuerdo (5) Totalmente de acuerdo
Mark only one oval.

1	2	3	4	5
⬭	⬭	⬭	⬭	⬭

19. **... crear aplicaciones con realidad aumentada.**
(1) Totalmente desacuerdo (2) En desacuerdo (3) Ni de acuerdo ni en acuerdo (4) De acuerdo (5) Totalmente de acuerdo
Mark only one oval.

1	2	3	4	5
⬭	⬭	⬭	⬭	⬭

20. **... integrar realidad aumentada con otras opciones del dispositivo móvil.**
(1) Totalmente desacuerdo (2) En desacuerdo (3) Ni de acuerdo ni en acuerdo (4) De acuerdo (5) Totalmente de acuerdo
Mark only one oval.

1	2	3	4	5
⬭	⬭	⬭	⬭	⬭

21. **... crear aplicaciones que hacen uso de gadgets (dispositivos que detecten el movimiento, el pulso, latidos del corazón, ondas cerebrales, ...).**
(1) Totalmente desacuerdo (2) En desacuerdo (3) Ni de acuerdo ni en acuerdo (4) De acuerdo (5) Totalmente de acuerdo
Mark only one oval.

1	2	3	4	5
⬭	⬭	⬭	⬭	⬭

22. **... analizar como mi alumnado ha realizado las actividades educativas en el dispositivo móvil.**
(1) Totalmente desacuerdo (2) En desacuerdo (3) Ni de acuerdo ni en acuerdo (4) De acuerdo (5) Totalmente de acuerdo
Mark only one oval.

1	2	3	4	5
⬭	⬭	⬭	⬭	⬭

UTILIDAD PERCIBIDA

La herramienta VEDILS me permite ...

23. **... desarrollar mis ideas sobre actividades educativas para dispositivos móviles.**
    (1) Totalmente desacuerdo (2) En desacuerdo (3) Ni de acuerdo ni en acuerdo (4) De acuerdo (5) Totalmente de acuerdo
    Mark only one oval.

1	2	3	4	5
⬭	⬭	⬭	⬭	⬭

24. **... crear aplicaciones sin grandes conocimientos de programación.**
    (1) Totalmente desacuerdo (2) En desacuerdo (3) Ni de acuerdo ni en acuerdo (4) De acuerdo (5) Totalmente de acuerdo
    Mark only one oval.

1	2	3	4	5
⬭	⬭	⬭	⬭	⬭

25. **... usar la realidad aumentada como elemento motivador de los alumnos.**
    (1) Totalmente desacuerdo (2) En desacuerdo (3) Ni de acuerdo ni en acuerdo (4) De acuerdo (5) Totalmente de acuerdo
    Mark only one oval.

1	2	3	4	5
⬭	⬭	⬭	⬭	⬭

26. **... usar la realidad aumentada para explicar conceptos a los alumnos.**
    (1) Totalmente desacuerdo (2) En desacuerdo (3) Ni de acuerdo ni en acuerdo (4) De acuerdo (5) Totalmente de acuerdo
    Mark only one oval.

1	2	3	4	5
⬭	⬭	⬭	⬭	⬭

27. **... crear actividades educativas que hagan uso de gadgets.**
    (1) Totalmente desacuerdo (2) En desacuerdo (3) Ni de acuerdo ni en acuerdo (4) De acuerdo (5) Totalmente de acuerdo
    Mark only one oval.

1	2	3	4	5
⬭	⬭	⬭	⬭	⬭

28. **... analizar las actividades educativas.**
(1) Totalmente desacuerdo (2) En desacuerdo (3) Ni de acuerdo ni en acuerdo (4) De acuerdo (5) Totalmente de acuerdo
Mark only one oval.

1	2	3	4	5
⬭	⬭	⬭	⬭	⬭

ACTITUD HACIA EL USO

Creo que VEDILS ...

29. **... es interesante para crear aplicaciones para dispositivos móviles con Realidad Aumentada**
(1) Totalmente desacuerdo (2) En desacuerdo (3) Ni de acuerdo ni en acuerdo (4) De acuerdo (5) Totalmente de acuerdo
Mark only one oval.

1	2	3	4	5
⬭	⬭	⬭	⬭	⬭

30. **... es interesante para realizar analítica del aprendizaje en dispositivos móviles**
(1) Totalmente desacuerdo (2) En desacuerdo (3) Ni de acuerdo ni en acuerdo (4) De acuerdo (5) Totalmente de acuerdo
Mark only one oval.

1	2	3	4	5
⬭	⬭	⬭	⬭	⬭

31. **... aporta los elementos necesarios para desarrollar actividades de aprendizaje.**
(1) Totalmente desacuerdo (2) En desacuerdo (3) Ni de acuerdo ni en acuerdo (4) De acuerdo (5) Totalmente de acuerdo
Mark only one oval.

1	2	3	4	5
⬭	⬭	⬭	⬭	⬭

32. **... será una de las herramientas que utilizaré para desarrollar mis actividades educativas.**
(1) Totalmente desacuerdo (2) En desacuerdo (3) Ni de acuerdo ni en acuerdo (4) De acuerdo (5) Totalmente de acuerdo
Mark only one oval.

1	2	3	4	5
⬭	⬭	⬭	⬭	⬭

Powered by

Google Forms
