# Supplementary material for: Block-Based Development of Mobile Learning Experiences for the Internet of Things
Source: Sensors (Basel). 2019 Dec 11;19(24):5467. doi: 10.3390/s19245467 (PMC6960931; doi:10.3390/s19245467)
Supplement: Supplementary file 1 [file sensors-19-05467-s001.zip › Study with students/Slides (in spanish).pdf]

Desarrollo sencillo de apps móviles para **IoT**

IES NTRA. SRA. DE LOS REMEDIOS

José Miguel Mota Macías josemiguel.mota@uca.es
Iván Ruiz Rube ivan.ruiz@uca.es
Juan Manuel Dodero Beardo juanma.dodero@uca.es

UCA Universidad de Cádiz
Escuela Superior de Ingeniería
SPI&FM Software Process Improvement and Formal Methods group

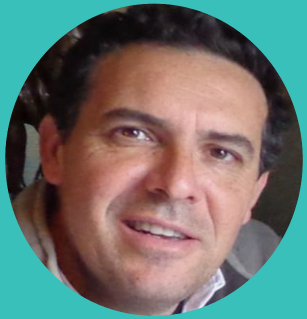

Hola!

JOSÉ MIGUEL MOTA MACÍAS

Profesor del *Dpto. de Ingeniería Informática*
Investigador en *Technology-Enhanced Learning*, mobile learning, realidad aumentada, real y mixta

@jmiguelmota

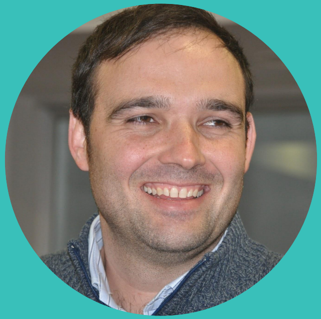

Hola!

IVÁN RUIZ RUBE

Profesor del *Dpto. de Ingeniería Informática*
Investigador en *Software Process Improvement, Linked Open Data* y *Technology-Enhanced Learning*

@iruizrube

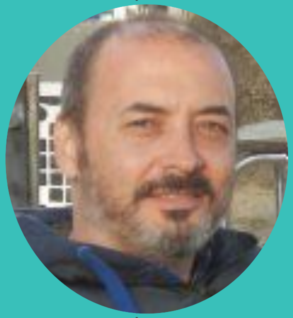

Hola!

JUAN MANUEL DODERO BEARDO

Catedrático del *Dpto. de Ingeniería Informática*
Investigador en *Technology-Enhanced Learning*, Computational thinking e Internet Computing

@jmdodero

Índice

- Internet de las Cosas
- IoT y la educación
- Desarrollo sencillo de apps Android para IoT
- Ejemplo: una app móvil para medición de la temperatura

1

Internet de las cosas

Internet of Things (IoT)

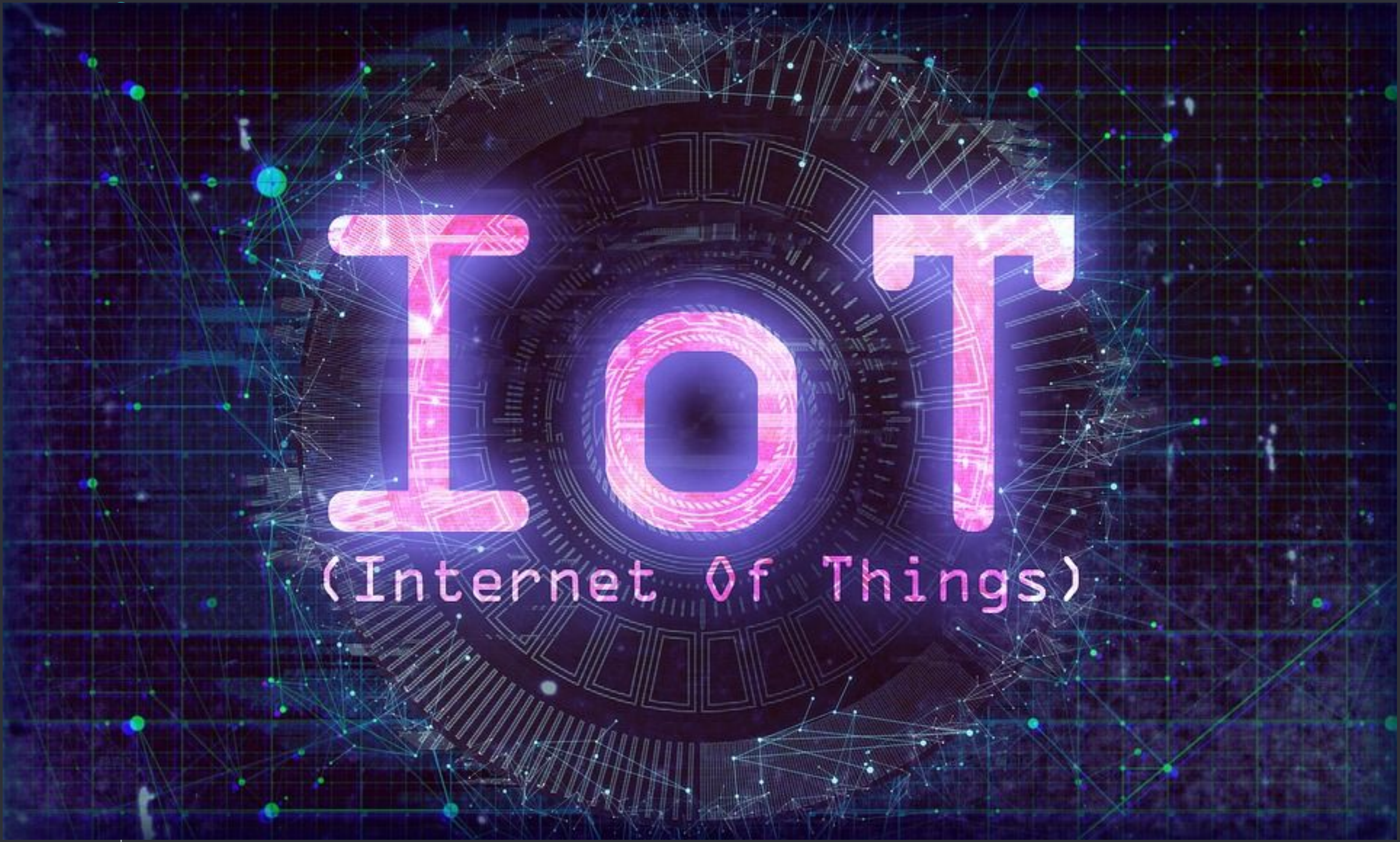

Image by methodshop from Pixabay

Internet de las Cosas

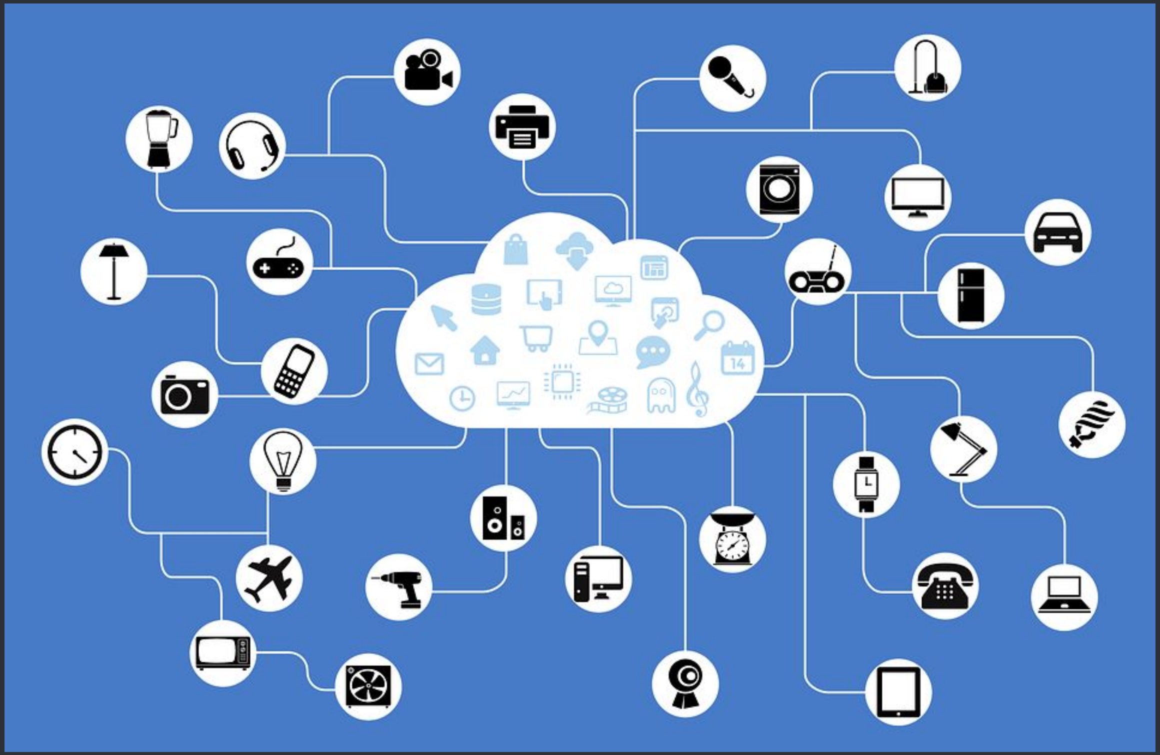

Image by jeferrb from Pixabay

Conjunto de dispositivos **mecánicos, electrónicos u objetos** de cualquier tipología **conectados en red**.

Aplicaciones

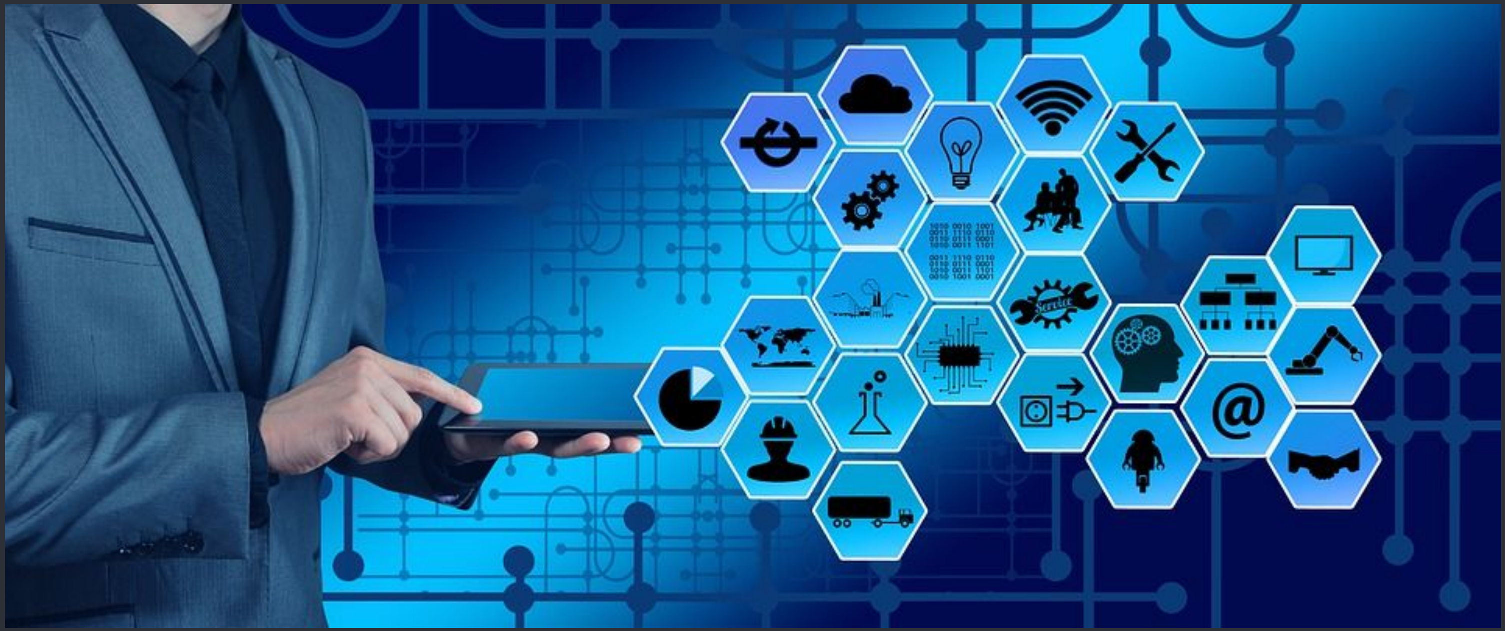

Image by Gerd Altmann from Pixabay

Aplicaciones en **múltiples sectores**:

Smart homes, Smart cities, Smart industries, Transportation, Internet of Health things, Internet of People, Smart Farming, Retail, Education, Automotive, etc.

Elementos del ecosistema IoT

- **Sensores**
  - temperatura, presión, luz, humedad, gas, óptico, etc.
- **Actuadores**
  - servomotores, válvulas, etc.
- **Periféricos**:
  - luminosos LED, pantallas táctiles, cámaras, etc.
- **Protocolos de comunicación/ middleware:**
  - transporte: WiFi, Bluetooth, LPWAN, etc.
  - datos: MQTT, Websockets, ReST, etc.

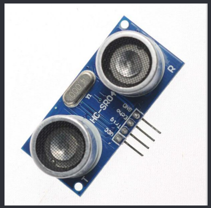

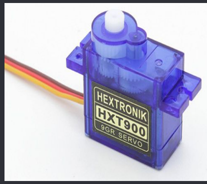

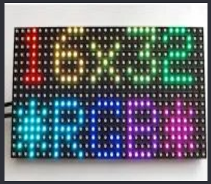

Internet de las Cosas

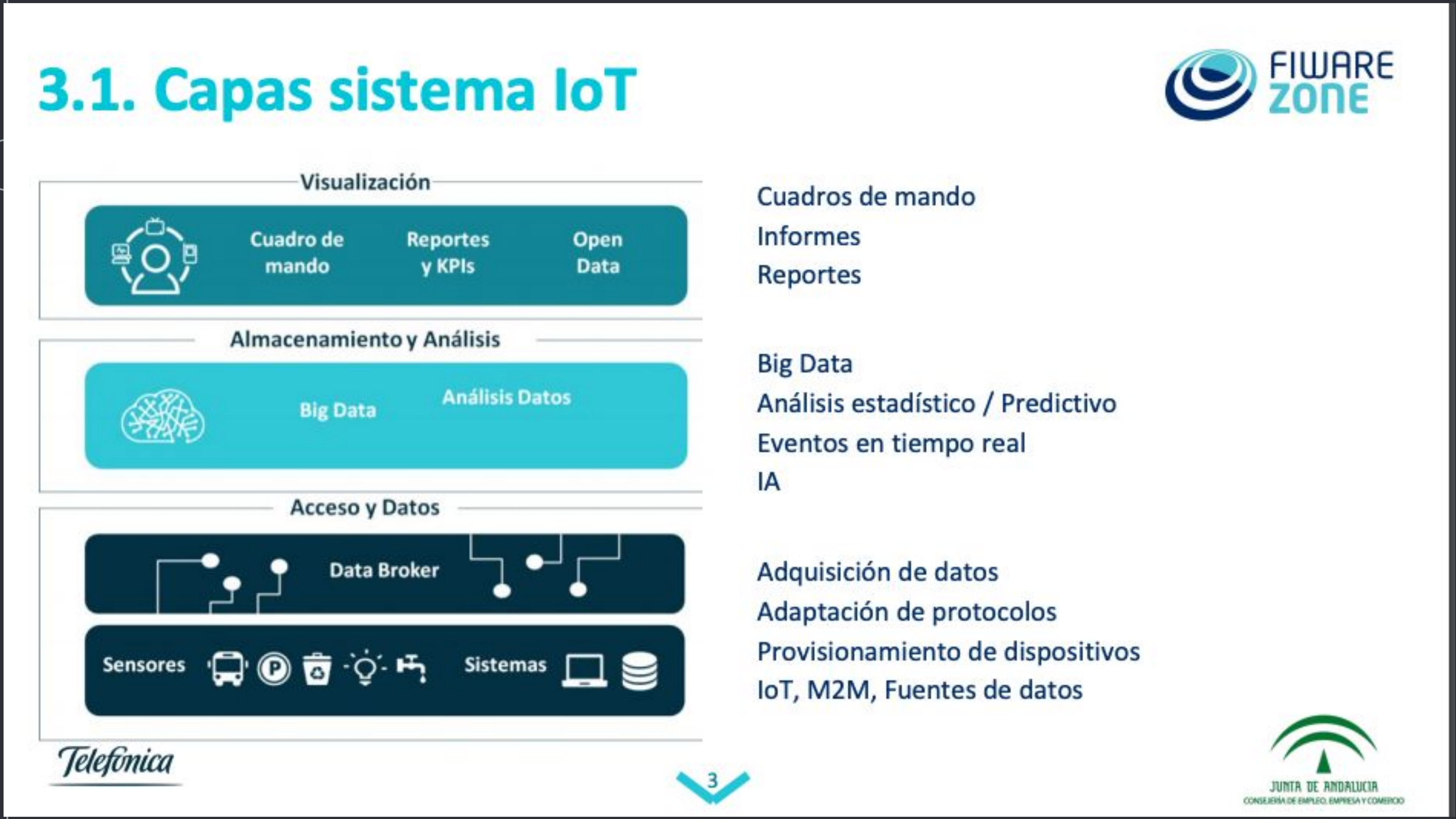

FIWARE es una **plataforma**, impulsada por la UE, **para el despliegue de aplicaciones de ioT**. Define una arquitectura y unas especificaciones para que los desarrolladores y organizaciones desarrollen productos que satisfagan sus necesidades

2 IoT y la educación

Arduino

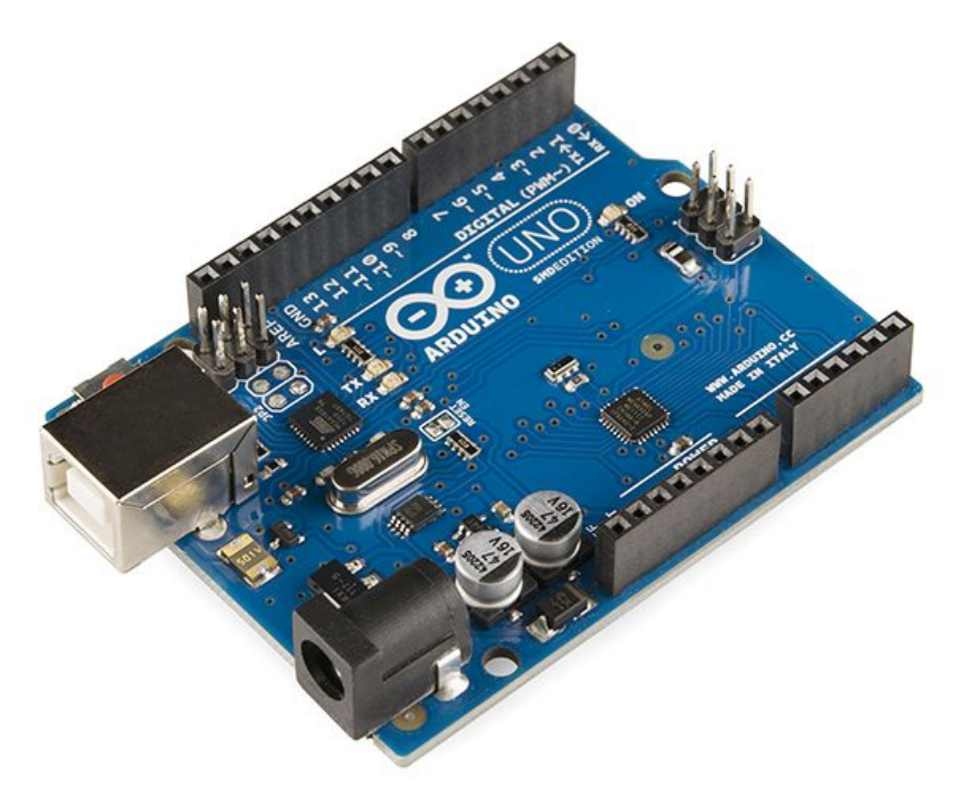

Plataforma **abierta** para la programación de microcontroladores

Aprendiendo a programar

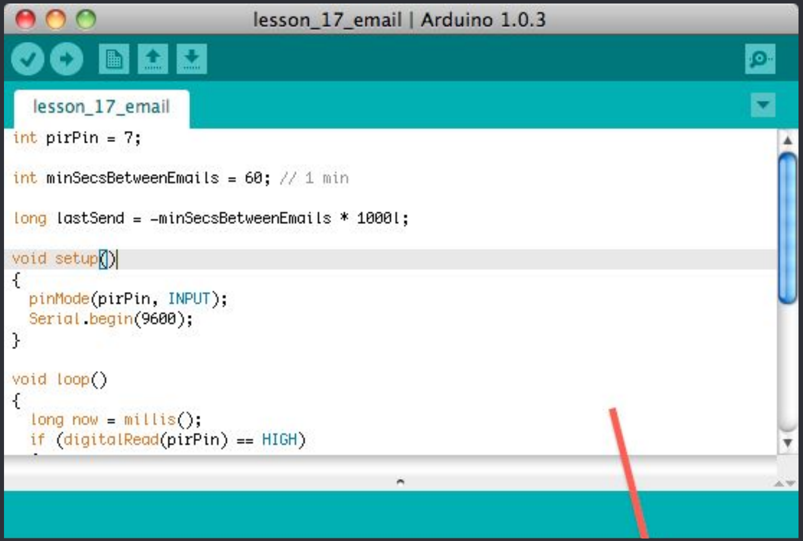

Herramientas **textuales** (C / Python)

Herramientas **visuales** (Scratch)

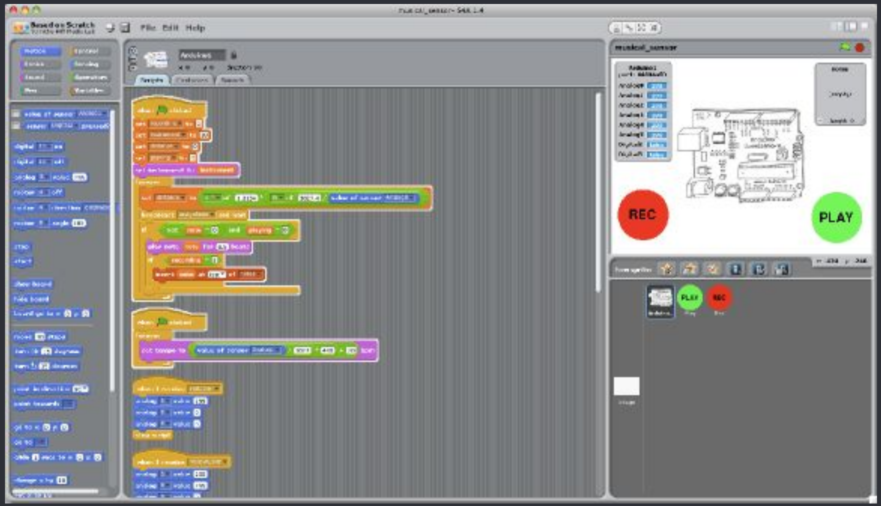

Raspberry

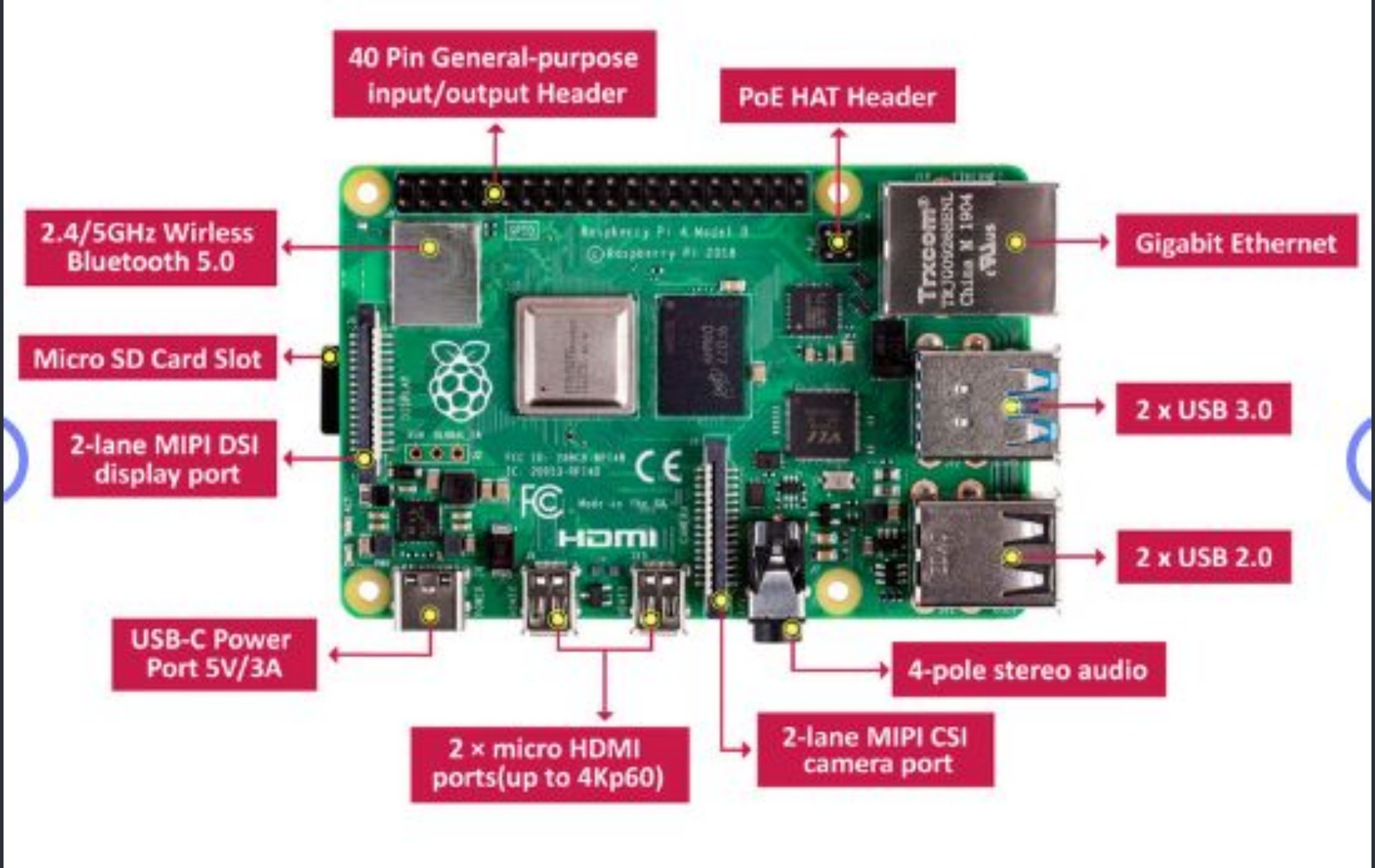

Mini-ordenador: *Single-board computer*

Raspberry

- Desarrollado en Reino Unido por la *Fundación Raspberry Pi*
- Objetivo: estimular la enseñanza de **ciencias de la computación** en las escuelas.
- Se trata de un ordenador de **bajo coste,** sin fuente de alimentación, ni carcasa, ni dispositivo de almacenamiento
- Gran cantidad de periféricos y dispositivos **conectables**

General-Purpose Input/Output (GPIO)

- Pins programables de entrada o salida:
  - Valor alto (3.3V)
  - Valor bajo (0V)

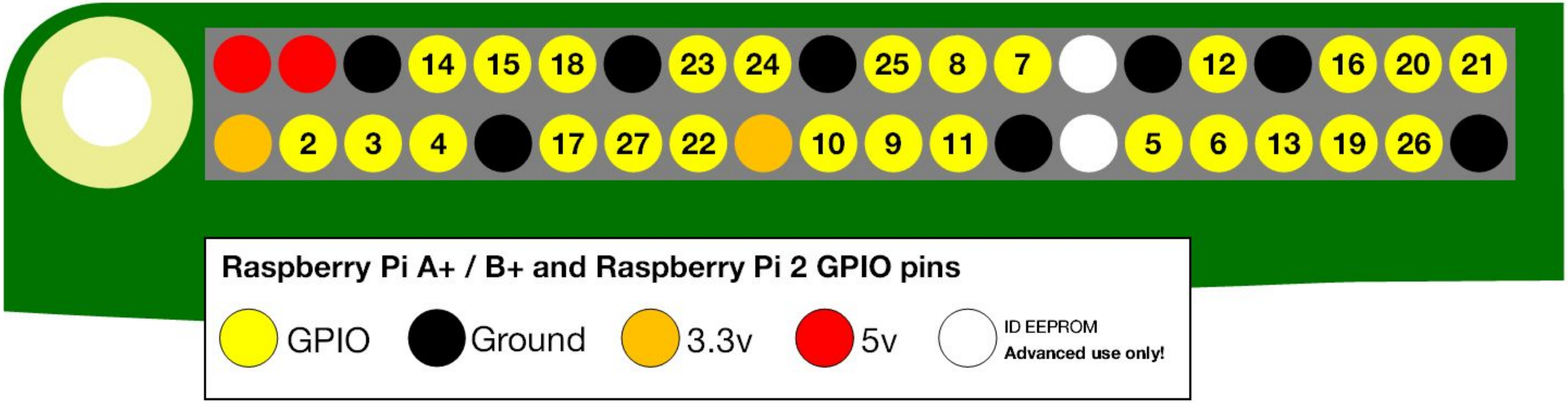

Aplicaciones

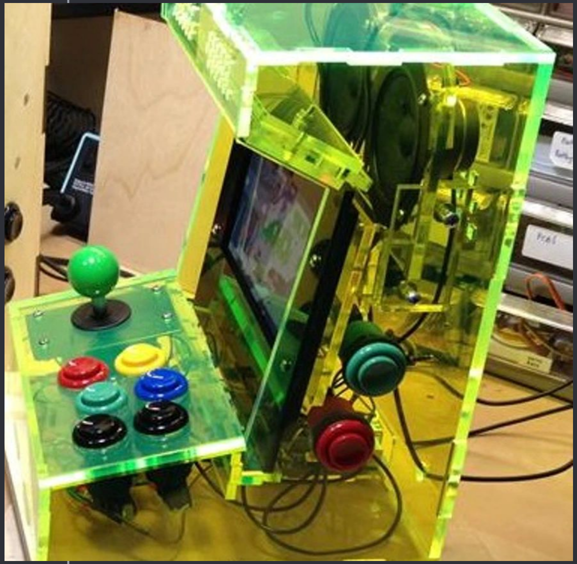

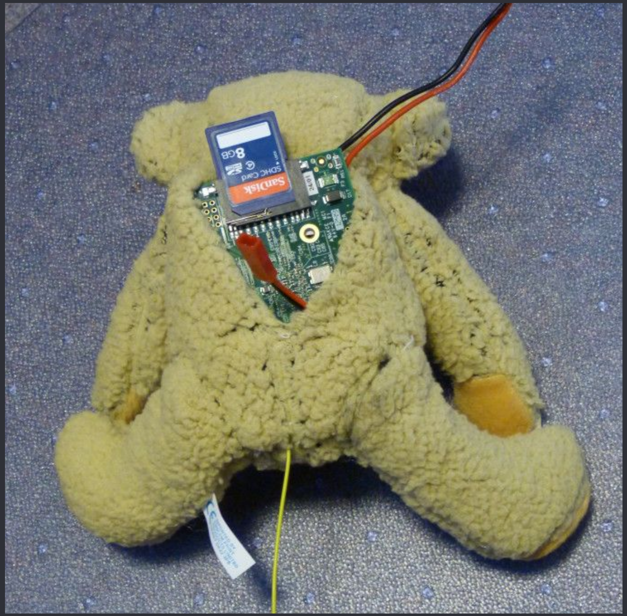

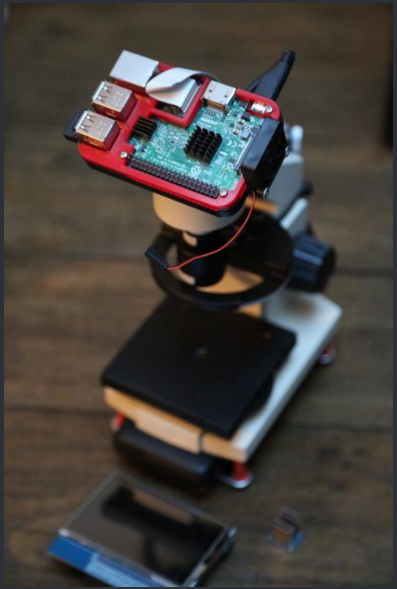

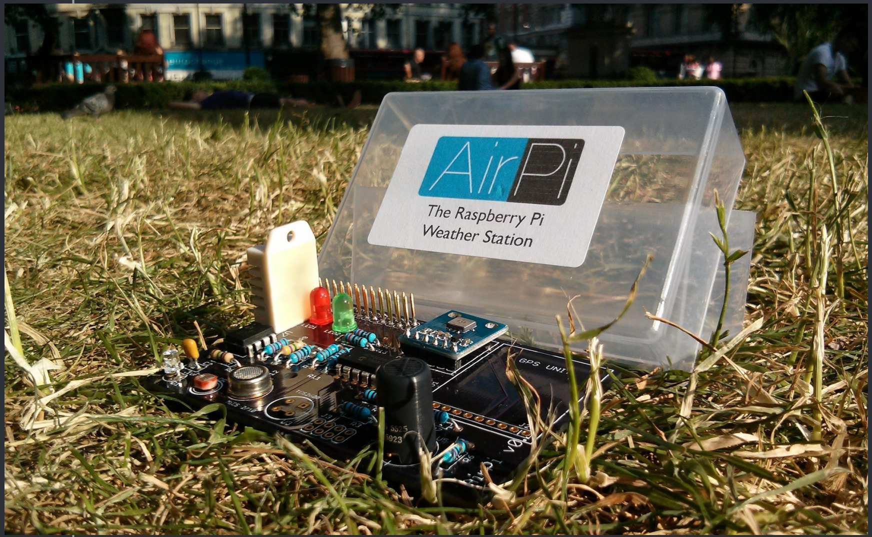

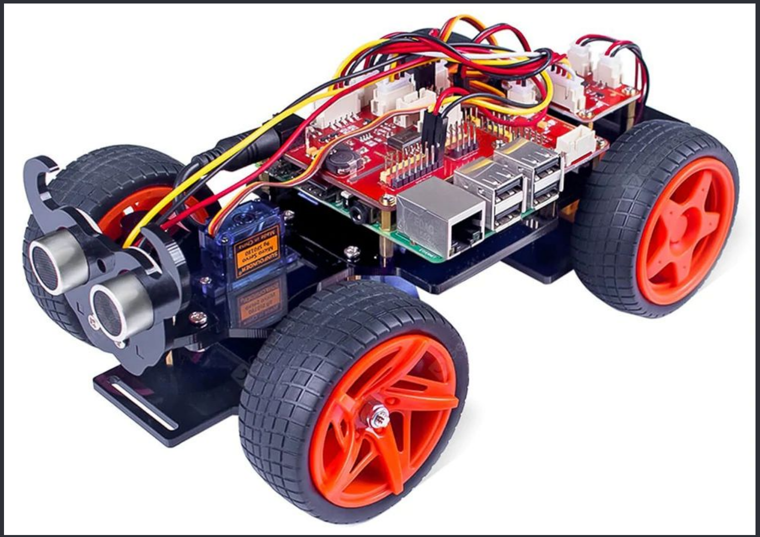

Sense HAT

- 8x8 RGB LED Matrix
- Joystick 5 botones
- Sensores ambientales
  - Giróscopo
  - Acelerómetro
  - Magnetómetro
  - Temperatura
  - Barómetro
  - Humedad
- API en Python

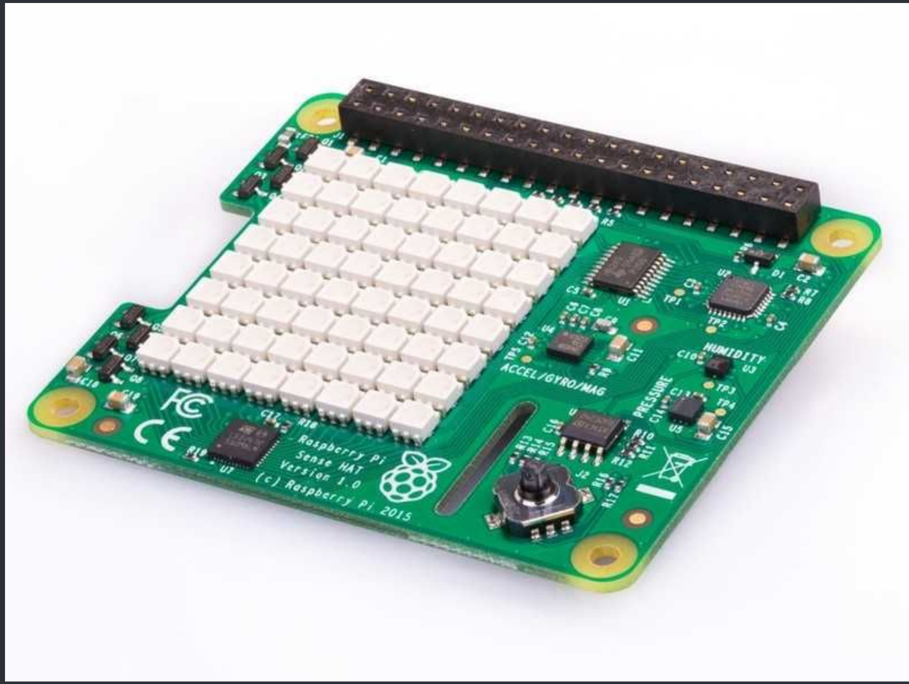

Astro PI

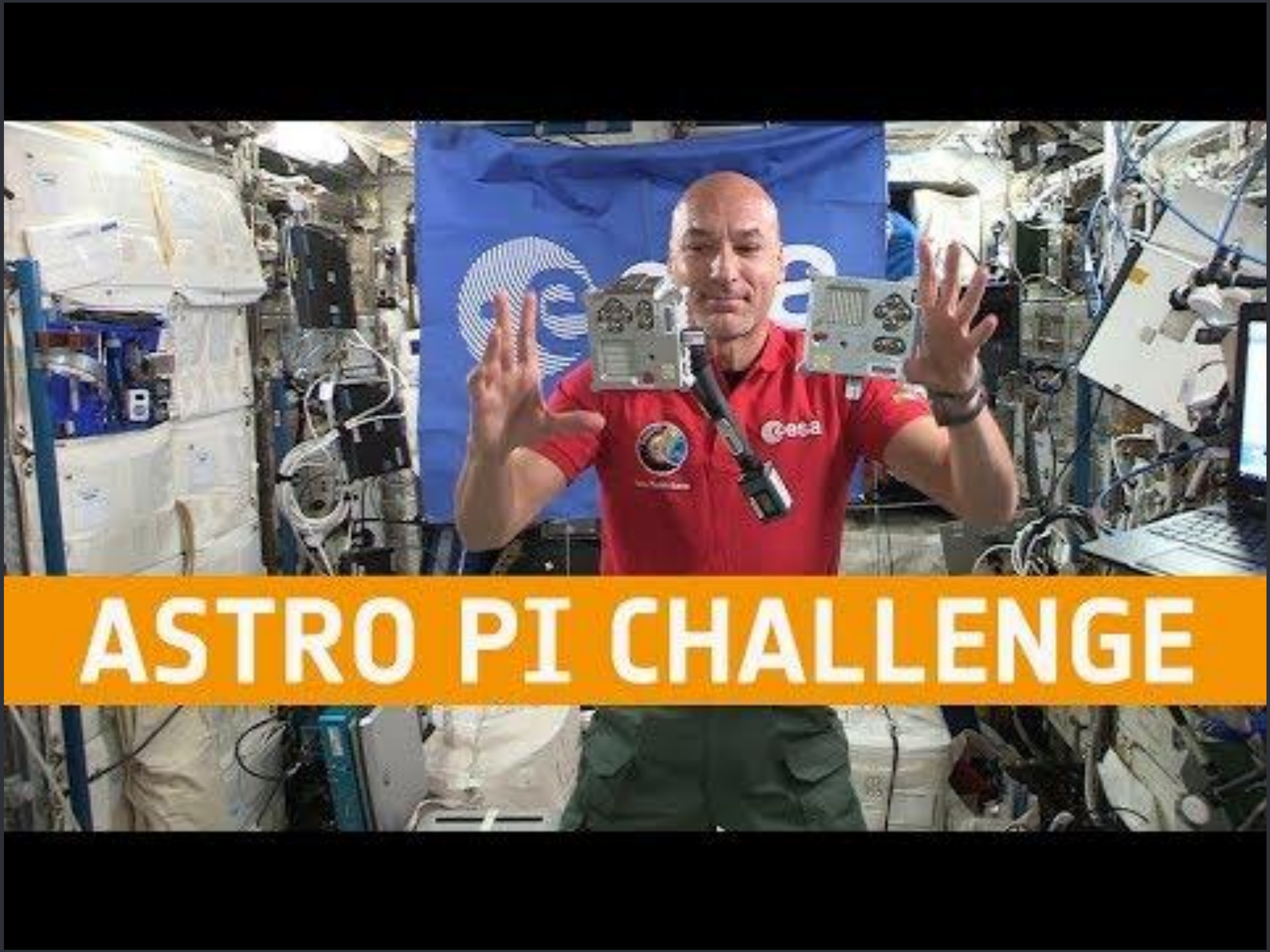

3

Desarrollo sencillo de apps Android para IoT

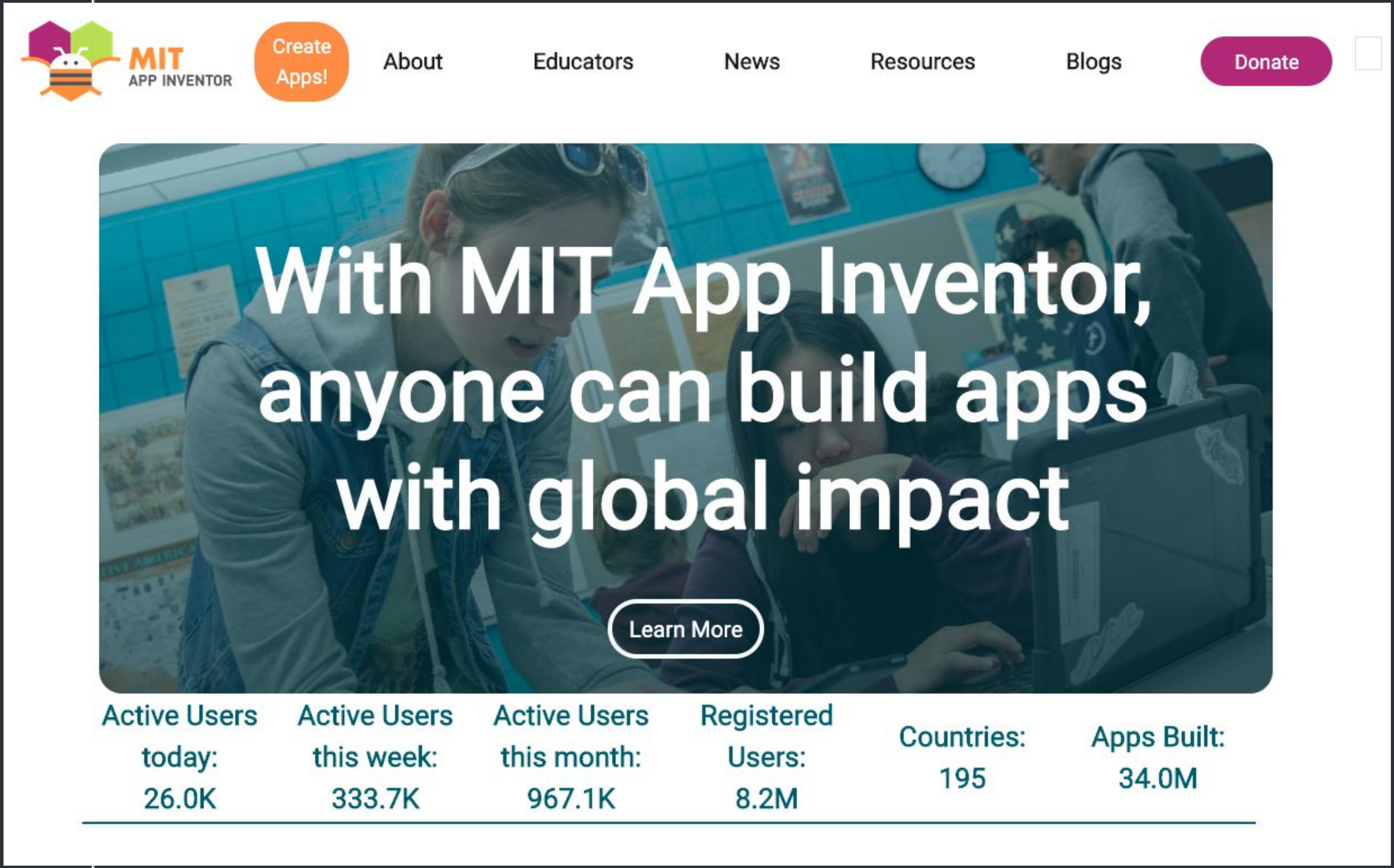
MIT APP INVENTOR
Create Apps!
About
Educators
News
Resources
Blogs
Donate
With MIT App Inventor, anyone can build apps with global impact
Learn More
Active Users today: 26.0K
Active Users this week: 333.7K
Active Users this month: 967.1K
Registered Users: 8.2M
Countries: 195
Apps Built: 34.0M

Extensiones para AppInventor

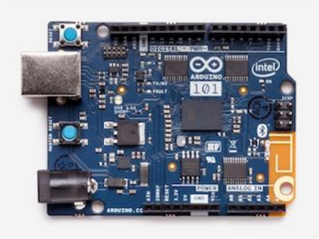

Arduino 101
The Arduino 101 is a version of the popular Arduino platform based on the Intel® Curie™ chipset. It has the same form factor as many other Arduino but supports built-in Bluetooth® low energy.

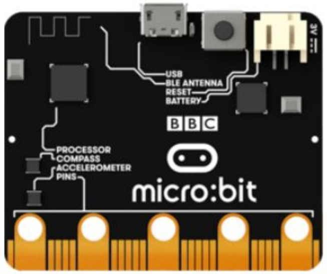

BBC micro:bit
The micro:bit is a computing platform from the BBC. It is an open platform for developing all manner of projects and is programmable by many different editors, including a blocks editor provided by Microsoft. Learn more about the micro:bit at the Micro:bit Educational Foundation's website.

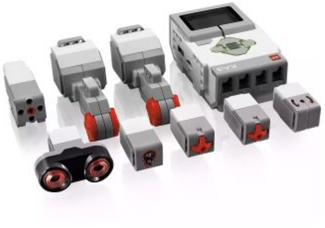

Lego EV3 robot
Lego Mindstorms EV3 is the third generation robotics kit in Lego's Mindstorms product line. Lego EV3 can interact with App Inventor through Bluetooth and Wi-Fi(need additional Wi-Fi dongle).

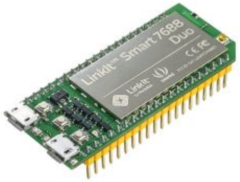

LinkIt Smart 7688/7688 Duo
The LinkIt Smart 7688 Duo development board uses MT7688AN as its MPU, complemented by an ATmega32U4 MCU. In addition to application development in Python, Node.js and native C for MT7688AN, applications for the MCU can be developed using the Arduino IDE. LinkIt Smart 7688/7688 Duo can interact with App Inventor through Wi-Fi.

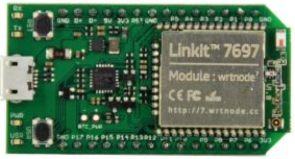

LinkIt 7697
LinkIt 7697 is an Arduino-compatible dev board of the LinkIt IoT platform with onboard Bluetooth Low energy and W-fi ommunication. It provides hardware development kits (HDKs) for user to develop prototypes of IoT devices. LinkIt 7697 can interact with App Inventor through Bluetooth Low energy and Wi-Fi.

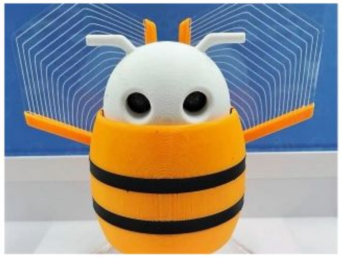

MIT App Inventor Codi Bot
The MIT App Inventor Codi Bot is a hands-on IoT kit. Our adorable mascot can be controlled via App Inventor through Bluetooth communication. This educational kit is easy to use and guides users through the whole process of development, from building a robot to programming it.

http://iot.appinventor.mit.edu

App Inventor es **software libre**

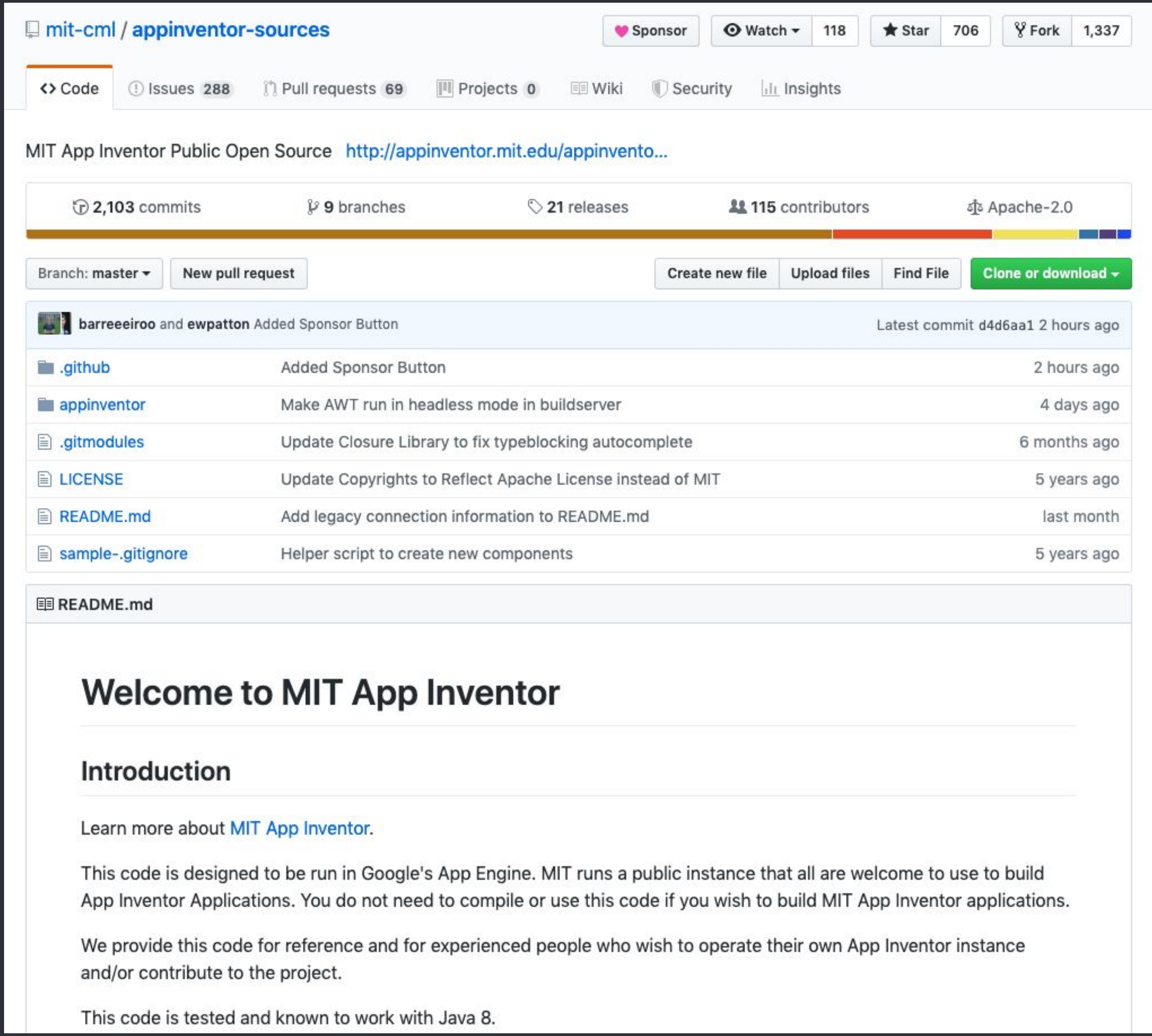

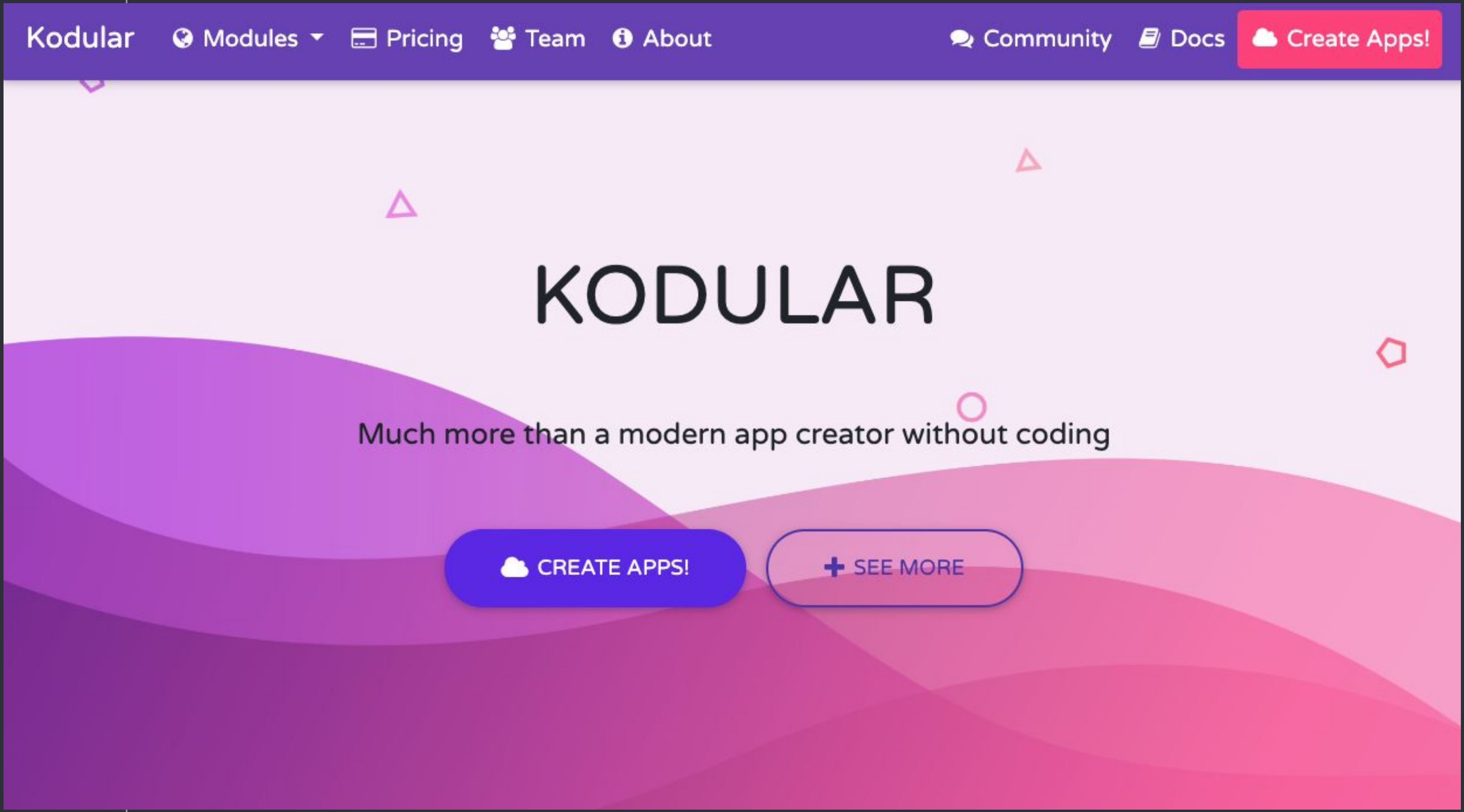
Kodular
Modules
Pricing
Team
About
Community
Docs
Create Apps!
KODULAR
Much more than a modern app creator without coding
CREATE APPS!
SEE MORE

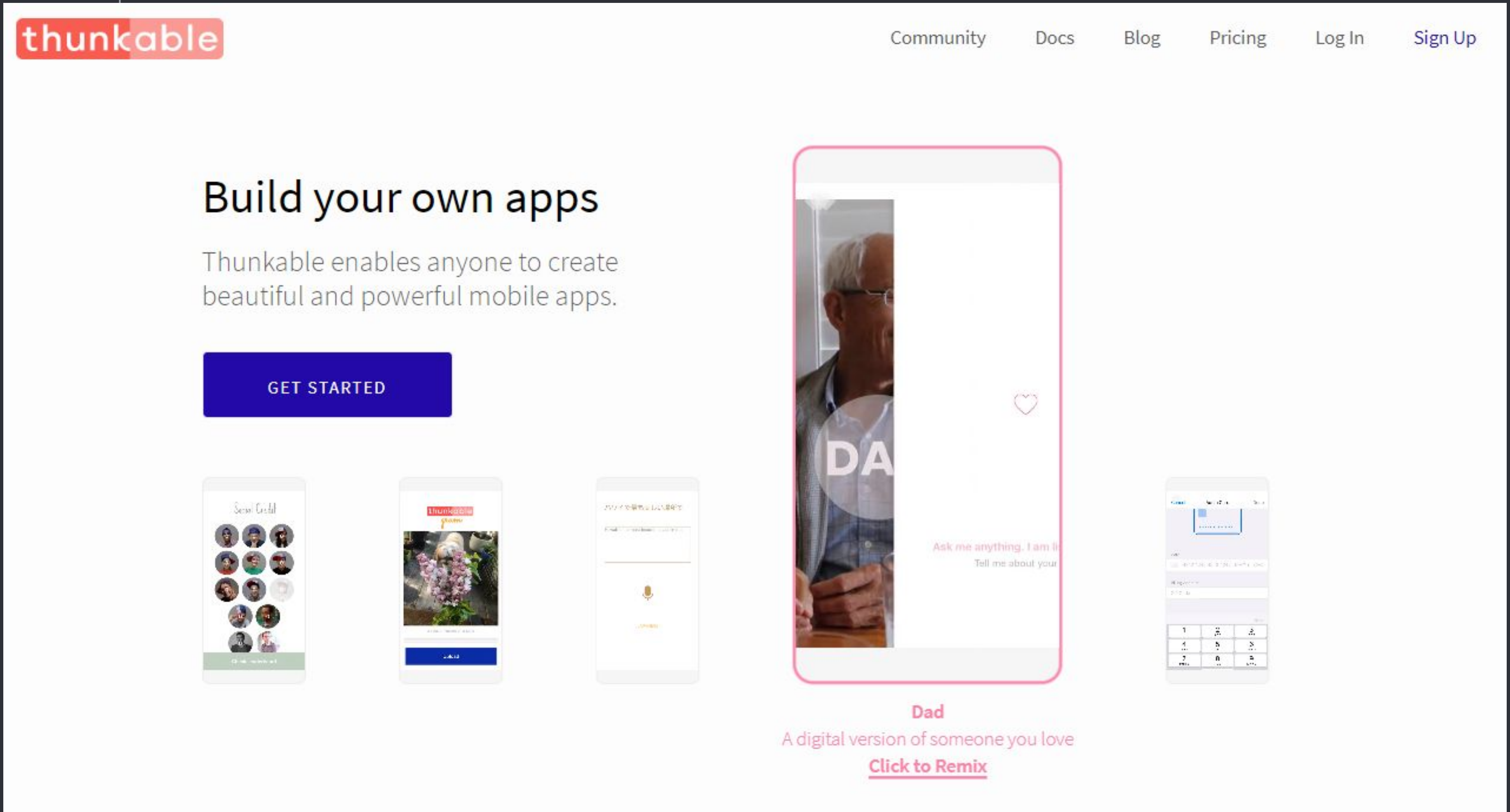
thunkable
Community
Docs
Blog
Pricing
Log In
Sign Up
Build your own apps
Thunkable enables anyone to create beautiful and powerful mobile apps.
GET STARTED
Dad
A digital version of someone you love
Click to Remix

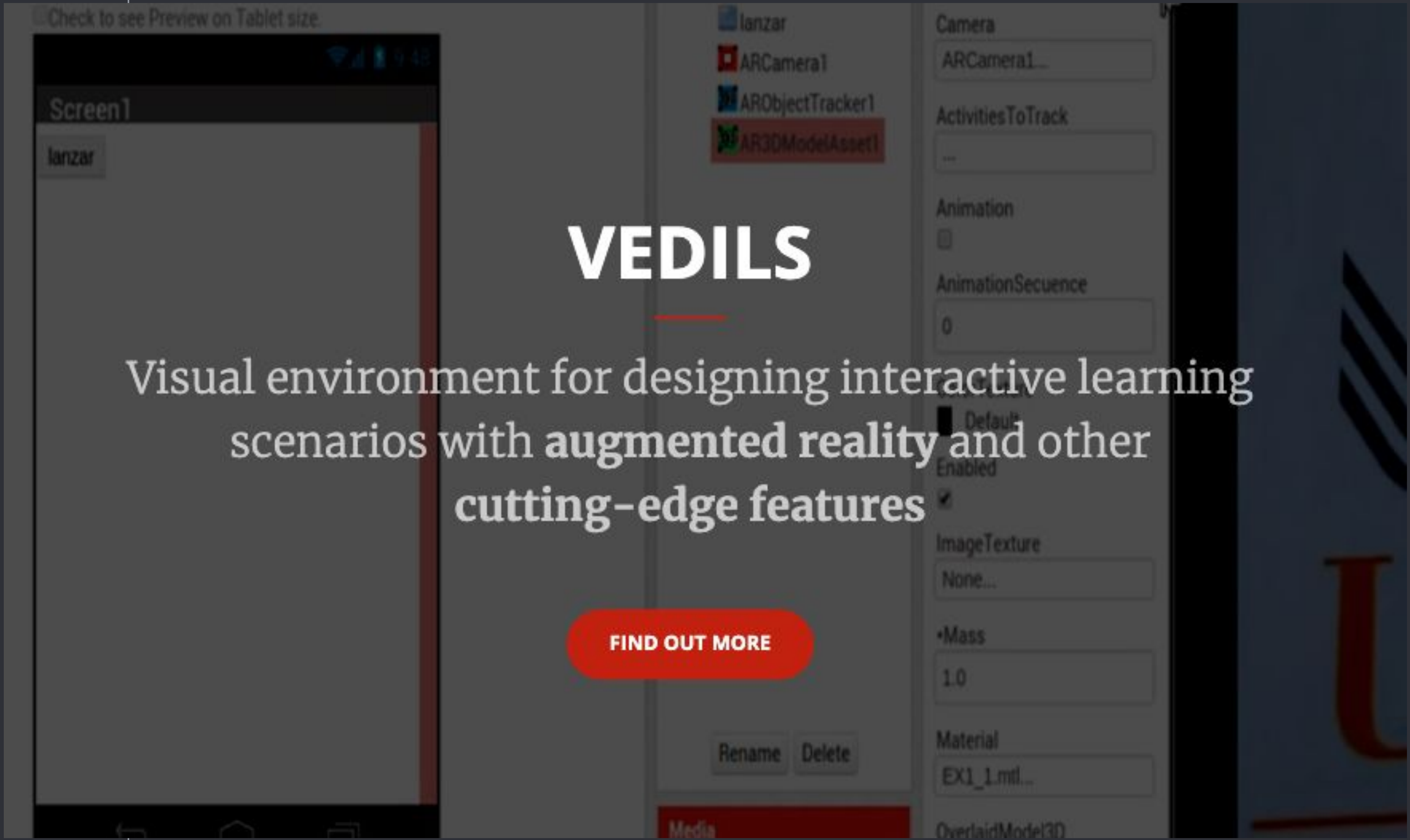
VEDILS
Visual environment for designing interactive learning scenarios with **augmented reality** and other **cutting-edge features**
FIND OUT MORE

Flujo de trabajo

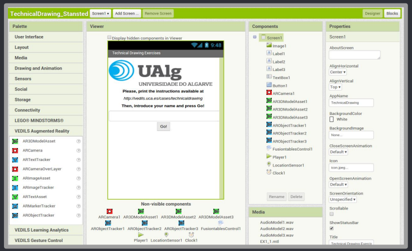

Diseño de la interfaz de usuario

En un **navegador WEB** diseñamos y configuramos los elementos del interfaz de usuario.

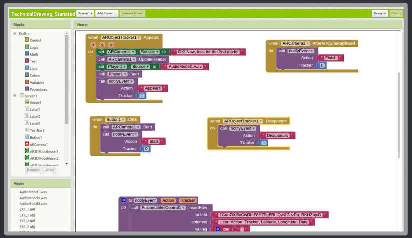

Diseño del comportamiento

Usando un **lenguaje visual de programación** definimos la lógica de la aplicación.

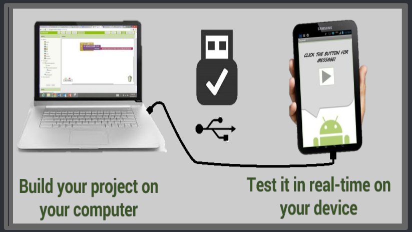

Pruebas de la aplicación

Podemos realizar pruebas en **tiempo real** de nuestra aplicación, para comprobar su comportamiento.

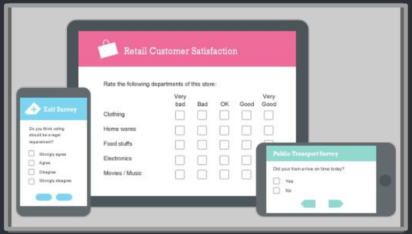

Despliegue en un dispositivo

Generamos un fichero **APK**, que son las aplicaciones que se instalan en el dispositivo Android.

4

Ejemplo: una app móvil para medición de la temperatura

Explicación del app

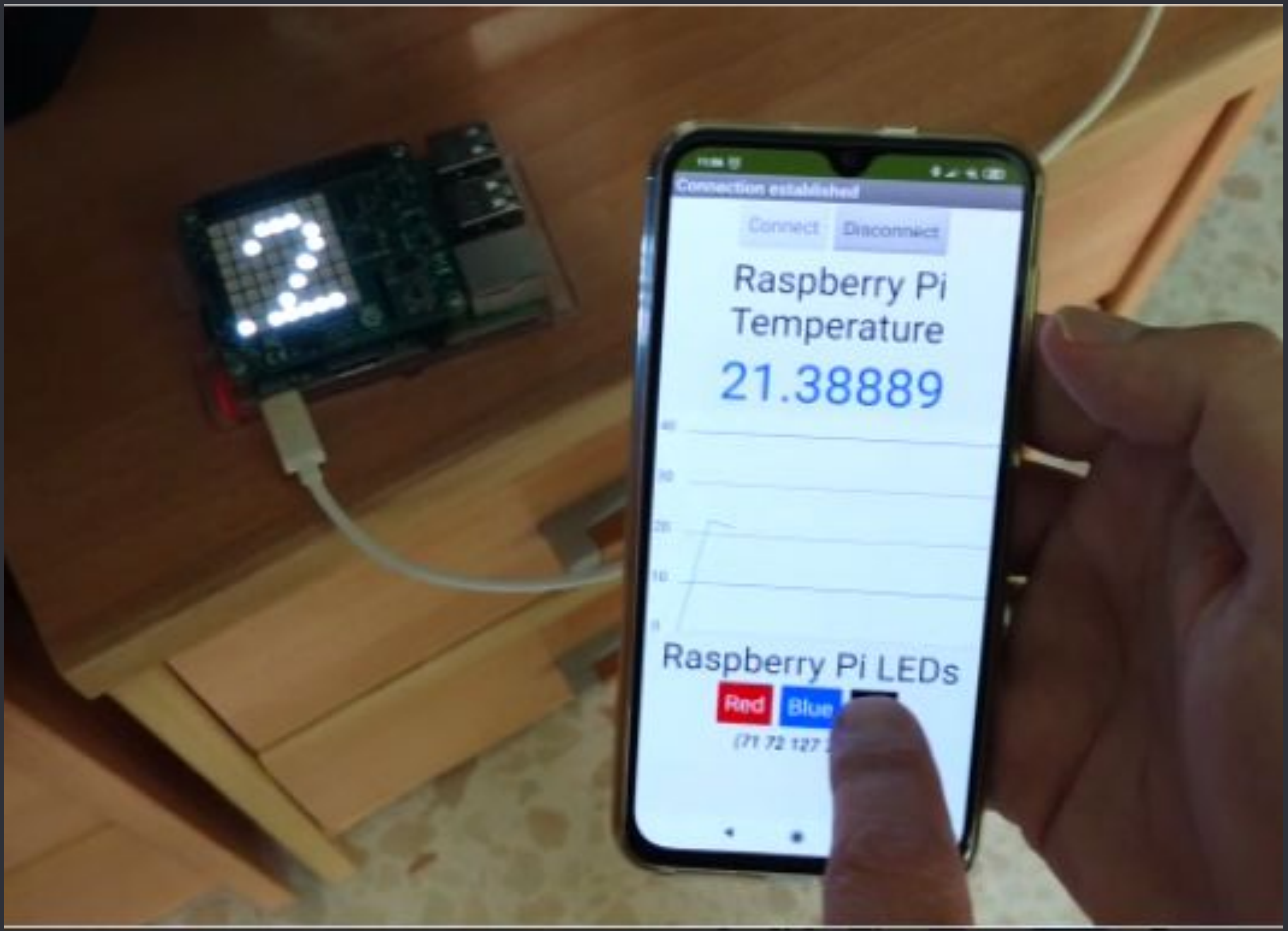

Configuración del servidor Bluetooth (Raspberry Pi)

```
server = bluetooth.BluetoothSocket(bluetooth.RFCOMM)
server.bind(("", 1))
server.listen(1)
client, address = server.accept()

## Preparar Sensehat
mysensehat = SenseHat()
text_colour = [255, 255, 255]
back_colour = [0, 0, 0]

while True:
    ## Enviamos temperatura
    sendTemperature(temperature_error)

    ## Recibimos datos desde el cliente
    data = client.recv(1024)
    if data == "r":       ## Encendemos LEDs Rojos
        colour = [255, 0, 0]
    elif data == "b":    ## Encendemos LEDs Azules
        colour = [0, 0, 255]
    elif data == "o":     ## Apagamos LEDs
        colour = [0, 0, 0]
....
```

Configuración del servidor Bluetooth (Raspberry Pi)

```
....
client_socket.close()
server_socket.close()



## Función que envía la temperatura por bluetooth
def sendTemperature(temp):
    mysensehat.clear()
    client.send(temp)
    mysensehat.show_message(temp, scroll_speed=0.025, text_colour = text_colour, back_colour = back_colour)
```

Configuración de la app móvil

Diseño de la interfaz de usuario

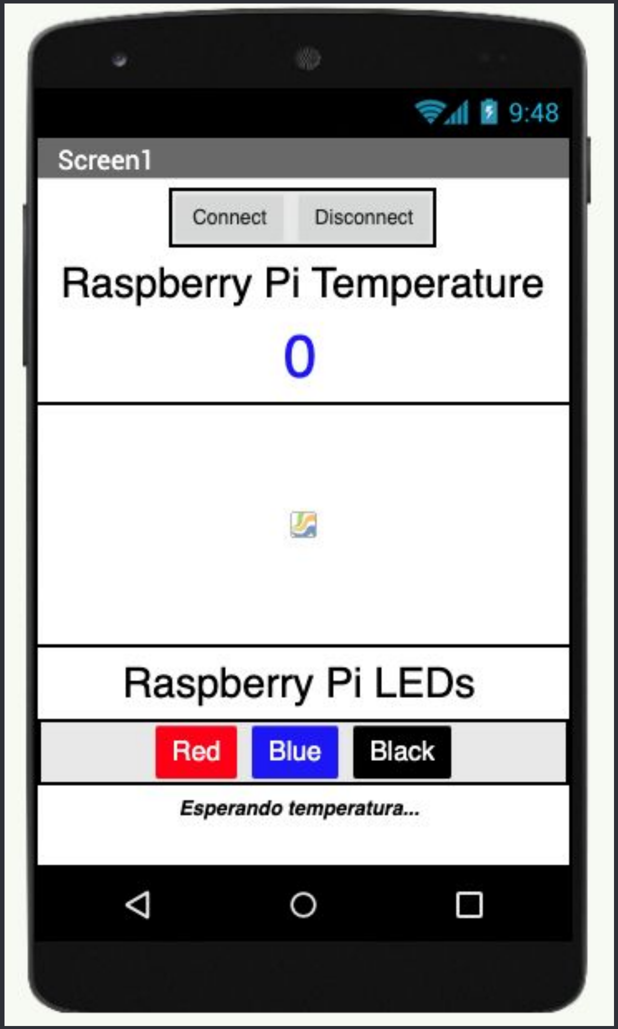

Requisitos del app

- Comprobar disponibilidad de Bluetooth
- Conectar/desconectar a dispositivo
- Enviar señales a la Raspberry Pi para que cambie el color de fondo de la matriz LED.
- Mostrar la media aritmética de la temperatura* obtenida por el sensor durante los X segundos anteriores.
- Representar gráficamente la temperatura medida por SenseHAT a lo largo del tiempo.

* El sensor de temperatura ofrece valores en grados fahrenheit entre 14 °F (-10 °C) y 104 °F (40 °C). Sin embargo, se generan esporádicamente valores anómalos de 127 °F (52.77 °C)

Programación del app

Comprobar disponibilidad de Bluetooth

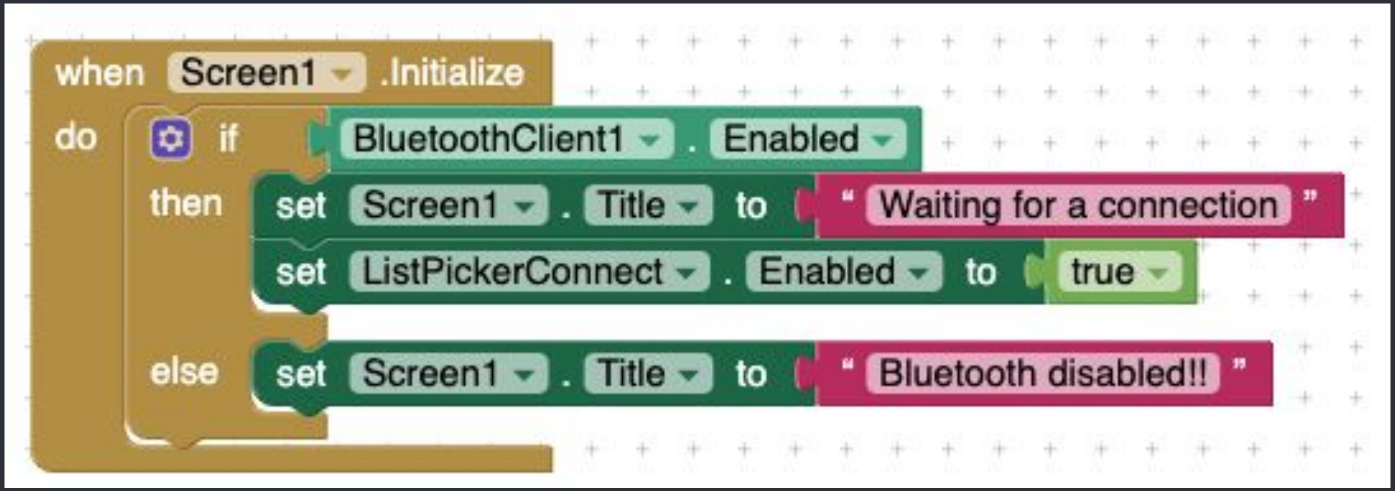

Programación del app

Conectar a dispositivo

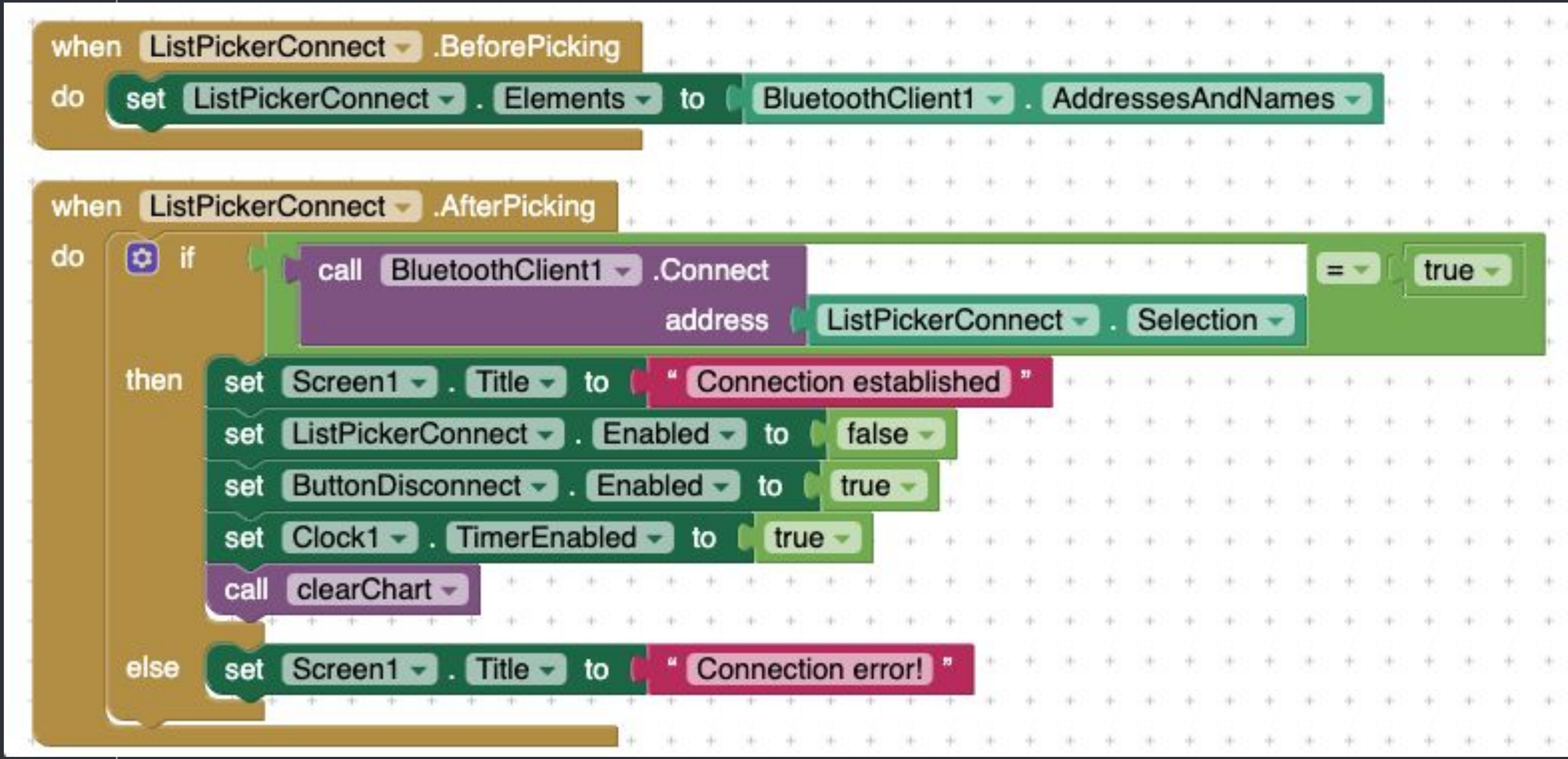

Programación del app

Desconectar del dispositivo

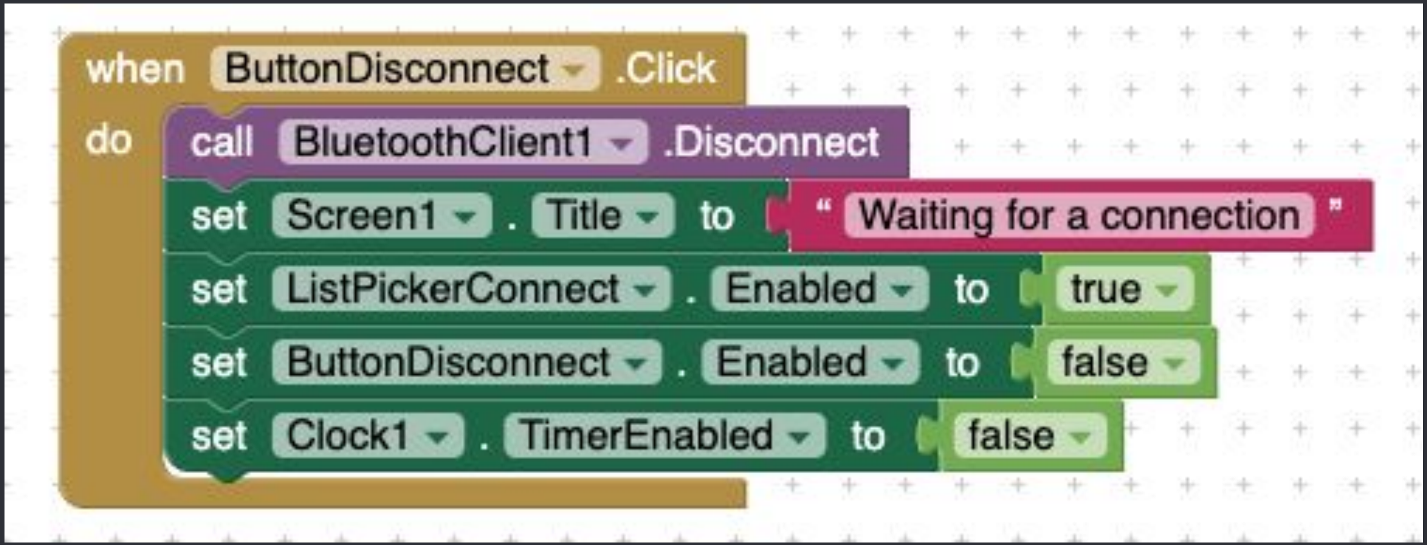

Programación del app

Enviar señales a la Raspberry Pi para que cambie el color de fondo de la matriz LED.

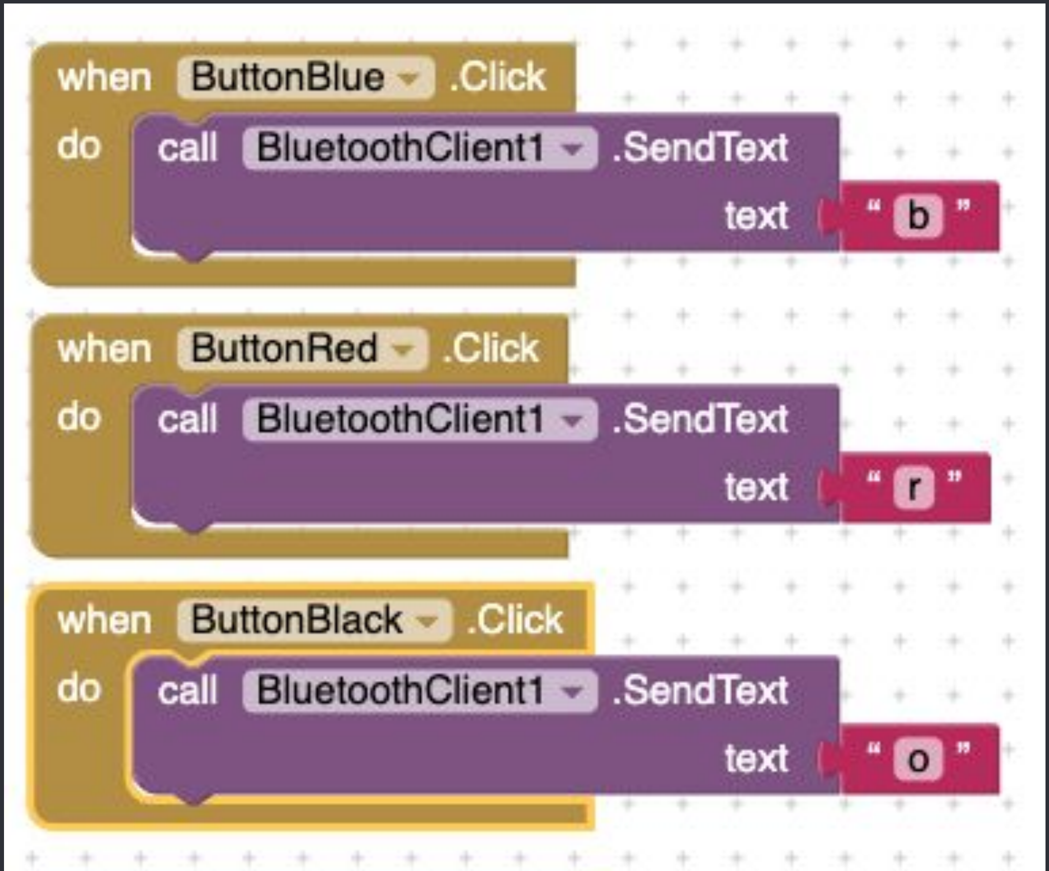

Mostrar por pantalla la media aritmética de la temperatura* obtenida por el sensor durante los X segundos anteriores.

Consideraciones:

- El sensor de temperatura ofrece valores en grados fahrenheit entre 14 °F (-10 °C) y 104 °F (40 °C).
- Se generan esporádicamente valores anómalos de 127 °F (52.77 °C)

Tutorial **App Inventor**

http://ior.ad/6Ji4

Tutorial **VEDILS**

http://ior.ad/6Jhd

Programación del app

Representar gráficamente la temperatura medida por SenseHAT a lo largo del tiempo.

Tutorial
App Inventor

http://ior.ad/6Jpu

Tutorial
VEDILS

http://ior.ad/6JtL

Encuesta final

Participa en la encuesta final:

https://tinyurl.com/y2pqer7w

Dispositivos Bluetooth

- raspberrypiJM
- Movil de Ivan
- XT1032
- Móvil de Miguel
- Móviles de hijos (x3)
- Tableta Samsung

Fuentes de los programas

- VEDILS
  - Programa completo
    https://drive.google.com/file/d/10sdq81h6wVLM19o5J7lSm_8oF6FHdWOR/view?usp=sharing
  - Programa sin código
    https://drive.google.com/file/d/17Wjq5MzQX5qUnMOFiiAdQNG4sXnXf5B1/view?usp=sharing
  - Programa sin gráficas
    https://drive.google.com/file/d/1o9f6MrmuvLWXEVJGUQYQNbHCHFrguMuM/view?usp=sharing
- AppInventor
  - Programa completo
    https://drive.google.com/file/d/1kPZEUg107DbERYXOCEUkv2tCR3dQLPx7/view?usp=sharing
  - Programa sin código
    https://drive.google.com/file/d/1Bzk-1WLskBdITrzovtBpoOnQBpM78yOW/view?usp=sharing
  - Programa sin gráficas
    https://drive.google.com/file/d/1DPueA69f0vQ9W1g4Bt7QLk6Byta54wac/view?usp=sharing
- Programa Raspberry PI
  - https://drive.google.com/file/d/1QgLw0jlRLurlt3efZj9Mkmj82cPTptAf/view?usp=sharing
- Programa servidor
  - https://drive.google.com/file/d/1c0zLt2NCFjf7_xSFh9Ta6_7JnBkO-Nts/view?usp=sharing

gracias

José Miguel Mota

josemiguel.mota@uca.es

Iván Ruiz

ivan.ruiz@uca.es

Juan Manuel Dodero

juanma.dodero@uca.es
