# Supplementary material for: Block-Based Development of Mobile Learning Experiences for the Internet of Things
Source: Sensors (Basel). 2019 Dec 11;19(24):5467. doi: 10.3390/s19245467 (PMC6960931; doi:10.3390/s19245467)
Supplement: Supplementary file 1 [file sensors-19-05467-s001.zip › Study with students/Survey (in spanish).pdf]

Desarrollo sencillo de apps móviles para IoT

¡Hola!

Bienvenido/a a la encuesta dirigida a evaluar la complejidad en el desarrollo de apps móviles para IoT.

Si tienes cualquier duda, por favor, no dudes en contactar conmigo mediante mi correo electrónico: ivan.ruiz@uca.es

No le llevará más de 5 minutos en completarla. Muchas gracias

*Required

Consentimiento

Doy mi consentimiento a los investigadores del grupo SPI-FM de la Universidad de Cádiz para recolectar las opiniones expresadas en la presente encuesta de evaluación. Este consentimiento sólo otorga permisos a dichos investigadores para la recolección anónima de datos y la posterior publicación de los mismos en foros de investigación.

El consentimiento puede ser revocado en cualquier momento, mediante correo electrónico dirigido a la dirección ivan.ruiz@uca.es

1. **¿Estás de acuerdo con las condiciones anteriores?** *

   Mark only one oval.

   - Sí
   - No *Stop filling out this form.*

Perfil de usuario

2. **Seleccione grupo** *

   Mark only one oval.

   - Grupo A
   - Grupo B

3. **Sexo** *

   Mark only one oval.

   - Mujer
   - Hombre

4. **Edad** *

5. **Tengo conocimientos tecnológicos para desarrollar pequeños juegos o aplicaciones utilizando entornos adaptados para el aprendizaje de programación, como Scratch, App Inventor o Alice, entre otros.** *

(1) Nada - (2) Poco - (3) Algo - (4) Bastante - (5) Mucho

Mark only one oval.

1	2	3	4	5
⬭	⬭	⬭	⬭	⬭

6. **Tengo conocimientos tecnológicos para implementar programas utilizando lenguajes como Java, Python, C, C#, Javascript, etc.** *

(1) Nada - (2) Poco - (3) Algo - (4) Bastante - (5) Mucho

Mark only one oval.

1	2	3	4	5
⬭	⬭	⬭	⬭	⬭

Conexión y desconexión Bluetooth con AppInventor/VEDILS

Observe y analice la siguiente figura. Posteriormente responda a la pregunta planteada

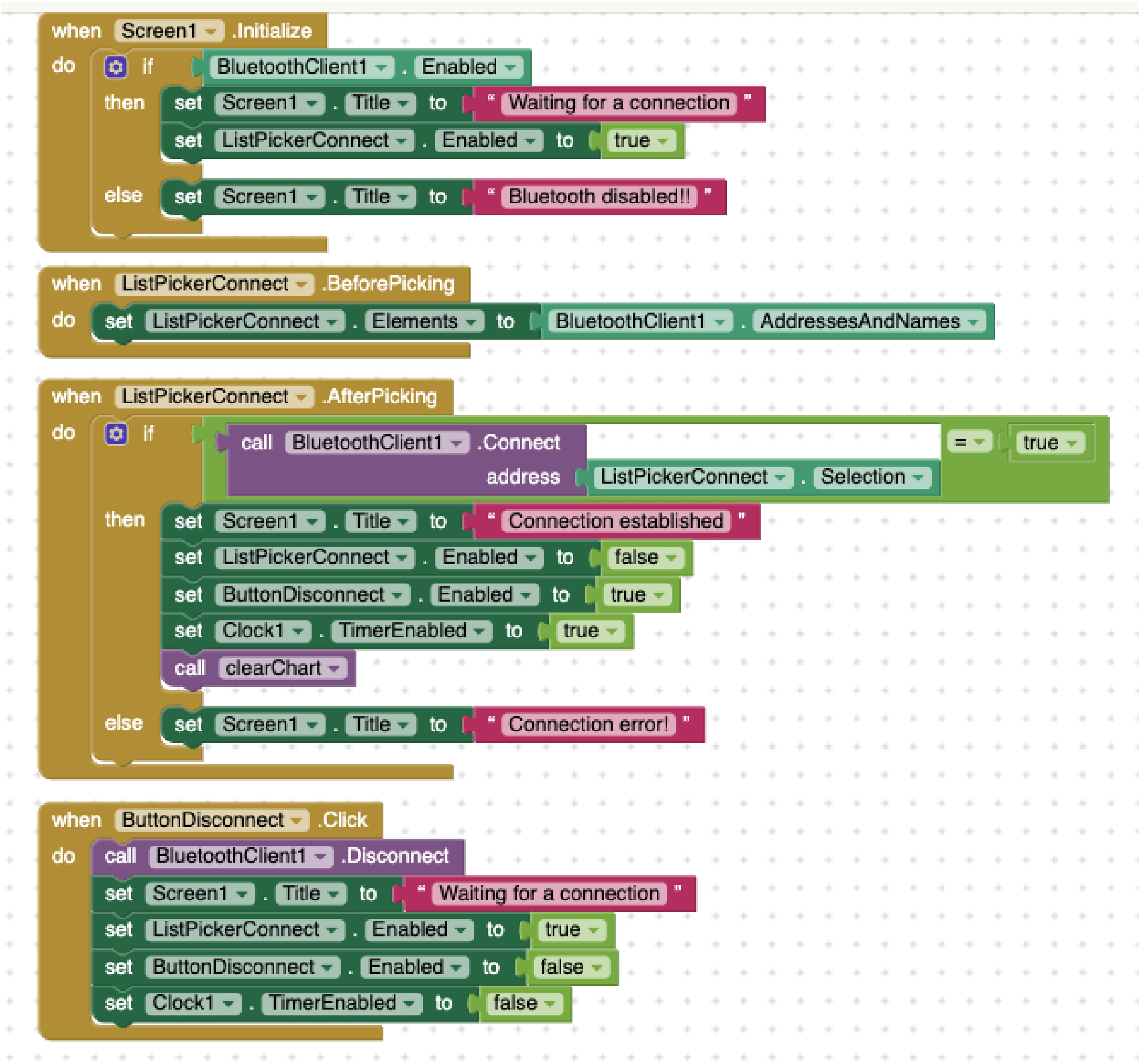

7. **Creo que con AppInventor/VEDILS resulta sencillo implementar la conexión/desconexión Bluetooth y el envío de datos entre la app móvil y el dispositivo IoT** *

Mark only one oval.

- ( ) Totalmente en desacuerdo
- ( ) En desacuerdo
- ( ) Ni de acuerdo ni en desacuerdo
- ( ) De acuerdo
- ( ) Totalmente de acuerdo

Captura y procesamiento de datos IoT con AppInventor

Observe y analice la siguiente figura. Posteriormente responda a la pregunta planteada

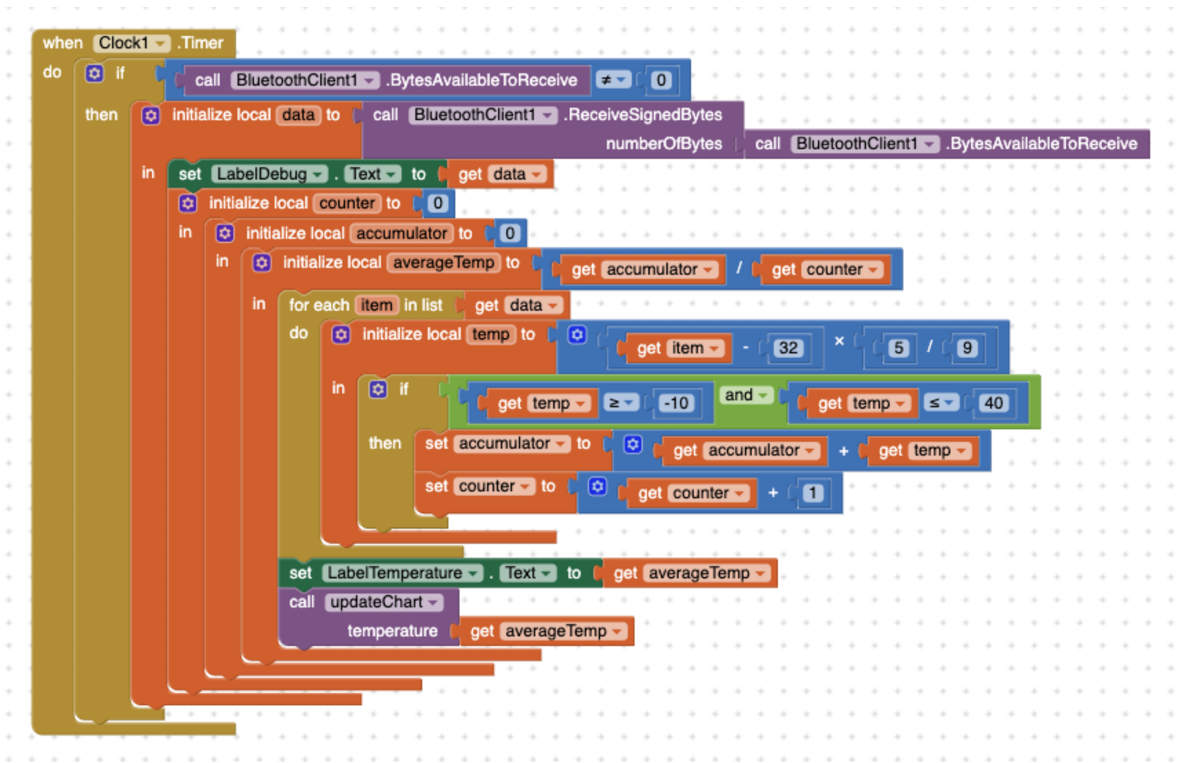

8. **Creo que con AppInventor resulta sencillo procesar los datos de un sensor IoT (temperatura), convertirlos (de grados Fahrenheit a grados centígrados), filtrarlos (eliminación de valores anómalos fuera del intervalo [-10,40]) y aplicar sobre ellos una función matemática (media aritmética)** *

Mark only one oval.

- ( ) Totalmente en desacuerdo
- ( ) En desacuerdo
- ( ) Ni de acuerdo ni en desacuerdo
- ( ) De acuerdo
- ( ) Totalmente de acuerdo

Captura y procesamiento de datos IoT con VEDILS

Observe y analice la siguiente figura. Posteriormente responda a la pregunta planteada

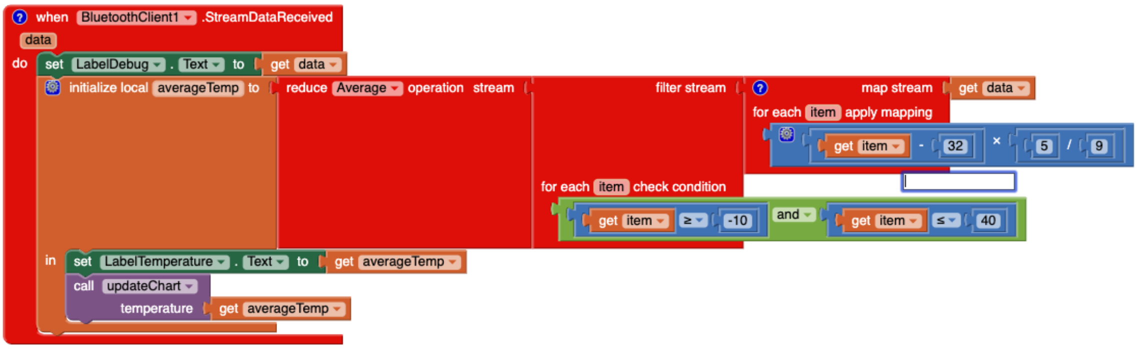

9. **Creo que con VEDILS resulta sencillo procesar los datos de un sensor IoT (temperatura), convertirlos (de grados Fahrenheit a grados centígrados), filtrarlos (eliminación de valores anómalos fuera del intervalo [-10,40]) y aplicar sobre ellos una función matemática (media aritmética)** *

Mark only one oval.

- Totalmente en desacuerdo
- En desacuerdo
- Ni de acuerdo ni en desacuerdo
- De acuerdo
- Totalmente de acuerdo

Visualización de datos IoT con AppInventor

Observe y analice la siguiente figura. Posteriormente responda a la pregunta planteada

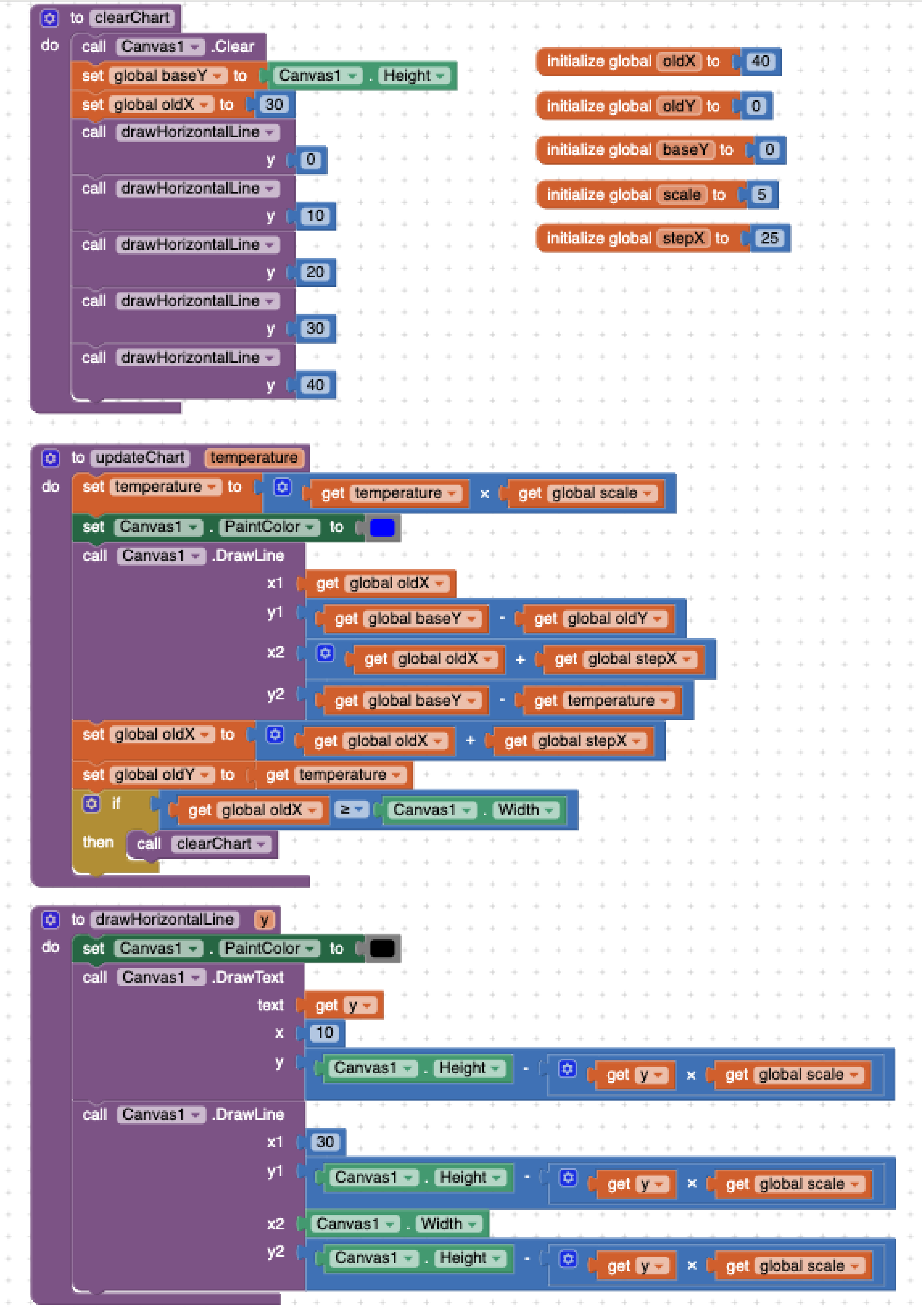

10. **Creo que con AppInventor resulta sencillo representar sobre una gráfica el histórico de datos procesados procedentes de un sensor IoT** *

Mark only one oval.

- ◯ Totalmente en desacuerdo
- ◯ En desacuerdo
- ◯ Ni de acuerdo ni en desacuerdo
- ◯ De acuerdo
- ◯ Totalmente de acuerdo

Visualización de datos IoT con VEDILS

Observe y analice la siguiente figura. Posteriormente responda a la pregunta planteada

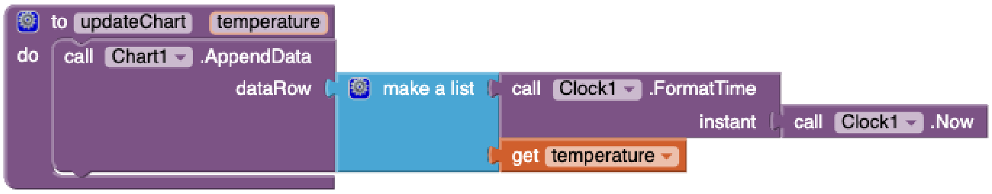

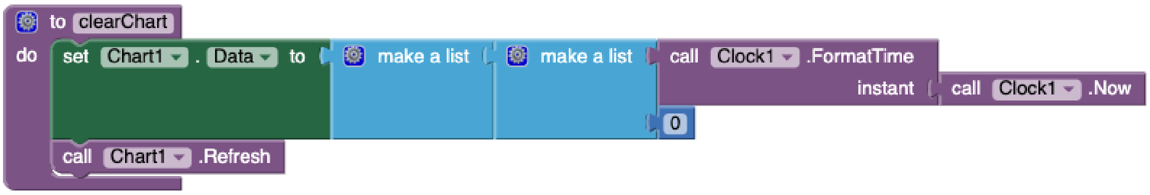

11. **Creo que con VEDILS resulta sencillo representar sobre una gráfica el histórico de datos procesados procedentes de un sensor IoT** *

Mark only one oval.

- Totalmente en desacuerdo
- En desacuerdo
- Ni de acuerdo ni en desacuerdo
- De acuerdo
- Totalmente de acuerdo

Comentarios finales

12. **En tu opinión, ¿cuáles son los beneficios que aporta AppInventor a la hora de desarrollar apps IoT?**

13. **En tu opinión, ¿cuáles son los beneficios que aporta VEDILS a la hora de desarrollar apps IoT?**

14. **Si tuvieras que desarrollar una aplicación IoT que consuma datos de algún sensor y los represente visualmente, ¿cuál de las dos herramientas elegirías?** *

Mark only one oval.

- AppInventor
- VEDILS
- Cualquiera de los dos sistemas. Me resultan igual de sencillos los dos

Powered by Google Forms
